# Cell Wall Remodeling in Abscission Zone Cells during Ethylene-Promoted Fruit Abscission in Citrus

**DOI:** 10.3389/fpls.2017.00126

**Published:** 2017-02-08

**Authors:** Paz Merelo, Javier Agustí, Vicent Arbona, Mário L. Costa, Leandro H. Estornell, Aurelio Gómez-Cadenas, Silvia Coimbra, María D. Gómez, Miguel A. Pérez-Amador, Concha Domingo, Manuel Talón, Francisco R. Tadeo

**Affiliations:** ^1^Centre de Genòmica, Institut Valencià d' AgràriesValència, Spain; ^2^Departamento de Biologia, Faculdade de Ciências, Universidade do PortoPorto, Portugal; ^3^Departament de Ciències Agràries i del Medi Natural, Universitat Jaume ICastelló de la Plana, Spain; ^4^Departamento de Desarrollo y Acción Hormonal en Plantas, Instituto de Biología Molecular y Celular de Plantas, Universidad Politécnica de Valencia-Consejo Superior de Investigaciones CientíficasValencia, Spain

**Keywords:** calyx abscission zone, cell wall modification, citrus fruit abscission, ethylene, lignin biosynthesis, phylogeny, transcriptomics

## Abstract

Abscission is a cell separation process by which plants can shed organs such as fruits, leaves, or flowers. The process takes place in specific locations termed abscission zones. In fruit crops like citrus, fruit abscission represents a high percentage of annual yield losses. Thus, understanding the molecular regulation of abscission is of capital relevance to control production. To identify genes preferentially expressed within the citrus fruit abscission zone (AZ-C), we performed a comparative transcriptomics assay at the cell type resolution level between the AZ-C and adjacent fruit rind cells (non-abscising tissue) during ethylene-promoted abscission. Our strategy combined laser microdissection with microarray analysis. Cell wall modification-related gene families displayed prominent representation in the AZ-C. Phylogenetic analyses of such gene families revealed a link between phylogenetic proximity and expression pattern during abscission suggesting highly conserved roles for specific members of these families in abscission. Our transcriptomic data was validated with (and strongly supported by) a parallel approach consisting on anatomical, histochemical and biochemical analyses on the AZ-C during fruit abscission. Our work identifies genes potentially involved in organ abscission and provides relevant data for future biotechnology approaches aimed at controlling such crucial process for citrus yield.

## Introduction

Abscission is a cell separation process by which plants can shed their aerial organs. It takes place in groups of functionally specialized cells known as abscission zones (AZs), which are located at specific sites of organ detachment in the plant (Roberts et al., 2002; Estornell et al., 2013; Tucker and Kim, 2015).

Abscission is a fundamental process in plant biology that represented a highly beneficial evolutionary adaptation for plants: abscission allows for discarding senescent or physiologically damaged organs and for highly efficient seed dispersal. However, from an agricultural point of view, abscission has a tremendous impact on yield, leading to high yield losses in key crops like brassica or citrus. In this way, understanding abscission at the molecular level is of top relevance not only to understand a fundamental process for plant physiology but also to generate new, improved, highly productive crops.

Abscission related traits (i.e., reduced abscission of fruits or seeds) are among the main agronomic traits selected along plant domestication (Konishi et al., 2006; Pickersgill, 2007). A current example is the expansion of late-season varieties of sweet orange in the citrus market. In such varieties, the decline in the fruit retention force is delayed during the maturing period in comparison with early and mid-season varieties (Gallasch, 1996) that usually undergo pre-harvest fruit abscission (Spiegel-Roy and Goldschmidt, 1996). Thus, late-season varieties of sweet orange extend the fruit harvesting season benefiting growers and food industry.

Control of abscission is also relevant to facilitate mechanical harvesting, thus reducing collection costs. Mechanical fruit harvesting systems have been developed although they are still inefficient and cause tree damages (Li et al., 2011). On the other hand, several abscission-triggering compounds have been used to improve mechanical harvesting. In citrus, treatments with CMNP (5-chloro-3-methyl-4-nitro-1*H*-pyrazole) are used to promote fruit loosening and to facilitate and coordinate mechanical harvesting of fruits (Burns, 2002). In this regard, understanding the mechanisms underlying abscission is essential to control abscission and improve harvesting practices and productivity.

Studies on floral organ abscission in the model system *Arabidopsis thaliana* have provided a wealth of valuable information. However, the current information about the molecular mechanisms underlying abscission in crop species is rather scarce.

Most of the molecular studies of abscission in crops have mainly been focused on the characterization of individual or few genes. However, high-throughput approaches have recently been applied in AZ-containing tissues of tomato flowers (Meir et al., 2010) and apple (Zhu et al., 2011), mature olive (Gil-Amado and Gomez-Jimenez, 2013; Parra et al., 2013), melon (Corbacho et al., 2013), litchi (Li et al., 2015), and orange fruits (Cheng et al., 2015). In our previous studies (Agustí et al., 2008, 2009, 2012), global expression analyses provided a wide set of genes potentially involved in citrus leaf abscission. These datasets included a number of cell wall modification related genes as well as genes involved in signaling, transcription control, protein synthesis and degradation and vesicle transport.

Our current challenge is to identify key regulatory genes of citrus fruit abscission which is, indeed, an economically important process. In citrus, maturing fruits are shed through the abscission zone C (AZ-C), located at the boundary between the calyx button and the fruit rind (FR). In this region, different tissues converge and the isolation of exclusive AZ-C cells for molecular studies without any contamination of other cell-types is extremely complicated. In this study, we have taken advantage of the optimization of laser microdissection (LM) in citrus tissues (Agustí et al., 2009; Matas et al., 2010; Caruso et al., 2012) for the accurate sampling of fruit AZ-C cells. This strategy has allowed the precise quantification of the timing and magnitude of gene expression and associate metabolites involved in the process of ethylene-promoted abscission in the specific cells of the AZ-C. Moreover, phylogenetic analyses of the most representative gene families during abscission in citrus and different plant species have revealed a link between phylogenetic proximity and expression pattern during this process suggesting highly conserved functions for specific members of these families in abscission. Overall, this study, through the identification of potential abscission-related genes and the detailed spatio-temporal analysis of the anatomical and histochemical changes in the activated AZ-C, provides crucial information for future biotechnological approaches aimed at improving citrus yield.

## Materials and methods

### Plant material and *in vitro* treatments

We used fruits from two *Citrus sinensis* cultivars: a mid-season orange cultivar (cv. Washington Navel) that usually undergoes pre-harvest abscission and a late-season orange cultivar (cv. Ricalate Navel) with delayed abscission. Maturing fruits were harvested after color change from adult trees grown in a homogeneous experimental orchard under normal cultural practices at the Institut Valencià d'Investigacions Agràries (IVIA). Fruits were separated from the tree leaving 2 cm peduncles to isolate the AZ-C for further analyses. For abscission kinetics studies and tissue collection, Washington Navel fruits were incubated for 0, 24, 48, and 96 h in the presence or absence of ethylene (10 μL/L) in sealed 10 l containers at 22°C with a 16 h light period under fluorescent lighting. Ricalate Navel fruits were incubated for 0, 24, 48, 96, and 192 h in the presence of 1-aminocyclopropane-l-carboxylic acid (ACC; 0.1 mM) or water under the same temperature and light conditions. In this case, a 3 mL Pasteur pipette containing the ACC solution or water was fitted to the fruit peduncles.

### Phloroglucinol staining

Phloroglucinol staining for lignin in fresh cut tissue portions (0.5 cm^3^) containing the AZ-C after 0, 24, and 48 h of ethylene or ACC treatment was performed according to Tadeo and Primo-Millo (1990). Samples were cut longitudinally to allow AZ-C staining and for further image acquisition. A saturated solution of phloroglucinol (Sigma-Aldrich) in 20% HCl was directly applied to samples. Observation was carried out with an Olympus SZ61 stereomicroscope (Olympus GmbH).

### Cryoscanning electron microscopy (cryo-SEM)

Longitudinal sections as well as the proximal (peduncle) and distal (fruit) fracture plane of the ethylene-promoted AZ-C were observed using cryo-SEM. To examine longitudinal sections of the AZ-C, 1 cm portions of tissue were manually dissected with a razor blade. In the second case, the peduncle was forcibly separated from the fruit. Specimen mounting and AZ-C observation were carried out as previously described in Agustí et al. (2009). At least three samples containing the AZ-C after 24, 48, and 96 h of ethylene treatment were observed.

### Periodic acid-schiff (PAS) staining

Tissue containing the AZ-C after 0, 24, and 48 h of ACC treatment was manually dissected using a razor blade in 0.5 cm^3^ portions. These samples were fixed overnight at 4°C in a 4% (w/v) paraformaldehyde-PBS solution. After fixation, samples were washed with PBS, dehydrated in a graded ethanol series and embedded in LR White (Electron Microscopy Sciences). Longitudinal sections of the calyx button area (1 μm thickness) were cut with a Leica RM2165 microtome and placed on glass slides. Slides were further stained with PAS (Sigma-Aldrich) and mounted with DPX Mountant (Fluka). Observations were performed on a Leica DMLA microscope (Leica Microsystems) and images were processed with Leica ASMLD Version 4.0 software.

### Preparation of tissue containing the AZ-C for LM

Portions of tissue containing the AZ-C (0.5 cm^3^) were dissected from fruits after 0, 12, and 24 h of ethylene treatment for the transcriptomics assay, and after 0, 12, 24, and 36 h of ACC treatment for lignin intermediates quantification. Preparation of cryosections and microdissection were performed as previously described in Agustí et al. (2009). Cells from the AZ-C and the adjacent FR were selected from 30 to 40 cryosections and collected separately.

### Phloroglucinol staining of cryosections

Cryosections of 14 μm of tissue containing the AZ-C after 48 h of ethylene treatment were processed as described in Agustí et al. (2009) and mounted on CryoJane® adhesive coated slides (Instrumedics) following the manufacturer's instructions. Slides were stored at −80°C until phloroglucinol staining. Staining for lignin was performed using a saturated solution of phloroglucinol (Sigma-Aldrich) in 20% HCl. Observation was carried out with an Olympus SZ61 stereomicroscope (Olympus GmbH).

### RNA isolation, sample labeling, and microarray hybridization

Three independent biological replicates were collected for each cell type at 0, 12, and 24 h after ethylene treatment. For each independent sample, total RNA from ~40,000 pooled cells was extracted using the RNeasy Micro Kit (Qiagen) following the manufacturer's instructions. The RNA purity was assessed by measurements of OD260/OD280. Two RNA amplification rounds were performed utilizing the TargetAmp™ 2-Round Aminoallyl-aRNA Amplification Kit (EPICENTER) according to the manufacturer's instructions to synthesize the antisense cRNA. The quality of the amplified RNA was evaluated by OD260/OD280 measurements and agarose gel electrophoresis. Each sample was labeled with Cy5 and co-hybridized with Cy3-labeled antisense cRNA from a reference sample containing a mixture of equal amounts of RNA from all experimental samples (0, 6, 12, 24, 48, and 96 h of ethylene/air treatment). RNA labeling, microarray hybridization, and slide washes were performed as previously described in Cercos et al. (2006). Hybridized microarrays scanning, hybridization data acquisition, and microarray normalization and analysis were carried out as described in Agustí et al. (2009). A cDNA microarray including 21.081 putative genes of citrus was utilized (Martinez-Godoy et al., 2008). Gene expression differences were considered significant under a *P*-value lower than 0.05 and an M contrast cutoff value of +0.5 or −0.5. In this work, a time course experiment was designed for each cell type (AZ-C and FR), therefore, the expression level corresponds to M = log_2_ [AZ-C_t_/AZ-C_0_] or M = log_2_ [FR_t_/FR_0_]. The raw microarray data as well as the protocols used to produce the data and the normalized data were deposited in the ArrayExpress database under the accession number E-MTAB-4538. Functional classification of the selected genes was performed using MIPS (Munich Information Center for Protein Sequences, http://www.helmholtzmuenchen.de/en/mips/) categorization. Amplified RNA was used for the validation of microarray hybridization data by semi-quantitative RT-PCR (sqRT-PCR) analysis (Figure S1).

### sqRT-PCR analysis

sqRT-PCR analysis was carried out using the SuperScript II Reverse Transcriptase kit (Invitrogen, Carlsbad) following the manufacturer's instructions. After first-strand cDNA synthesis, PCR reactions were performed using the Biotools Taq DNA Polymerase (BIOTOOLS, B&M Labs). Size and intensity of expected bands were checked by 1% agarose gel electrophoresis. Citrus *UBC* gene (Ubiquitin-conjugating enzyme) was used as a reference to evaluate the amounts of mRNA in each sample. Primer sequences are available in Table S1.

### *In situ* hybridization

RNA *in situ* hybridization with digoxigenin-labeled probes was performed as described (Gomez et al., 2011). Portions of tissue containing the AZ-C (0.5 cm^3^) were dissected from fruits after 24 h of ethylene treatment and immediately fixed at 4°C overnight in FAE (25% formaldehyde, 5% acetic acid, 50% ethanol), dehydrated, embedded in paraffin wax and sectioned to 8 μm. For *CitCEL6* and *CitPG20*, RNA antisense and sense probes were generated with SP6 and T7 RNA polymerases, using as substrate a 1518 bp fragment of the *CitCEL6* cDNA (1–1518 from ATG) or a 1110 bp fragment of the *CitPG20* cDNA (217–1326 from ATG), amplified by PCR and cloned into the pGEM-T Easy vector (Promega).

### Lignin intermediates quantification

Coumaric acid, caffeic acid, and ferulic acid were analyzed by UPLC coupled to tandem mass spectrometry (UPLC-MS/MS) as described by Argamasilla et al. (2014). Three independent samples containing ~40,000 pooled AZ-C cells were isolated by LM for each time point of ACC treatment (0, 12, 24, and 36 h) and collected in 60 μL of water.

### Sequence identification, alignment, and phylogenetic analysis

Members of the different gene families associated with cell wall remodeling in citrus (based on CAZy classification; Carbohydrate-Active Enzymes; Cantarel et al., 2009; http://www.cazy.org/) were identified by TBLASTN search in the *Citrus clementina* haploid genome (version 0.9) database web browser (http://www.phytozome.net/search.php) using the consensus sequence for the catalytic domain of each family. Prediction of characteristic domains and conserved motifs was carried out through SMART (http://smart.embl-heidelberg.de/; Schultz et al., 1998; Letunic et al., 2009), PSORT (http://psort.hgc.jp/form.html), InterProScan (http://www.ebi.ac.uk/Tools/pfa/iprscan/) and big-PI Plant Predictor (http://mendel.imp.ac.at/gpi/plant_server.html) servers. Phylogenetic trees are based on multiple alignments using the profile alignment function of ClustalW (www.ch.embnet.org/software/ClustalW-XXL.htm) and were generated with MEGA7 (Kumar et al., 2016) using the neighbor-joining algorithm with 1000 bootstrap replicates. Poisson correction for multiple substitutions was used and only values higher than 50% were considered.

### Immunolocalization of pectic polyssacharides

The primary monoclonal antibodies (mAbs) used in this study and provided by Prof. Paul Knox (Centre for Plant Sciences, Faculty of Biological Sciences, University of Leeds, UK) were LM5 [anti-(1,4)-β-D-galactan; Jones et al., 1997], LM6 [anti-(1,5)-α-L-arabinan; Willats et al., 1998] and JIM5 [anti-partially methylesterified/de-esterified homogalacturonan; (Knox et al., 1990)]. The secondary antibody was fluorescein isothiocyanate (FITC)-conjugated anti-rat IgG (Sigma-Aldrich). Immunolocalization of pectic polysaccharides was performed on semi-thin sections of tissue containing the AZ-C (0.5 cm^3^) from fruits after 0, 24, and 48 h of ACC treatment. Immunolocalization of pectic polyssacharides, light microscopy and image acquisition were perfomed as described in Coimbra et al. (2007).

## Results and discussion

### Ethylene accelerates citrus fruit abscission

**Figure 1:**
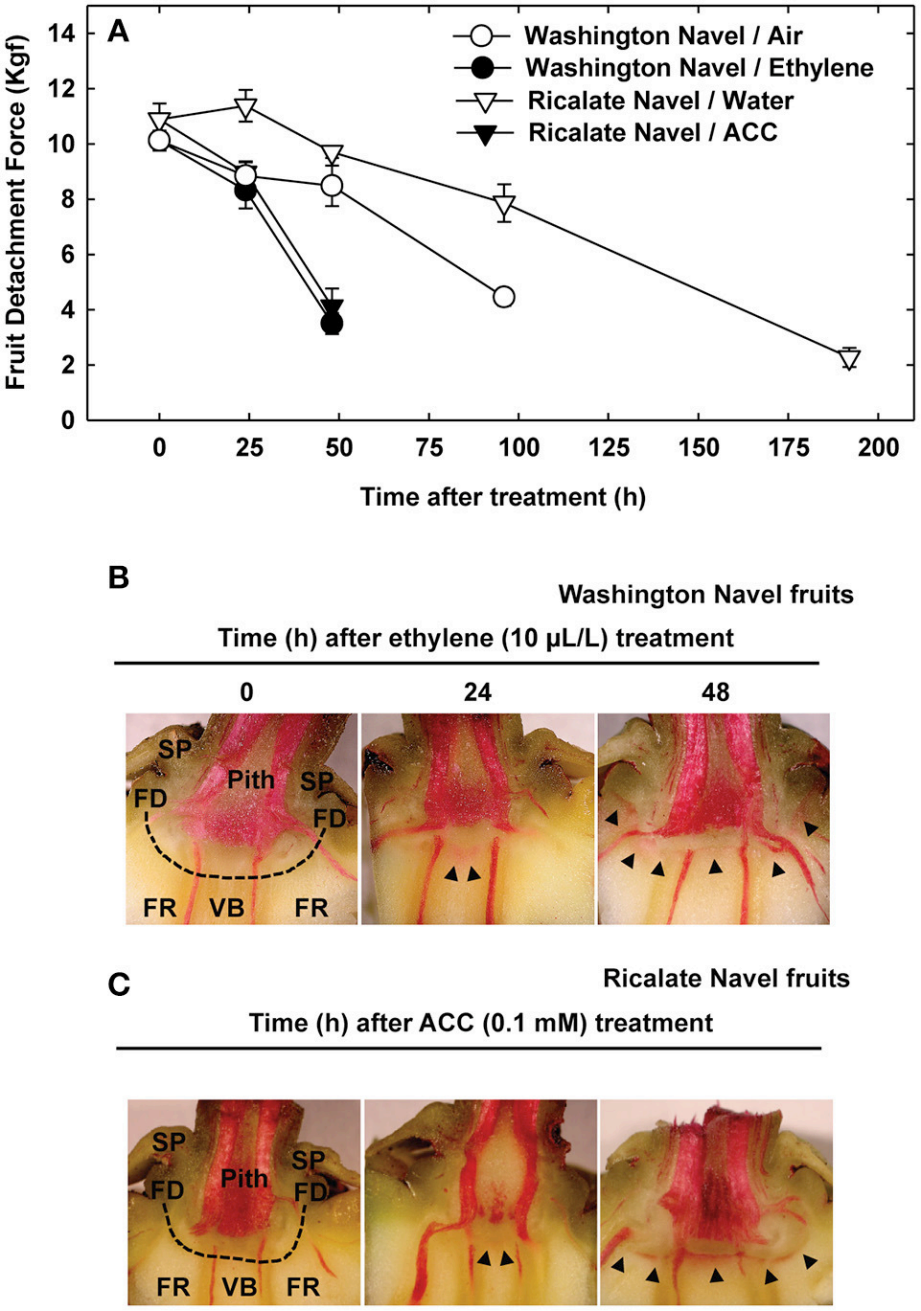
**Effect of ethylene and 1-aminocyclopropane- 1-carboxylic acid (ACC) on citrus fruit abscission. (A)** Abscission kinetics of Washington Navel fruits non-treated or treated with ethylene and Ricalate Navel fruits non-treated or treated with ACC. The results are means of 10 fruits ± SE. **(B,C)** Phloroglucinol staining for lignin in the AZ-C of Washington Navel fruits after ethylene treatment **(B)** and Ricalate Navel fruits after ACC treatment **(C)**. Dashed line, abscission zone C; 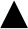, lignin deposition (phloroglucinol); FR, fruit rind; FD, floral disc; SP, sepals; VB, vascular bundles; P, parenchyma.

We performed a kinetics assay of citrus fruit abscission in response to abscission-accelerating treatments to determine the optimal sampling for the transcriptomic analysis. To that end, we carried out a comparison between orange (*C. sinensis*) fruits incubated with ethylene or its immediate metabolic precursor 1-aminocyclopropane-1-carboxylic acid (ACC) and fruits incubated with air or water (controls). We used maturing fruits from a mid-season orange cultivar (cv. Washington Navel) that usually undergoes pre-harvest abscission and from a late-season orange cultivar (cv. Ricalate Navel) with delayed abscission. We observed a faster decrease of fruit detachment force (FDF) in both Washington Navel and Ricalate Navel fruits treated with ethylene/ACC in comparison to air-/water-treated control fruits (Figure 1A). At 48 h after treatment, the FDF in fruits treated with ethylene or ACC was around 4 kgf. However, control fruits of Washington Navel and Ricalate Navel only showed values of FDF around 4 kgf at 96 and 192 h, respectively, a response that matches their pre-harvest abscission behavior. Thus, ethylene and ACC accelerated the abscission process in both varieties tested. This result strongly suggests that the natural delay in the schedule of FDF decline in fruits of the late-season variety Ricalate Navel in comparison with those of the mid-season variety Washington Navel was not related to any impairment in the response of well-developed tissues to ethylene (Zacarias et al., 1993). Based on these findings, we used induced AZ-C samples from both varieties for further analyses.

### Phloroglucinol staining reveals a positive correlation between abscission and lignin deposition

Phloroglucinol staining in receptacles of both Washington Navel and Ricalate Navel fruits revealed lignin deposition at the central core of the AZ-C, between the axial vascular bundles, 24 h after ethylene and ACC treatments (Figures 1B,C). Forty-eight hours after the treatments, lignin deposition spread out along the AZ-C, perfectly drawing the separation line between the calyx button and the FR. Accordingly, timing for lignin deposition positively correlated with abscission kinetics.

### Morphological changes in activated AZ-C cells

**Figure 2:**
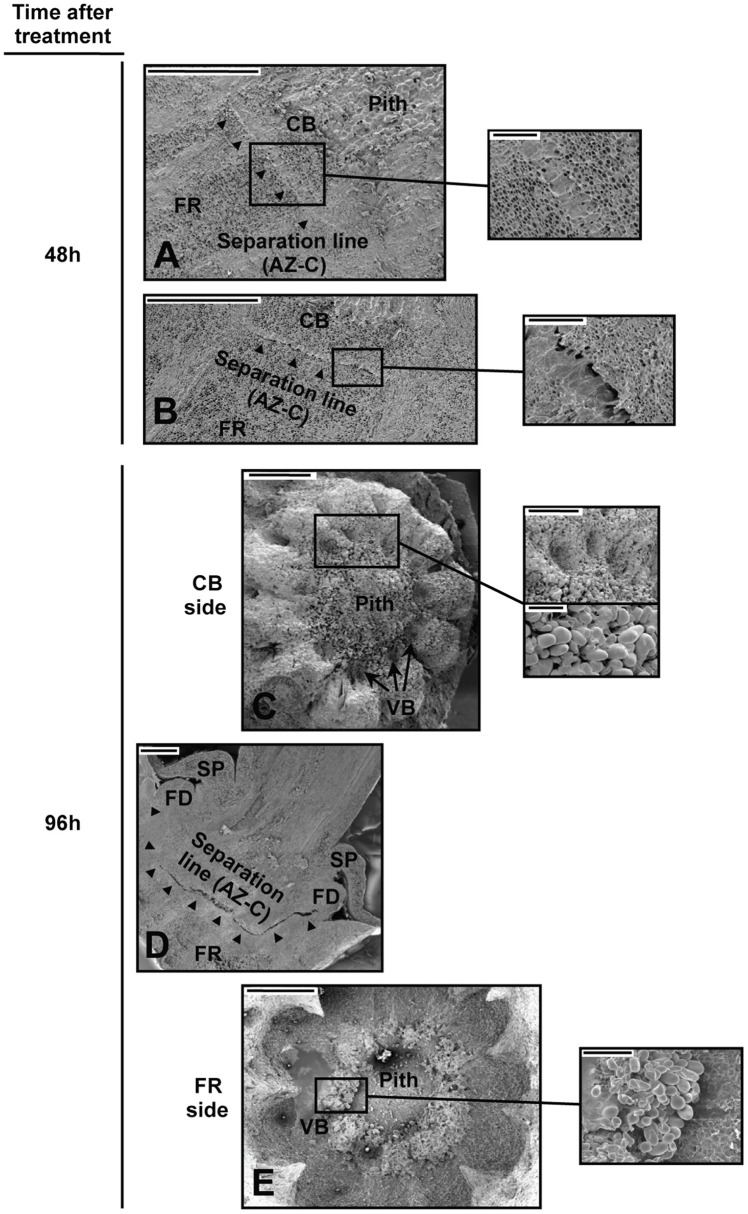
**Cellular morphology of the AZ-C. (A–E)** Scanning electron micrographs of longitudinal sections **(A,B,D)** and the proximal (**C**; calyx button side) and distal (**E**; fruit rind side) fracture planes of the AZ-C from Washington Navel fruits after 48 h **(A,B)** and 96 h **(C–E)** of ethylene treatment. High magnification pictures show cells of separation layers. AZ-C, abscission zone C; 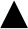, separation line inside the AZ-C; CB, calyx button; FD, floral disc; FR, fruit rind; SP, sepal; VB, vascular bundles. Scale bars: 1 mm **(A–E)**, 500 μm **(A–C)**, 200 μm **(E)**, 100 μm **(C)**.

We used scanning electron microscopy (SEM) to examine changes in the cellular morphology of the AZ-C from Washington Navel fruits treated with ethylene (Figure 2). We observed the first cellular signs of activation of abscission by ethylene in the central core of the AZ-C at 48 h of treatment (Figures 2A,B). At that time, AZ-C samples could be split into two groups, one showing early stages of cell separation and the other one showing late events of cell separation. In the former group, the AZ-C was clearly distinguishable (Figure 2A), with accumulation of an amorphous material probably derived from the partial dissolution of the middle lamella and cell wall of the AZ-C cells. In the latter group of samples, cell separation was observed in the central core of the AZ-C (Figure 2B). A greater accumulation of amorphous material was observed, suggesting that cell wall and middle lamella degradation was complete after 48 h at the central region. At 96 h after ethylene treatment, cell separation extended from the central core to the periphery of the AZ-C (Figure 2D) and differential cell expansion was observed at proximal (calyx button) and distal (fruit) sides (Figures 2C,E). At the proximal side, parenchymatic pith cells underwent expansion (Figure 2C) while, at the distal side, expansion occurred in the cells of the axial vascular bundles (Figure 2E).

### Two different cell areas form the AZ-C

**Figure 3:**
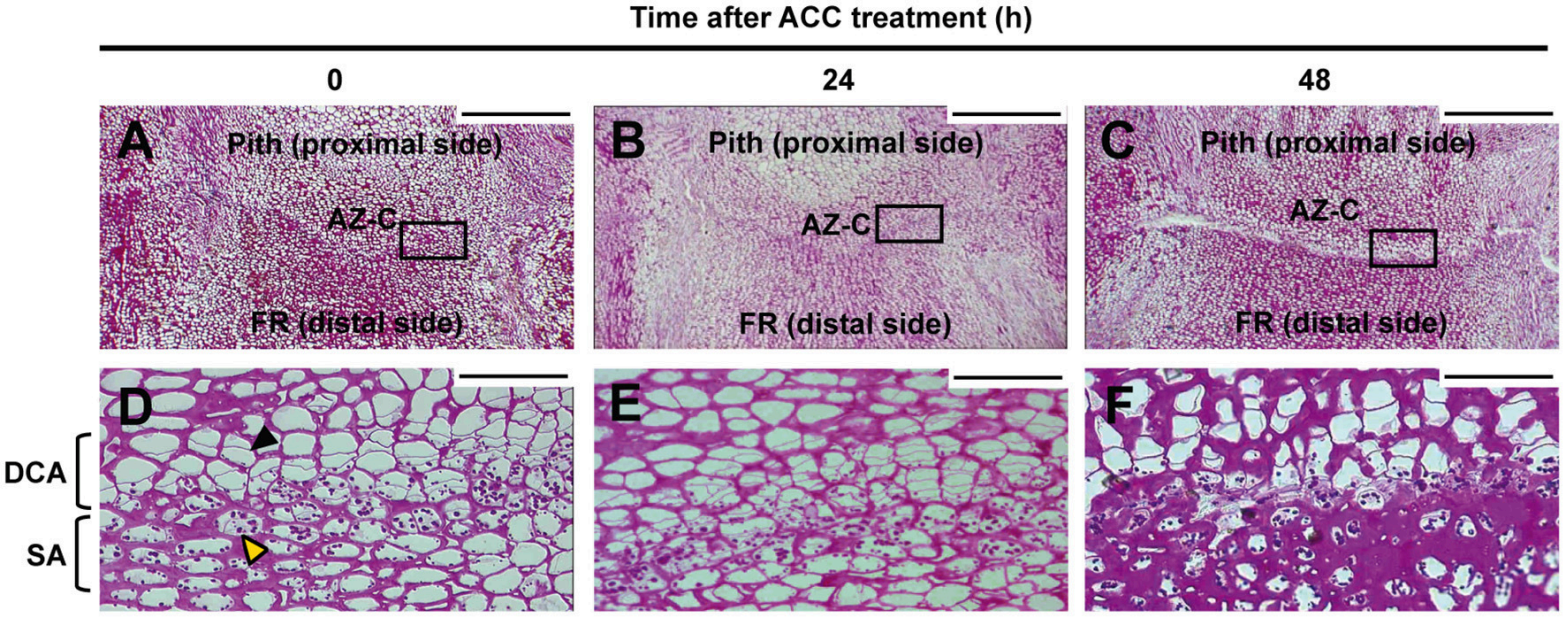
**Anatomy of the AZ-C**. Periodic acid-Schiff reactive (PAS) staining for insoluble carbohydrates of longitudinal sections of the AZ-C from Ricalate Navel fruits non-treated **(A,D)** and treated for 24 h **(B,E)** and 48 h **(C,F)** with ACC. Squares in **(A–C)** show the area magnified with the 40X objective. AZ-C, abscission zone C; FR, fruit rind; DCA, divided cells area; SA, starch-rich area; 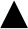, recently divided cell; 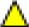, cell containing amyloplasts. Scale bars: 500 μm and 50 μm.

Periodic acid-Schiff (PAS) staining was used to characterize anatomically the AZ-C after ACC treatment (Figure 3). This method detects insoluble carbohydrates and was used to distinguish the cells belonging to the AZ-C since these accumulate starch grains (Wilson and Hendershott, 1968; Huberman et al., 1983; Shiraishi and Yanagisawa, 1988; Goren, 1993). In addition to the starch-rich cell area (SA) previously identified by Wilson and Hendershott (1968) at the distal side of the AZ-C (FR side), we identified another cell area located at the proximal side of the AZ-C (pith side) and composed by recently divided cells based on the observation of thinner cell walls formed between cells (Divided Cells Area, DCA; Figure 3D). Then, the AZ-C was constituted by 10–15 cell layers distributed in cellular areas with two different cell morphologies and organellar composition (i.e., cells from the SA contain amyloplasts). The analysis of the AZ-C after 48 h of treatment suggests that cell wall degradation and cell degeneration occurred mainly at the layers of the SA in the fracture plane, adjacent to the mesocarp cells of the FR known as the albedo (see **Figure 9**). However, cell expansion occurred at the DCA (Figure 3F). These results together with kinetics (Figure 1A), phloroglucinol staining (Figures 1B,C) and SEM (Figure 2) data suggested that the activation of the fruit AZ-C by ethylene/ACC occurred early after treatment, probably prior to 24 h. The events related to cell wall loosening might start at 24 h, while cell separation seemed to begin at 48 h and to be completed at 96 h after treatment.

### Gene expression regulated by ethylene in AZ-C and FR cells

For gene expression analysis, we used the 20 K citrus cDNA microarray (Martinez-Godoy et al., 2008). We isolated cells from the central core of the AZ-C as well as from the FR located beneath the AZ-C through LM (Figure S2) to perform a time-course experiment (0, 12, and 24 h-ethylene) and compared data from each analysis. Results showed that ethylene differentially regulated 2280 genes exclusively in the AZ-C cells, 1742 genes exclusively in the FR cells, and 2001 genes were regulated by ethylene in both the AZ-C and the FR cells (Table S1).

All differentially regulated genes were grouped into functional categories according to the Munich Information Center for Protein Sequences (MIPS). The categories sugar, glucoside, polyol, and carboxylate metabolism and polysaccharide metabolism showed a higher percentage of regulation in the AZ-C compared with the FR (Table S2).

The set of genes discussed below was selected because of its prominent representation in the AZ-C or its particular biological interest. These gene families included those associated with cell wall metabolism and monolignol biosynthesis and polymerization (Table S3).

### Many genes related to cell wall modification are regulated during fruit abscission

A high number of genes encoding cell wall modification enzymes were differentially regulated by ethylene exclusively in the fruit AZ-C. Our data suggested that this strong activation of cell wall metabolism occurred through both degradation and biosynthesis (Table 1) as we previously showed in the laminar abscission zone (LAZ) during leaf abscission (Agustí et al., 2008, 2009, 2012). Genes encoding enzymes that hydrolyze the cell wall and middle lamella would be responsible of the cell wall disassembly observed mainly in the SA at the distal side of the AZ-C (Figure 3F), which would lead to the effective organ separation. On the other hand, genes involved in the biosynthesis of new cell wall components would be related to the cell expansion observed mainly in the cell layers of the DCA located at the proximal side of the fracture plane (Figures 2C, 3F). The cell expansion might be associated with the generation of protective layers at the regions of the receptacle that remain exposed to the environment after abscission. Indeed, the recently divided cells at the proximal side of the AZ-C did not show cell degeneration (Figure 3F), suggesting that these cells might acquire such a function during the last step of organ abscission (Roberts et al., 2002; Estornell et al., 2013).

**Table 1:** **Relative gene expression values (AZ-Ct vs. AZ-C0) of genes involved in cell wall modification exclusively regulated by ethylene in AZ-C cells**.

**Name**	**Contig/singleton ID**	**Microarray probe**	**Putative Ath orthologue**	**Relative expression [log2 (AZ-Ct/AZ-C0)]**	
				**12 h**	**24 h**
**ENDO-1,4-**β**-GLUCANASES|CELLULASES (GH9s)**					
*CitCEL3*	aCL1687Contig1	IC0AAA38AD03	AT2G32990	–	0.69
CitCEL6^*^	aCL1347Contig1	C21007H10	AT4G02290	–	4.15
*CitCEL10*	aCL7597Contig1	IC0AAA68DE06	AT4G02290	–	0.60
*CitCEL17*	aCL1288Contig1	C32011E04	AT1G75680	−0.88	–
*CitCEL22*	aC20010F01SK_c	C20010F01	AT1G23210	–	0.72
**POLYGALACTURONASES (GH28s)**					
*CitPG6*	aCL2029Contig1	C03009E03	AT4G23820	–1.58	–
*CitPG16*	aCL5261Contig1	C01018A12	AT3G61490	1.02	–
CitPG20^*^			AT3G07970	*in-situ* hybridization	
*CitPG41*	aC18008C05Rv_c	C18008C05	AT3G57790	1.10	1.49
	aCL1063Contig1	IC0AAA19CA02	AT3G57790	0.88	0.95
*CitPG42*	aIC0AAA85AB02RM1_c	IC0AAA85AB02	AT3G48950	–	0.63
*CitPG43*	aCL675Contig4	IC0AAA67DG09	AT2G43870	–	2.34
**PECTATE-LYASES (PL1s)**					
*CitPL1*	aC03011D06SK_c	C03011D06	AT5G63180	−0.86	−0.80
*CitPL5*	aIC0AAA15AF11RM1_c	IC0AAA15AF11	AT1G67750		2.66
**PECTIN-METHYLESTERASES (CE8s)**					
*CitPME8*	aC05807A09SK_c	C05807A09	AT4G33220	−3.13	−2.57
*CitPME11*	aCL1691Contig1	C08033H07	AT1G11580	−2.30	–
*CitPME13*	aCL4116Contig2	C01011H09	AT5G53370	−2.15	−2.90
*CitPME24*	aCL1451Contig1	IC0AAA40DF03	AT1G69940	0.71	0.71
*CitPME41*	aCL2379Contig1	C32102B03	AT5G09760	–	1.41
**PECTIN-ACETYLESTERASES (CE18s)**					
*CitPAE1*	aKN0AAP13YN19FM1_c	KN0AAP13YN19	AT3G62060	−0.72	–
*CitPAE4*	aCL67Contig4	C08028G04	AT4G19420	0.58	0.51
*CitPAE6*	aCL7344Contig1	C02003B05	AT5G26670	−1.57	–
	aKN0AAI1DH10FM2_c	KN0AAI1DH10	AT5G26670	−2.07	–
β**-GALACTOSIDASES (GH35s)**					
*CitGBAL16*	aC31805H10EF_c	C31805H10	AT4G36360	−1.59	
	aCL7104Contig1	C02004B02	AT4G36360	−1.01	
β**-GALACTOSIDASES (GH2s)**					
CitGH22	aCL4443Contig1	C31401H10	AT3G54440	0.71	0.95
β**-GLUCOSIDASES (GH1s)**					
*CitBGLU17*	aCL5744Contig1	C31007D10	AT2G44480	−0.59	−0.55
*CitBGLU24*	aCL1136Contig3	IC0AAA1CB06	AT3G06510	–	0.62
β**-MANNOSIDASES (GH5s)**					
*CitMAN4*	aC04002G09SK_c	C04002G09	AT1G02310	1.41	–
**XYLOGLUCAN ENDOTRANSGLYCOSYLASES/HYDROLASES (GH16s)**					
*CitXTH16*	aC02023G10SK_c	C02023G10	AT4G03210	−1.55	−2.33
*CitXTH24*	aIC0AAA99CH05RM1_c	IC0AAA99CH05	AT1G32170	1.07	1.03
*CitXTH28*	aCL6772Contig1	C01009B04	AT4G37800	–	−0.88
α**-XYLOSIDASES (GH31s)**					
*CitXYL4*	aCL6235Contig1	C05075C11	AT1G68560	−0.98	–
β**-XYLOSIDASES (GH3s)**					
*CitBXL13*	aCL3345Contig1	C02024D10	AT1G78060	−0.63	–
*CitBXL16*	aCL8110Contig1	IC0AAA75AA10	AT5G20950	−0.99	–
**EXPANSINS**					
*CitEXP14*	aCL2131Contig1	IC0AAA14BD04	AT2G40610	–	3.01
	aIC0AAA87BH09RM1_c	IC0AAA87BH09	AT2G40610	1.63	3.19
*CitEXP15*	aKN0AAQ1YG09RM1_c	KN0AAQ1YG09	AT4G17030	1.69	1.40
*CitEXP19*	aC02006G07SK_c	C02006G07	AT1G20190	–	0.75
**CELLULOSE SYNTHASES/CELLULOSE SYNTHASE-LIKE PROTEINS**					
*CitCes1*	aC16012C03SK_c	C16012C03	AT4G24010	1.82	–
*CitCes2*	aIC0AAA5DG11RM1_c	IC0AAA5DG11	AT5G05170	−1.74	–
*CitCsl3*	aCL5293Contig1	C05070F01	AT3G03050	–	0.68
**UDP-GLUCOSE 4-EPIMERASE**					
*CitUGE1*	aC31108G08EF_c	C31108G08	AT4G10960	–	0.95
*CitUGE2*	aCL6604Contig1	C03009D04	AT1G12780	–	0.82
**MANNAN SYNTHASES**					
*CitManS1*	aCL3377Contig1	IC0AAA16BH03	AT5G22740	–	1.47
*CitManS2*	aCL3377Contig2	IC0AAA99AD02	AT5G22740	–	1.36
**GALACTOMANNAN GALACTOSYLTRANSFERASE (GMGT)**					
*CitGMGT1*	aC34004D03EF_c	IC0AAA58DH01	AT2G22900	–	1.35
**RHAMNOSE BIOSYNTHETIC ENZYME**					
*CitRHM1*	aCL4478Contig1	KN0AAI3AD11	AT1G78570	−0.52	–
**GLUCOSYLTRANSFERASES**					
*CitGTF1*	aCL3010Contig2	IC0AAA58BE08	AT1G77130	0.82	–
*CitGTF2*	aCL1592Contig1	IC0AAA30DE02	AT1G16570	0.58	–
*CitGTF3*	aCL3054Contig2	KN0AAK3DE03	AT3G50060	–	1.21
*CitGTF4*	aCL6931Contig1	IC0AAA42BE09	AT3G25140	–	0.65
*CitGTF5*	aCL5570Contig1	C05056H08	AT1G77990	−1.42	−1.51
*CitGTF6*	aCL6758Contig1	C31701H10	AT3G02100	–	−1.23
*CitGTF7*	aKN0AAB3DB09ZM1_c	KN0AAB3DB09	AT3G45400	–	0.60
**GDP-L-FUCOSE SYNTHASE**					
*CitGLFS1*	aCL790Contig1	IC0AAA85AB07	AT1G17890	–	0.73
**GALACTOSIDE 2-ALPHA-L-FUCOSYLTRANSFERASE**					
*CitGLFT1*	aIC0AAA69BA06RM1_c	IC0AAA69BA06	AT1G74420	–	0.58
*CitGLFT2*	aCL5210Contig1	C08029G10	AT1G05575	−0.87	−1.24
**UDP-GLUCOSE DEHYDROGENASE**					
*CitUGD1*	aKN0AAP5YD20FM1_c	KN0AAP5YD20	AT5G15490	–	0.51
**GLUCURONOSYL TRANSFERASE-LIKE PROTEIN**					
*CitGluT1*	aCL8573Contig1	C02015B05	AT3G55700	–	−0.54
**UDP-GLUCURONATE DECARBOXYLASE**					
*CitUGluD1*	aCL1799Contig2	C02011A11	AT2G28760	1.67	1.57
**MANNOSYLTRANSFERASE-LIKE PROTEIN**					
*CitManT1*	aIC0AAA25BC01RM1_c	IC0AAA25BC01	AT2G27100	0.77	0.60

–, No significant regulation. (^*^) Localization of gene expression in AZ-C cells by in-situ hybridization. Putative gene identifications are based on sequence homology with Arabidopsis thaliana. Additional data are shown in Figures S3–S6.

### Phylogenetic analyses reveal convergences between cell wall-remodeling proteins

Cell wall-remodeling enzymes are key players in abscission, but also in many other biological processes. Thus, the identification of abscission specific genes within these gene families acquires special relevance. However, this task becomes complicated due to the large size and high functional diversification of such gene families. To identify potential abscission specific cell wall-remodeling enzymes as well as reinforce the gene expression data, a phylogenetic analysis of gene families related to cell wall modification was carried out. For this purpose, genes encoding cell wall-remodeling proteins in the sequence of the *C. clementina* haploid genome (http://www.phytozome.net; Wu et al., 2014) were retrieved and compared to those of orange (*C. sinensis*; http://citrus.hzau.edu.cn/orange/) to identify all members of these citrus gene families (Table S3). We considered the phylogenetic relationships between cell wall-remodeling proteins of citrus and *Arabidopsis* along with proteins previously described as associated with abscission or dehiscence in other plant species.

#### Glycoside hydrolases and pectate lyases

##### Cellulases

**Figure 4:**
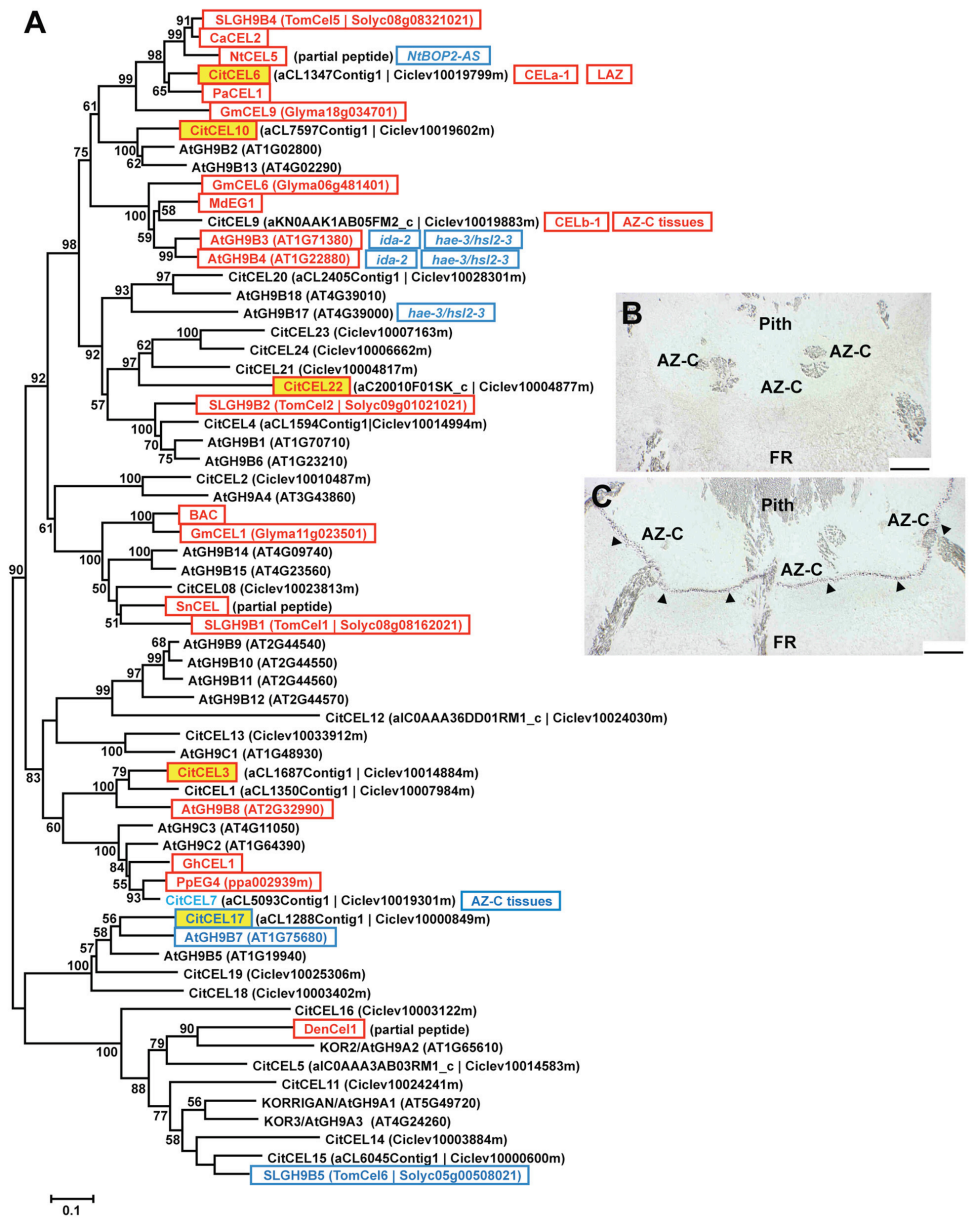
**Phylogenetic relationships between cellulases/endo-1,4-β-glucanases (CELs) and gene expression changes in response to ethylene inAZ-C and FR cells. (A)** CELs annotated in the *Citrus clementina* haploid genome (Wu et al., 2014), regulated by ethylene in AZ-C cells and/or FR cells of Washington Navel maturing fruits and previously described as related to the abscission process in other plant species are shown. Phylogenetic trees are based on multiple alignments of proteins using the profile alignment function of ClustalW (http://www.ch.embnet.org/software/ClustalW-XXL.html) and were generated with MEGA7 (Kumar et al., 2016) using the neighbor-joining algorithm with 1000 bootstrap replicates. Only bootstrap supports higher than 50% were considered and are shown in the nodes. Sequences are color-coded as follows: CitXXXXX, up-regulated exclusively in AZ-C cells; CitXXXXX, down-regulated exclusively in AZ-C cells; CitXXXXX, represented in the 20 K citrus microarray (Martinez-Godoy et al., 2008) but without hybridization results. ATXGXXXXX and ATXGXXXXX, up- and down-regulated, respectively, in Arabidopsis stamen-AZ cells (Lashbrook and Cai, 2008); XXXXX, and XXXXX, up- and down-regulated, respectively, during AZ activation in other plant species. CEL-a1 and CEL-b1, cellulases previously identified and characterized in citrus AZs (Burns et al., 1998). LAZ, up-regulated in the citrus laminar AZ (LAZ) cells during ethylene- and water stress-promoted leaf abscission (Agustí et al., 2008, 2012); AZ-C tissues, up-regulated in citrus AZ-C and surrounding tissues (Cheng et al., 2015). Transcripts of *NtCEL5* were down-regulated in the corolla base of tobacco plants over-expressing an antisense-oriented sequence of *NtBOP2* (NtBOP2-AS; Wu et al., 2012). Transcripts of *AtGH9B3* and *AtGH9B4* were down-regulated in receptacles of ida-2 mutant plants (Liu et al., 2013) and hae-2/hsl2-3 double mutant plants (Niederhuth et al., 2013) while transcripts of *AtGH9B17* were down-regulated only in receptacles of *hae-2/hsl2-3* double mutant plants (Niederhuth et al., 2013). **(B,C)** *CitCEL6* expression by *in situ* hybridization in the AZ-C of Ricalate Navel fruits after 24 h of ACC treatment (**B**, sense probe; **C**, anti-sense probe). Hybridization is indicated by the presence of a dark purple precipitate (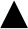). AZ-C, abscission zone C; FR, fruit rind. Scale bars: 500 μm.

Cellulases/endo-1,4-β-glucanases (CELs; EC 3.2.1.4) belong to the glycoside hydrolase family 9 (GH9; CAZy; http://www.cazy.org; Henrissat, 1991; Lombard et al., 2014) and are classified in Groups A, B and C based on their particular protein domains (Libertini et al., 2004; Urbanowicz et al., 2007). Figure 4A shows the phylogenetic analysis of the citrus and *Arabidopsis* GH9 families and the deduced proteins of this family previously related to the abscission process in other plant species. Interestingly, three CELs up-regulated exclusively in the AZ-C (*CitCEL6, CitCEL10*, and *CitCEL22*) fell in the same subclade with other CELs up-regulated during abscission in other plant species (Figure 4A, Figure S4). *CitCEL6* and *CitCEL10* were closely related to CELs induced during abscission in avocado (*PaCEL1*; Tonutti et al., 1995), tomato (*SLGH9B4*; Brummell et al., 1999), pepper (*CaCEL2*; Ferrarese et al., 1995), tobacco (*NtCEL5*; Wu et al., 2012), and soybean (*GmCEL09*; Tucker et al., 2007), while *CitCEL22* was more closely related to the tomato CEL *SLGH9B2* (Brummell et al., 1999). *CitCEL6* was previously reported as up-regulated in the citrus laminar abscission zone (LAZ) and the AZ-C (*CsCEL-a1;* Burns et al., 1998; Agustí et al., 2008, 2009, 2012). In addition, *in-situ* hybridizations on longitudinal AZ-C sections for *CitCEL6* showed specific signal in activated AZ-C cells (Figures 4B,C), supporting a potential role of this CEL in cell wall degradation during the abscission process. However, *CitCEL9* did not show a significant change in gene expression although it has been reported as up-regulated in both the LAZ and the fruit AZ-C (CsCEL-b1; Burns et al., 1998; Cheng et al., 2015). This gene showed a very close phylogenetic relationship to *AtGH9B3* and *AtGH9B4*, which were down-regulated in *ida-2* and *hae hsl2 Arabidopsis* mutant plants (Liu et al., 2013; Niederhuth et al., 2013). This could be due to the different methods used for AZ-C cells isolation and gene expression analyses. *CitCEL3* was also up-regulated exclusively in the AZ-C (Figure 4A). It was located in a different subclade, closely related to *AtGH9B8* that was up-regulated during stamen abscission in *Arabidopsis* (Lashbrook and Cai, 2008). On the other hand, *CitCEL17*, down-regulated exclusively in the AZ-C, was closely related to *AtGH9B7* that was also down-regulated during stamen abscission in *Arabidopsis* (Lashbrook and Cai, 2008).

##### Polygalacturonases

**Figure 5:**
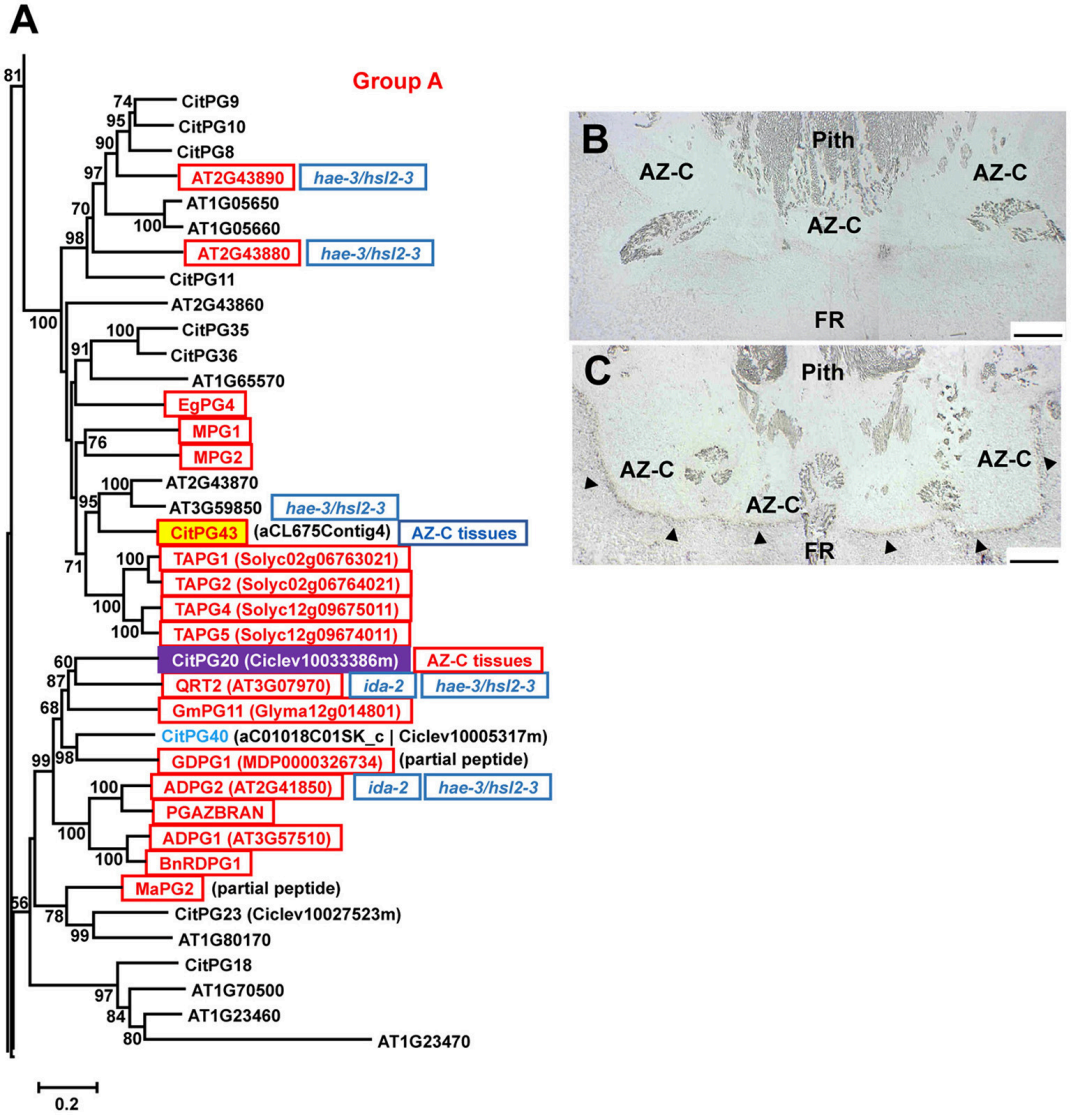
**Phylogenetic relationships between polygalacturonases (PGs) of the Group A (GH28) and gene expression changes in response to ethylene in AZ-C and FR cells**. PGs of the Group A located at the *Citrus clementina* haploid genome, regulated by ethylene in AZ-C cells and/or FR cells of Washington Navel maturing fruits and previously described as related to the abscission process in Arabidopsis (Lashbrook and Cai, 2008), tomato (Kalaitzis et al., 1995; Li et al., 2015; Hong and Tucker, 1998), apple (Atkinson et al., 2002; Li and Yuan, 2008), oilseed rape (Petersen et al., 1996; Sander et al., 2001; Gonzalez-Carranza et al., 2002), melon (Hadfield et al., 1998), oil palm (Roongsattham et al., 2012), and banana (Mbéguié-a-Mbéguié et al., 2009) are shown. Phylogenetic trees are based on multiple alignments of proteins using the profile alignment function of ClustalW (http://www.ch.embnet.org/software/ClustalW-XXL.html) and were generated with MEGA7 (Kumar et al., 2016) using the neighbor-joining algorithm with 1000 bootstrap replicates. Only bootstrap supports higher than 50% were considered and are shown in the nodes. Sequences are color-coded as follows: CitXXXXX, up-regulated exclusively in AZ-C cells; CitXXXXX, localization of transcripts in the citrus fruit AZ-C by *in situ* hybridization; CitXXXXX, represented in the 20 K citrus microarray (Martinez-Godoy et al., 2008) but without hybridization results. AZ-C tissues, up-regulated in citrus AZ-C and surrounding tissues (Cheng et al., 2015). ATXGXXXXX, up-regulated in Arabidopsis stamen-AZ cells (Lashbrook and Cai, 2008); XXXXX, up-regulated during AZ activation in other plant species. Transcripts of *ADPG2* (AT2G41850) and *QRT2* (AT3G07970) were down-regulated in receptacles of ida-2 mutant plants (Liu et al., 2013) and *hae-2/hsl2-3* double mutant plants (Niederhuth et al., 2013) while transcripts of *AT2G43880, AT2G43890* and *AT3G59850* were down-regulated only in receptacles of hae-2/hsl2-3 double mutant plants (Niederhuth et al., 2013). **(B,C)** *CitPG20* expression by *in situ* hybridization in the AZ-C of Ricalate Navel fruits after 24 h of ACC treatment (**B**, sense probe; **C**, anti-sense probe). Hybridization is indicated by the presence of a dark purple precipitate (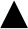). AZ-C, abscission zone C; FR, fruit rind. Scale bars: 500 μm.

**Figure 6:**
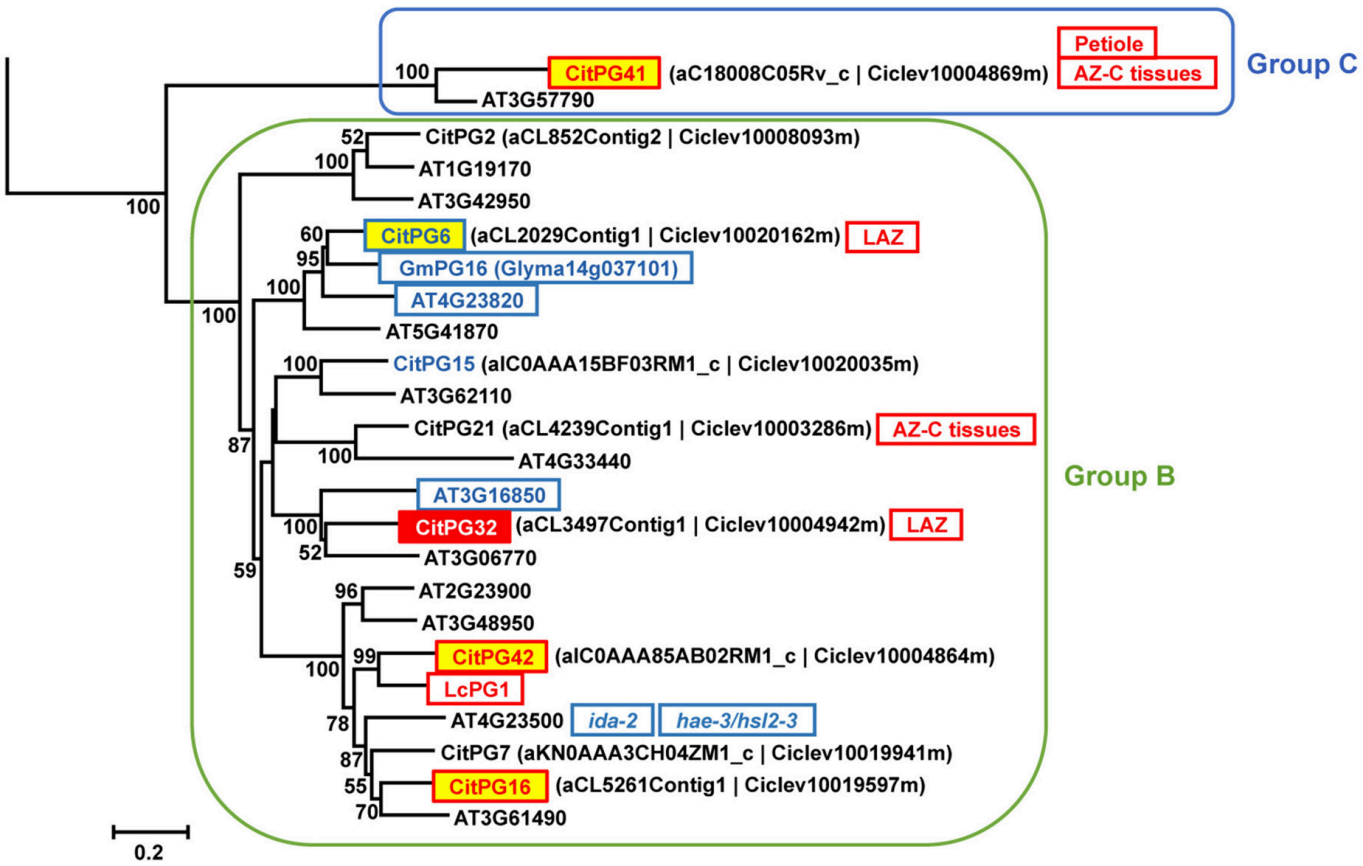
**Phylogenetic relationships between PGs from Groups B and C (GH28) and gene expression changes in response to ethylene in AZ-C and FR cells**. PGs of the Groups B and C located at the *Citrus clementina* haploid genome, regulated by ethylene in AZ-C cells and/or FR cells of Washington Navel maturing fruits and previously described as related to the abscission process in Arabidopsis (Lashbrook and Cai, 2008), soybean (Tucker et al., 2007), and lychee (Peng et al., 2013). Phylogenetic trees are based on multiple alignments of proteins using the profile alignment function of ClustalW (http://www.ch.embnet.org/software/ClustalW-XXL.html) and were generated with MEGA7 (Kumar et al., 2016) using the neighbor-joining algorithm with 1000 bootstrap replicates. Only bootstrap supports higher than 50% were considered and are shown in the nodes. Sequences are color-coded as follows: CitXXXXX, up-regulated exclusively in AZ-C cells; CitXXXXX, down-regulated by exclusively in AZ-C cells; CitXXXXX, up-regulated in both AZ-C and fruit rind (FR) cells; CitXXXXX, down-regulated exclusively in FR cells. ATXGXXXXX, down-regulated in Arabidopsis stamen-AZ cells (Lashbrook and Cai, 2008); XXXXX, up-regulated during AZ activation in other plant species. LAZ, preferentially expressed in the citrus laminar AZ (LAZ) cells during ethylene-promoted leaf abscission (Agustí et al., 2009); Petiole, preferentially expressed in the citrus leaf petiolar cells during ethylene-promoted leaf abscission (Agustí et al., 2009); AZ-C tissues, up-regulated in citrus AZ-C and surrounding tissues (Cheng et al., 2015). Transcripts of *AT4G23500* were down-regulated in receptacles of ida-2 mutant plants (Liu et al., 2013) and hae-2/hsl2-3 double mutant plants (Niederhuth et al., 2013).

Polygalacturonases (PGs; EC 3.2.1.15) belong to the glycoside hydrolase family 28 (GH28) and are classified in three different groups (Groups A, B, and C) based on phylogenetic analyses (Kim et al., 2006). The large PG gene family of citrus (Figures 5A, 6, Figure S4) was highly represented in the AZ-C transcriptome. Regarding Group A, to which most of the PGs belong, *CitPG43* was up-regulated exclusively in the AZ-C cells (Figure 5A) and *CitPG20* was detected specifically in the abscission-activated AZ-C cells by *in-situ* hybridization (Figures 5B,C). Another three PGs, *CitPG16*, and *CitPG42*, belonging to Group B, and the only PG belonging to Group C, *CitPG41*, were also up-regulated exclusively in AZ-C cells during ethylene-promoted fruit abscission (Figure 6). On the other hand, a PG belonging to Group B, *CitPG6*, was down-regulated exclusively in the AZ-C (Figure 6) although it has been reported to be predominantly expressed in the LAZ cells of citrus leaves during ethylene-promoted abscission (Agustí et al., 2009).

Many PGs were shown to be involved in organ abscission in different plant species. Moreover, several of the citrus PGs up-regulated in AZ-C cells during fruit abscission showed close phylogenetic relationship with some of the most actively expressed abscission-associated PGs. This was the case of *CitPG43* and *CitPG20* (Figure 5A). *CitPG43* was grouped in the same subclade as different abscission-related PGs from tomato (*TAPG1, TAPG2, TAPG4*, and *TAPG5*; Kalaitzis et al., 1995, 1997; Hong and Tucker, 1998), ripe melon (*MPG1* and *MPG2*; Hadfield et al., 1998), and oil palm fruit (*EgPG4*; Roongsattham et al., 2012). On the other hand, *CitPG20* was grouped in the same subclade as several PGs associated with abscission or dehiscence in *Arabidopsis* (*ADPG1, ADPG2*, and *QRT2*; Gonzalez-Carranza et al., 2002, 2007; Ogawa et al., 2009), rape (*PGAZBRAN* and *RDPG1*; Petersen et al., 1996; Sander et al., 2001; Gonzalez-Carranza et al., 2002), apple (*GDPG1*; Atkinson et al., 2002), soybean (*GmPG11*; Tucker et al., 2007), and banana (*MaPG2*; Mbéguié-a-Mbéguié et al., 2009). Both *CitPG20* and *CitPG43* have recently been reported to be up-regulated by ethephon in citrus fruit AZ-C-enriched tissues (Cheng et al., 2015). In relation to the Group B of PGs up-regulated exclusively in the AZ-C, *CitPG16* and *CitPG42*, that were closely related phylogenetically, were also closely related to PGs involved in lychee fruitlet abscission (*LcPG1*; Peng et al., 2013) and floral organ abscission (Liu et al., 2013; Niederhuth et al., 2013). Regarding *CitPG6*, it was grouped in the same subclade as *GmPG16* (Figure 6) that was down-regulated during abscission in soybean (*GmPG16*; Tucker et al., 2007). Therefore, despite PGs comprise a large gene family in plants, it has been possible to highlight a strong phylogenetic connection to PGs that are actively regulated during organ abscission.

##### Pectate lyases

Pectate lyases (PLs; EC 4.2.2.2) belong to pectate lyase family 1 (PL1; CAZy; http://www.cazy.org; Henrissat, 1991; Lombard et al., 2014) and are classified in five different groups (Groups I, II, III, IV, and V; Sun and Van Nocker, 2010). Group I of PLs contained all enzymes previously reported to be induced during organ abscission in *Arabidopsis* (*AtPLL18, AtPLL19, AtPLL22, AtPLL23, AtPLL24*, and *AtPLL25*; Lashbrook and Cai, 2008; Niederhuth et al., 2013), soybean (*GmPL01* and *GmPL02*; Tucker et al., 2007), banana (*MaPEL1*; Mbéguié-a-Mbéguié et al., 2009), and rose (*RbPEL1*; Singh et al., 2011; Figure S5). In agreement with that, the expression of *CitPL5* was up-regulated exclusively in the AZ-C during ethylene-promoted fruit abscission. *CitPL19* was up-regulated in both the AZ-C and the FR and was closely related to PLs that were also up-regulated in the AZ of rose (*RbPEL1*; Singh et al., 2011) and *Arabidopsis* (*AtPLL25* and *AtPLL26*; Lashbrook and Cai, 2008; Sun and Van Nocker, 2010). Furthermore, *CitPL19* was previously reported to be up-regulated exclusively in the citrus LAZ (Agustí et al., 2008, 2009), suggesting a role in fruit and leaf abscission but also in cell wall degradation events taking place in the FR. Three citrus PLs (*CitPL5, CitPL7*, and *CitPL19*) have recently been reported to be up-regulated by ethephon in fruit AZ-C-enriched tissues (Cheng et al., 2015; Figure S5). On the other hand, *CitPL1*, also belonging to Group I-PLs, was down-regulated exclusively in the AZ-C.

#### Other cell wall modifying enzymes

In addition to the gene families mentioned above, several cell wall modification-related families included genes regulated by ethylene in the AZ-C and/or the FR such as pectin-methylesterases (PMEs; family CE8), pectin-acetylesterases (PAEs; family CE13), β-galactosidases (β-GALs and members of the GH2 gene family), β-glucosidases (β-GLUs; family GH1), endo-β-mannosidases (β-MANs; family GH5), xyloglucan endotransglucosylases/hydrolases (XTHs; family GH16), β-xylosidases (β-XYLs; family GH3), α-xylosidases (α-XYLs; family GH31), and expansins (EXPs; Table 1, Tables S2, S4). Phylogenetic relationships between citrus, *Arabidopsis* and abscission-related genes from different species are shown in Figures S3–S6.

In summary, this study revealed a link between phylogenetic proximity and expression pattern between cell wall remodeling enzymes of citrus and those previously reported to be involved in organ abscission in other plant species, suggesting that different plant species use common genes to control similar processes. Therefore, those genes exclusively up-regulated in the AZ-C and closely related to genes that were also up-regulated during abscission in other plant species (*CitCEL3, CitCEL6, CitCEL10, CitCEL22, CitPG43, CitPG16, CitPG20, CitPG42, CitPL5, CitXTH24*, and *CitEXP14*) might have a molecular function during citrus fruit abscission (Table 1, Figures 4–6, Figures S3–S6). Furthermore, phylogenetic analysis also provided insights into the functional divergence between members of these gene families. More importantly, our results provided a set of genes belonging to groups, subfamilies, or families that have not yet been described as involved in abscission. These include, the Group 1 of PMEs and the family CE13 (Figure S3), the Group C of PGs (Figure 6), the family GH2 of β-GALs, the family GH1 (Figure S4), and the subfamily EXLB of EXPs (Figure S6).

### Pectic polysaccharides change their spatial distribution in the cell walls of AZ-C cells during abscission

**Figure 7:**
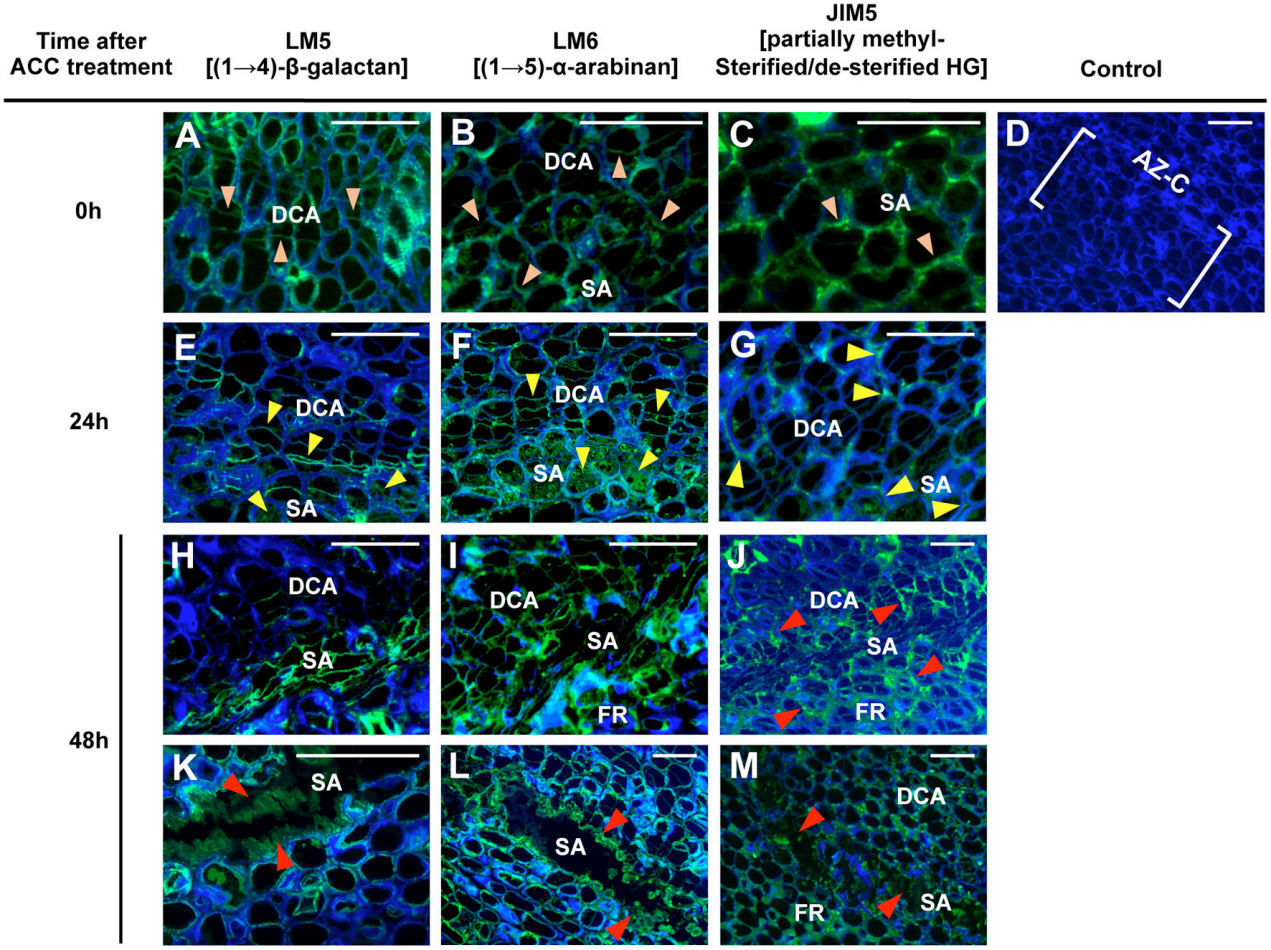
**Immunolocalization of pectic polysaccharides in the AZ-C**. Longitudinal sections of tissue containing the AZ-C of Ricalate Navel maturing fruits were incubated with the monoclonal antibodies (mAb) LM5 **(A,E,H,K)**, LM6 **(B,F,I,L)**, and JIM5 **(C,G,J,M)** to detect (1,4)-β-D-galactans, (1,5)-α-L-arabinans and partially methylesterified/de-esterified HGs, respectively, after 0 (A, B and C), 24 **(E,F,G)** and 48 h **(H–M)** of ACC treatment. Control did not show immunofluorescence **(D)**. Scale bars: 5 μm. Key labeling: walls at the two AZ-C cell areas (divided cells area [DCA] and starch-rich area [SA]) and at the fruit rind (FR) cell layers just below the SA showing fluorescence due to each of the mAbs after 0 (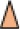), 24 (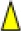) or 48 (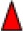) h of ACC treatment. Micrographs represent the merger of images from pectic epitopes detection by mAbs (green) and from cellulose detection by calcofluor white (blue).

Having identified a set of cell wall-related genes that were up-regulated during fruit abscission and to test whether such gene expression changes corresponded to enzymatic activity, we next attempted to identify alterations in pectic polysaccharides arrangements at the AZ-C cell walls. To examine such changes, we carried out an immunolocalization of pectic polysaccharides assay during ACC-promoted abscission. At 0 h, the epitopes (1 → 4)-β-D-galactan, (1 → 5)-α-L-arabinan, and partially methylesterified/de-esterified homogalacturonan (HG) recognized by the mAbs LM5 and LM6 and JIM5, respectively, were homogeneously distributed along cell walls of the AZ-C (Figures 7A–C). An increase in the (1 → 4)-β-D-galactan (LM5) labeling intensity in the starch-rich cell area of the AZ-C was observed at 24 h after ACC treatment (Figure 7E) whereas (1 → 5)-α-L-arabinan (LM6) and partially methylesterified/de-esterified HGs (JIM5) labeling highlighted cell wall changes in the divided cell area (Figures 7F,G). At a more advanced stage of the process (48 h), the labeling intensity detected for the three mAbs was higher at the cell layers closer to the separation line (Figures 7H–J), probably due to the accumulation of residual compounds of these pectic polysaccharides from the dissolution of the middle lamella and cell walls. Furthermore, an amorphous material with high fluorescence was observed at the regions where cell separation was completed (Figures 7K–M), in accordance with findings observed in Figure 2B and also with the first anatomical and histological description of citrus fruit abscission reported by Wilson and Hendershott (1968).

Changes in the pectic polysaccharides distribution in the AZ-C cell walls enabled us to correlate evidences of enzymatic activity with gene expression results. In particular, based on immunodetection of partially methylesterified/de-esterified HG and expression results, we propose that PMEs *CitPME24* and *CitPME41* and PAE *CitPAE4* may act on de-esterification of HGs in the AZ-C cell walls (Table 1, Figure S3). The activity of PMEs and PAEs is thought to facilitate the subsequent action of pectin hydrolases (Chen and Mart, 1996). Thus, the PGs *CitPG43, CitPG16, CitPG20, CitPG41*, and *CitPG42*, and the PLs *CitPL5*, and *CitPL19* may potentially hydrolyze the HGs highly accessible after *CitPME24, CitPME41*, and *CitPAE4* activity (Table 1, Figures 5, 6, Figures S3–S5). Finally, the only α-L-arabinofuranosidase identified in citrus (*CitASD1*) did not show significant changes in gene expression based on the statistical cutoff mentioned in materials and methods (Figure S4). In *Arabidopsis*, it has been reported that AtBXL1 and AtBXL3 acted as bifunctional α-L-arabinofuranosidase/β-D-xylosidases (Minic et al., 2004; Arsovski et al., 2009). Therefore, changes observed in (1, 5)-α-L-arabinans at the AZ-C might be due to the dual activity of β-XYLs such as *CitBXL11* and *CitBXL19*, which were in the same clade as *AtBXL1* and *AtBXL3* (Figure S4).

### A set of genes involved in lignin biosynthesis and polymerization are regulated in the AZ-C cells

**Figure 8:**
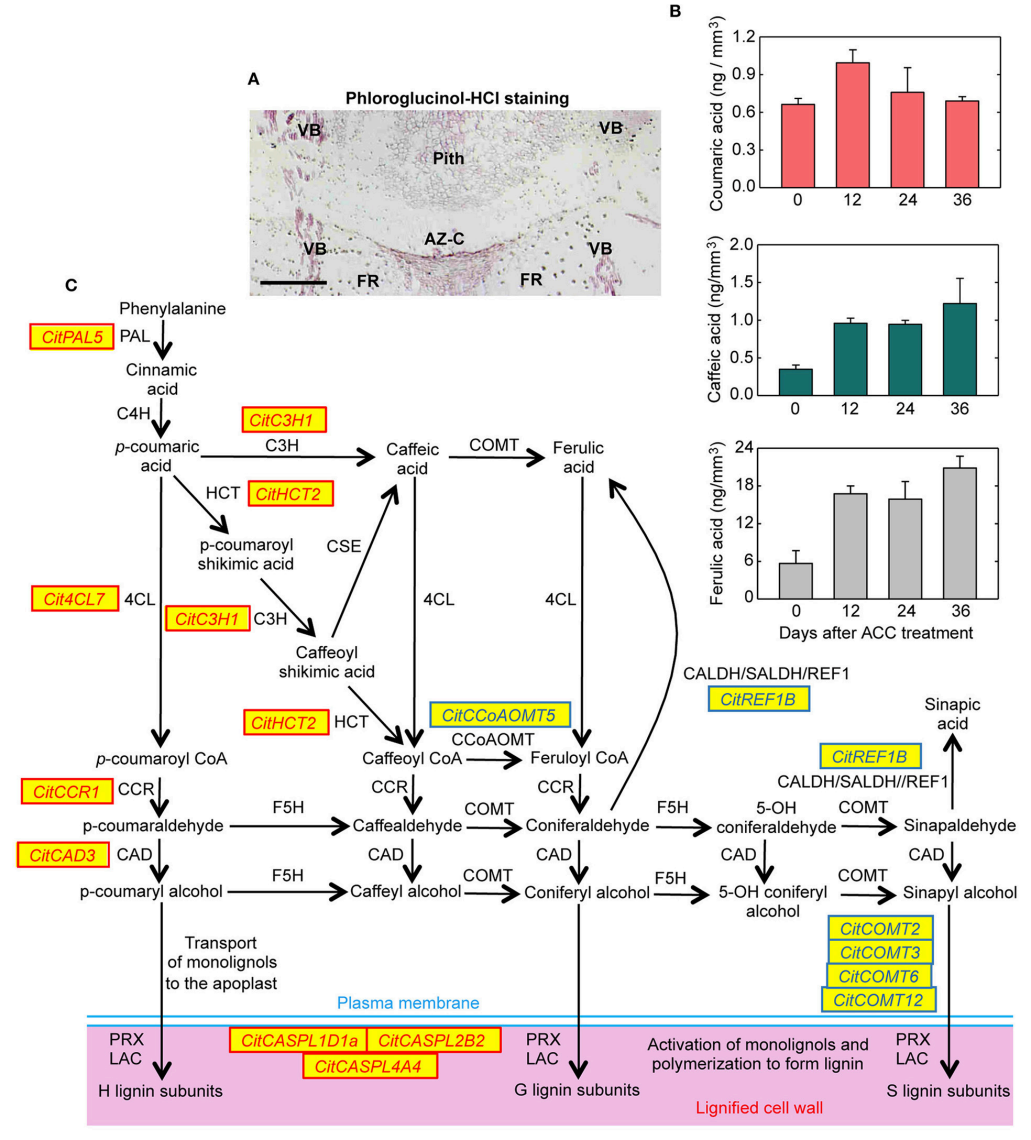
**Lignin biosynthesis and deposition in the abscission zone area during citrus fruit abscission. (A)** Tissue localization of lignin through phloroglucinol-HCl staining in longitudinal sections of the AZ-C from Washington Navel fruits treated for 48 h with ethylene. Lignin is deposited at the central core of the AZ-C, at the separation line, and spreads to the adjacent cells of the fruit rind through the distal side of the AZ-C. Scale bar: 500 μm. Key labeling: AZ-C, abscission zone C; FR, fruit rind; VB, vascular bundles. **(B)** Lignin biosynthesis intermediates were quantified through UPLC-MS/MS in AZ-C cells at 0, 12, 24, and 36 h after ACC treatment. Data are expressed as ng of coumaric acid, caffeic acid and ferulic acid per mm^3^ of microdissected tissue. The results are means of three independent samples containing ~40,000 pooled AZ-C cells ± SE. **(C)** Genes belonging to the general phenylpropanoid and monolignol biosynthesis pathways and lignin polymerization up- CitXXXXX or down-regulated CitXXXXX exclusively in the fruit AZ-C cells during ethylene-promoted citrus fruit abscission. Enzymes and proteins associated with monolignol biosynthesis and polymerization are: phenylalanine ammonia lyase (PAL), trans-cinnamate 4-hydroxylase (C4H), 4-coumarate:CoA ligase (4CL), hydroxycinnamoyl-CoA:shikimate/quinate hydroxycinnamoyl transferase (HCT), coniferaldehyde dehydrogenase/sinapaldehyde dehydrogenase (CALDH/SALDH), caffeoyl shikimate esterase (CSE), *p*-coumarate 3-hydroxylase (C3H), caffeoyl-CoA 3-O-methyltransferase (CCoAOMT), cinnamoyl-CoA reductase (CCR), ferulate 5-hydroxylase (F5H), caffeic acid O-methyltransferase (COMT), cinnamyl alcohol dehydrogenase (CAD), Casparian strip membrane domain protein-like (CASPL), laccase (LAC) and peroxidase (PRX).

Significant expressed genes belonging to different gene families involved the monolignol biosynthesis pathway were up-regulated by ethylene exclusively in the AZ-C (Table 2, Figure 8, Figure S7). These included a phenylalanine ammonia-lyase (*CitPAL5*), a *p*-coumarate 3-hydroxylase (*CitC3H1*), a hydroxycinnamoyl-CoA shikimate/quinate hydroxycinnamoyl transferase (*CitHCT2*), a 4-coumarate-CoA ligase-like protein (*Cit4CL7*), a cinnamoyl-CoA reductase-like protein (*CitCCR1*), and a cinnamyl alcohol dehydrogenase (*CitCAD3*). In addition, three genes encoding proteins involved in the oxidative coupling of monolignols and belonging to the CASPARIAN STRIP MEMBRANE DOMAIN PROTEIN family (*CitCASPL1D1a, CitCASPL2B2*, and *CitCASPL4A4*) were also up-regulated by ethylene exclusively in the AZ-C (Table 2, Figure 8, Figure S7). These results correlated with the observation of lignin deposition in the AZ-C after 24 and 48 h of ethylene/ACC treatment (Figures 1B,C) and the increase in the level of lignin intermediates detected by UPLC-MS/MS in AZ-C cells (Figure 8B). A significant increase of coumaric acid was observed at 12 h after ACC treatment. In addition, levels of caffeic acid and ferulic acid, compounds that are synthesized from coumaric acid, increased at 12 h and were maintained up to 36 h. Taken together, these data mainly reflected the activation of the H lignin pathway which results from the incorporation of *p*-hydroxyphenyl (H) units into the lignin polymer (Figure 8C). However, G lignin (lignin with guaiacyl units) biosynthesis would be also possible in activated AZ-C cells since an increase in *CitC3H1* and *CitHCT2* expression and caffeic and ferulic acids levels also occurred in AZ-C cells despite any member of the CCoAOMTs gene family were up-regulated (Figure 8). Regarding S lignin biosynthesis, four caffeic acid *O*-methyltransferases (*CitCOMT2, CitCOMT3, CitCOMT6*, and *CitCOMT12*) were down-regulated exclusively in AZ-C cells (Table 2, Figure 8C). *CitCOMT3* was closely related in sequence to *AtCOMT1* (Figure S7), a member of the *Arabidopsis* COMT gene family with 5-hydroxyconiferaldehyde *O*-methyltransferase activity that has been implicated directly in S lignin synthesis (Nakatsubo et al., 2008). These results suggest that the S lignin pathway might be inactive in AZ-C cells during fruit abscission. In woody dicotyledons, such as citrus, lignin is polymerized from mostly G and S lignin subunits (Sarkanen and Ludwig, 1971). However, lignin composition can differ among cell types (Nakashima et al., 2008; Ruel et al., 2009) and expression data suggested that cell walls of AZ-C cells might be mainly enriched in H lignin and probably also in G lignin subunits.

**Table 2:** **Relative gene expression values (AZ-Ct vs. AZ-C0) of genes involved in lignin biosynthesis exclusively regulated by ethylene in AZ-C cells**.

**Name**	**Contig/Singleton ID**	**Microarray probe**	**Putative Ath orthologue**	**Relative expression [log2 (AZ-Ct/AZ-C0)]**	
				**12 h**	**24 h**
**PHENYLALANINE AMMONIA-LYASES (PALs)**					
*CitPAL5*	aC02002A11SK_c	C02002A11	AT2G37040	2.33	1.22
**4-COUMARATE:CoA LIGASES (4CLs)**					
*Cit4CL7*	aC31504D07EF_c	C31504D07	AT4G19010	–	1.00
***p*****-COUMARATE 3-HYDROXYLASES (C3Hs)**					
*CitC3H1*	aC32104F09EF_c	C32104F09	AT2G40890	1.03	0.65
**HYDROXYCINNAMOYL CoA:SHIKIMATE/QUINATE HYDROXYCINNAMOYLTRANSFERASES (HCTs)**					
*CitHCT2*	aIC0AAA81CG08RM1_c	IC0AAA81CG08	AT5G48930	0.58	0.56
**CAFFEOYL-CoA 3-O-METHYLTRANSFERASES (CCoAOMTs)**					
*CitCCoAOMT5*	aCL18Contig10	C31100A08	AT4G34050	–	−0.78
**CINNAMOYL-CoA REDUCTASES (CCRs)**					
*CitCCR1*	aCL8119Contig1	C34205C03	AT2G23910	0.94	1.39
**CONIFERALDEHYDE/SINAPALDEHYDE DEHYDROGENASES (CALDH/SALDH//REF1)**					
*CitREF1B*	aCL1370Contig1	C01019E09	AT3G24503	–	−0.97
**CAFFEIC ACID O-METHYLTRANSFERASES (COMTs)**					
*CitCOMT2*	aCL4061Contig1	C03007H04	AT5G54160	−0.75	–
*CitCOMT3*	aCL3343Contig1	C05133H01	AT5G54160	–	−0.87
*CitCOMT6*	aCL4936Contig1	C02003G01	AT5G54160	−1.41	–
*CitCOMT12*	aC08039B05SK_c	C08039B05	AT5G54160	–	−0.56
**CINNAMYL ALCOHOL DEHYDROGENASES (CADs)**					
*CitCAD3*	aCL33Contig1	C18001A01	AT4G37970	0.90	–
**CASPARIAN STRIP MEMBRANE DOMAIN PROTEINS (CASPs)**					
*CitCASPL1D1a*	aCL6688Contig1	C04010H11	AT4G15610	0.74	–
*CitCASPL2B2*	aCL5933Contig1	C32104D12	AT2G35760	0.82	–
*CitCASPL4A4*	aC08028B10SK_c	C08028B10	AT4G11655	0.93	0.61

*–, No significant regulation. Putative gene identifications are based on sequence homology with Arabidopsis thaliana. Additional data are shown in Figure S7*.

The role of lignin deposition has been associated with the generation of protective layers at the tissues remaining in the plant during the last step of the abscission process (Addicot, 1982; Agustí et al., 2008; Van Nocker, 2009). However, it has been suggested that lignification could also facilitate the mechanical cell wall breakage during cell separation processes (Sexton, 1979; Liljegren et al., 2000). In the AZ-C, lignin deposition only occurred at the distal side of the AZ-C (Figure 8A). This differential deposition of lignin strongly suggests that this polymer mainly acts by generating a tension in the fracture plane to facilitate cell wall breakage during citrus fruit abscission rather than forming protective layers.

## Conclusion

**Figure 9:**
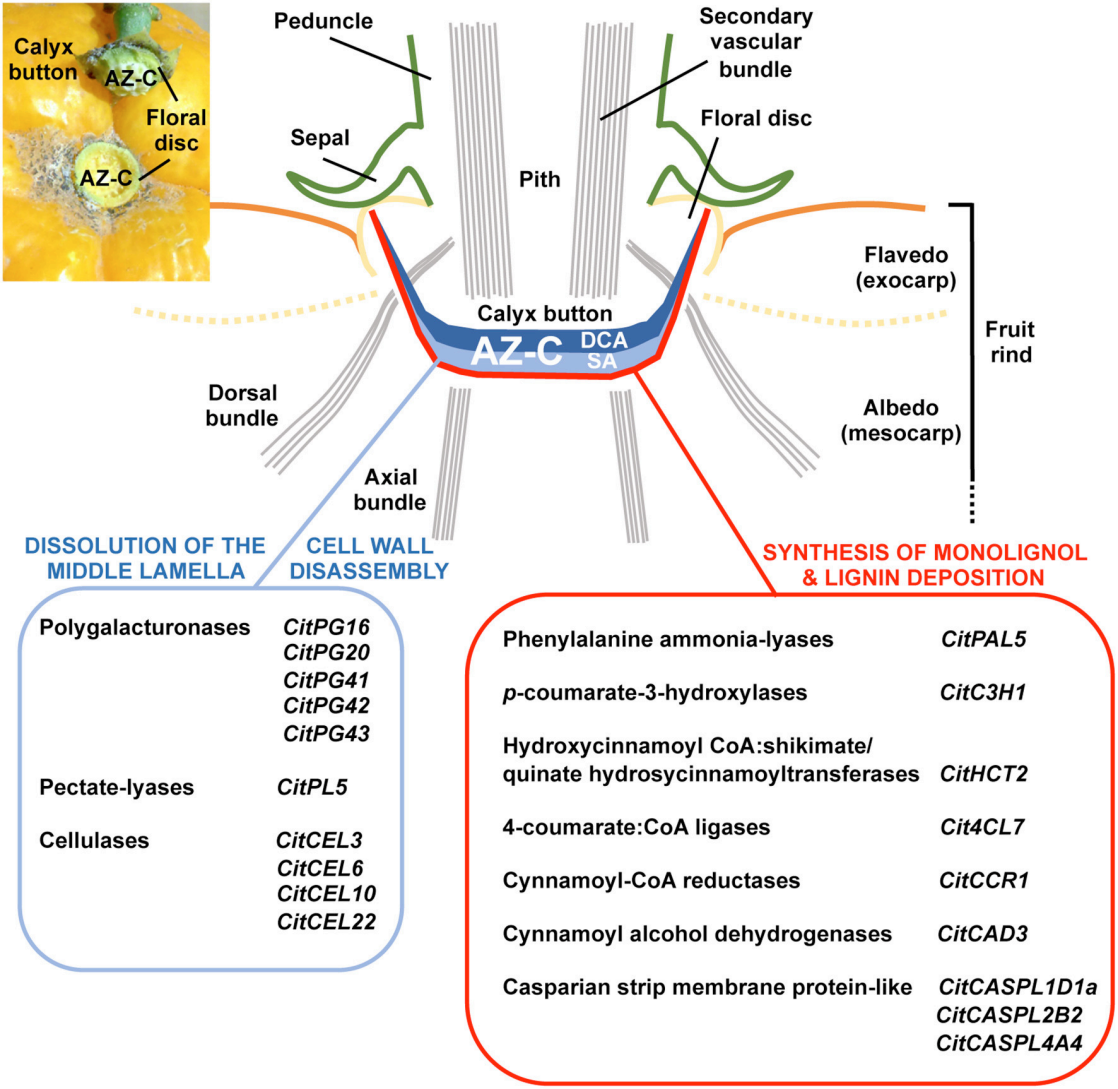
**Specific cellular and molecular events involved in the dissolution of the middle lamella, the disassembly of cell walls and the synthesis and deposition of lignin in the AZ-C during ethylene-promoted abscission**. The AZ-C consists of two different cell areas, the Divided Cells Area (DCA) and the Starch-rich Area (SA). The early cellular and molecular events associated with citrus fruit abscission occur in the central core of the AZ-C between the axial vascular bundles and spread up to the calyx button periphery reaching then the floral disc. The final outcome of this cell separation process is the shedding of the fruit remaining the calyx button attached to the tree as shown in the inset of the upper-left corner of the figure. Two parallel cellular events involving cell wall dissolution and synthesis and deposition of lignin occurred specifically in the SA of the AZ-C cells during abscission. These cellular events are potentially promoted by the tissue-specific expression of particular members of several gene families that have been clearly involved in those metabolic pathways.

In this work, the isolation of specialized cell types through LM, combined with the global transcriptional analysis of ethylene-promoted AZ-C cells and the comparison with adjacent FR cells, enabled us to identify an AZ-C-exclusive gene set potentially involved in citrus fruit abscission. This set of genes includes those related to cell wall remodeling as well as lignin biosynthesis and polymerization. The combined function of these genes would enable cell wall modifications necessary for organ detachment (Figure 9). These results, together with the anatomical and morphological analysis of the AZ-C, the determination of changes in pectic polysaccharides distribution and the deposition of extracellular polymers observed in the activated AZ-C, lead to the most comprehensive characterization of citrus fruit abscission performed to date. In addition, our work shows the first classification in citrus of gene families involved in cell wall modification, which are crucial in abscission, and reveals a robust nexus between phylogenetic proximity and expression pattern during abscission in citrus and other plant species, not previously described. Therefore, this study strongly suggests that different plant species use common genes to control the abscission process. The dataset provided in this study is a highly valuable resource for guiding future functional analyses in order to answer specific abscission-related questions. In particular, those cell wall-related genes, which are evolutionarily conserved in citrus and other plant species with similar expression pattern during abscission, would represent major candidate genes for further biotechnological approaches.

## Author contributions

PM, JA, MT, and FT conceived the survey and designed the experiments; PM performed laser microdissection of citrus tissues; VA and AG performed metabolite profiling analyses; PM, JA, and CD performed microarray experiments and analyses; MC, SC, and FT performed immunolocalization experiments; LE, MG, MP, and FT performed *in situ* hybridization experiments; PM, JA, MT, and FT wrote the article with contributions of all the authors; all authors discussed the results and edited the article.

### Conflict of interest statement

The authors declare that the research was conducted in the absence of any commercial or financial relationships that could be construed as a potential conflict of interest.

## Supplementary material

Table S1 **Specific primers used for sqRT-PCR**.

Table S2 **Genes regulated by ethylene exclusively in AZ-C or FR cells, and in both AZ-C and FR cells**.

Table S3 **Functional categories related to polysaccharide metabolism and subcellular localization regulated in the AZ-C and the FR**.

Table S4 **Identification of citrus genes belonging to different families of cell wall remodeling enzymes, and monolignol biosynthesis and polymerization**.

Figure S1 **sqRT-PCR-based relative expression in the AZ-C and the FR**.

Figure S2 **LM isolation of AZ-C and FR cells**.

Figure S3 **Phylogenetic relationships between Carbohydrate esterases**.

Figure S4 **Phylogenetic relationships between Glycoside hydrolases**.

Figure S5 **Phylogenetic relationships between Pectate lyases**.

Figure S6 **Phylogenetic relationships between Expansins**.

Figure S7 **Phylogenetic relationships between members of gene families associated with monolignol biosynthesis and polymerization**.
