# Corrigendum: Cell Wall Remodeling in Abscission Zone Cells during Ethylene-Promoted Fruit Abscission in Citrus

**DOI:** 10.3389/fpls.2017.00301

**Published:** 2017-03-07

**Authors:** Paz Merelo, Javier Agustí, Vicent Arbona, Mário L. Costa, Leandro H. Estornell, Aurelio Gómez-Cadenas, Silvia Coimbra, María D. Gómez, Miguel A. Pérez-Amador, Concha Domingo, Manuel Talón, Francisco R. Tadeo

**Affiliations:** ^1^Centre de Genòmica, Institut Valencià d'Investigacions AgràriesMontcada (València), Spain; ^2^Departamento de Biologia, Faculdade de Ciências, Universidade do PortoPorto, Portugal; ^3^Departament de Ciències Agràries i del Medi Natural, Universitat Jaume ICastelló de la Plana, Spain; ^4^Departamento de Desarrollo y Acción Hormonal en Plantas, Instituto de Biología Molecular y Celular de Plantas, Universidad Politécnica de Valencia-Consejo Superior de Investigaciones CientíficasValencia, Spain

**Keywords:** calyx abscission zone, cell wall modification, citrus fruit abscission, ethylene, lignin biosynthesis, phylogeny, transcriptomics

Corrigendum on Authors affiliation

In the original article, there was an error in Affiliation 1. Instead of “Centre de Genòmica, Institut Valencià d' Agràries, València, Spain”, it should be “Centre de Genòmica, Institut Valencià d'Investigacions Agràries, Montcada (València), Spain.”

In addition there was a mistake in the X-axis label of Figure 8B. Instead of “Days after ACC treatment,” it should be “Hours after ACC treatment.” The correct version of Figure 8 appears below. The authors apologize for these errors and state that they do not change the scientific conclusions of the article in any way.

**Figure 8:**
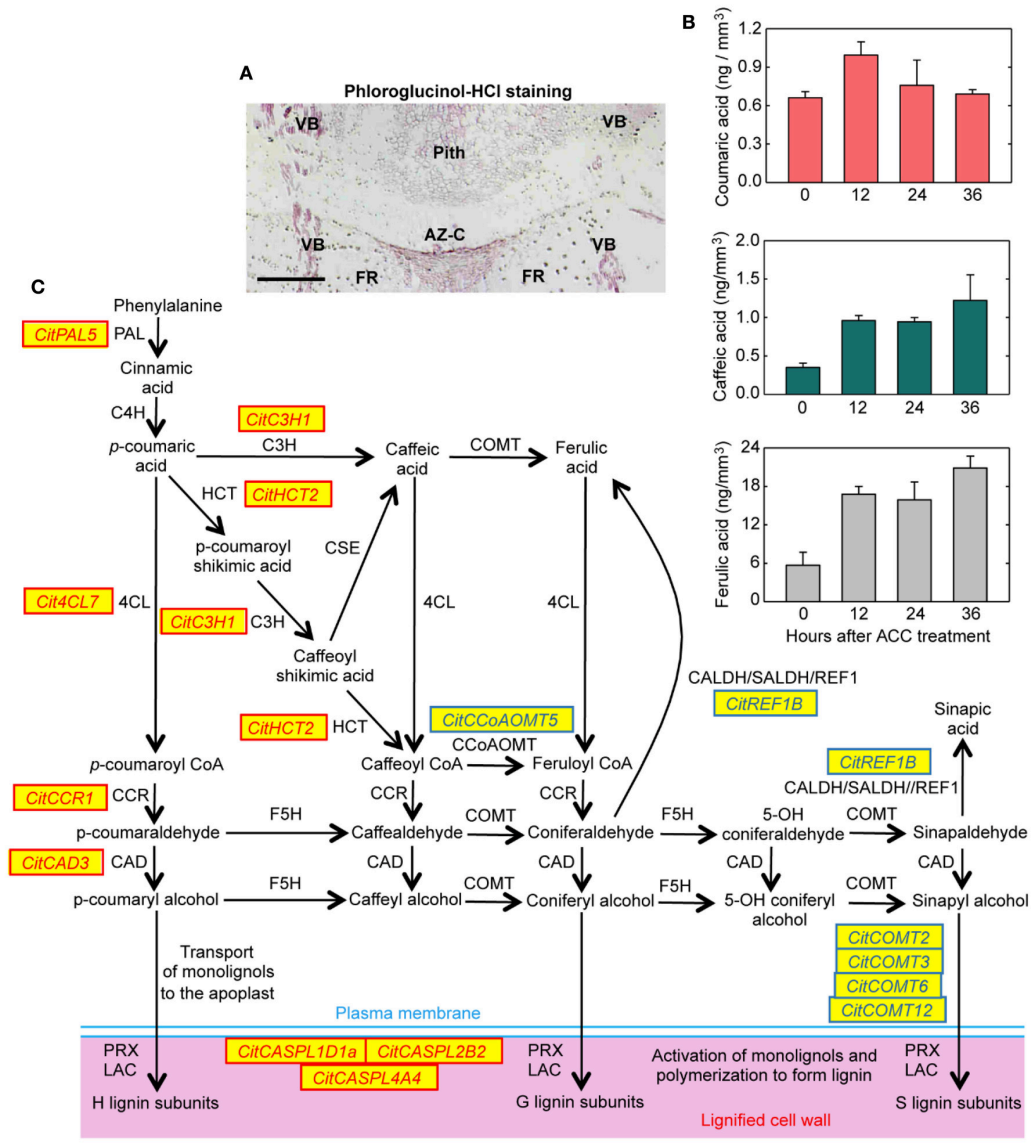
**Lignin biosynthesis and deposition in the abscission zone area during citrus fruit abscission**. **(A)** Tissue localization of lignin through phloroglucinol-HCl staining in longitudinal sections of the AZ-C from Washington Navel fruits treated for 48 h with ethylene. Lignin is deposited at the central core of the AZ-C, at the separation line, and spreads to the adjacent cells of the fruit rind through the distal side of the AZ-C. Scale bar: 500 μm. Key labeling: AZ-C, abscission zone C; FR, fruit rind; VB, vascular bundles. **(B)** Lignin biosynthesis intermediates were quantified through UPLC-MS/MS in AZ-C cells at 0, 12, 24, and 36 h after ACC treatment. Data are expressed as ng of coumaric acid, caffeic acid and ferulic acid per mm3 of microdissected tissue. The results are means of three independent samples containing ~40,000 pooled AZ-C cells ±SE. **(C)** Genes belonging to the general phenylpropanoid and monolignol biosynthesis pathways and lignin polymerization up- or down-regulated exclusively in the fruit AZ-C cells during ethylene-promoted citrus fruit abscission. Enzymes and proteins associated with monolignol biosynthesis and polymerization are: phenylalanine ammonia lyase (PAL), trans-cinnamate 4-hydroxylase (C4H), 4-coumarate:CoA ligase (4CL), hydroxycinnamoyl-CoA:shikimate/quinate hydroxycinnamoyl transferase (HCT), coniferaldehyde dehydrogenase/sinapaldehyde dehydrogenase (CALDH/SALDH), caffeoyl shikimate esterase (CSE), p-coumarate 3-hydroxylase (C3H), caffeoyl-CoA 3-O-methyltransferase (CCoAOMT), cinnamoyl-CoA reductase (CCR), ferulate 5-hydroxylase (F5H), caffeic acid O-methyltransferase (COMT), cinnamyl alcohol dehydrogenase (CAD), Casparian strip membrane domain protein-like (CASPL), laccase (LAC) and peroxidase (PRX).

## Conflict of interest statement

The authors declare that the research was conducted in the absence of any commercial or financial relationships that could be construed as a potential conflict of interest.

