# Supplementary material for: Cell Wall Remodeling in Abscission Zone Cells during Ethylene-Promoted Fruit Abscission in Citrus
Source: Front Plant Sci. 2017 Feb 8;8:126. doi: 10.3389/fpls.2017.00126 (PMC5296326; doi:10.3389/fpls.2017.00126)
Supplement: Table S2 — Genes regulated by ethylene exclusively in AZ-C or FR cells, and in both AZ-C and FR cells. [file Table2.PDF]

Table S2. Functional categories related to polysaccharide metabolism and subcellular localization regulated in the AZ-C and the FR. Percentage and number of unigenes for those categories with relevant differences between the AZ-C and the FR are shown. Percentage is based on the total number of significant unigenes from the AZ-C or the FR assigned to each functional category.

MIPS functional category	Abscission zone C			Fruit rind		
	% Unigenes	# Unigenes	p-value	% Unigenes	# Unigenes	p-value
**Metabolism**						
C-compound and carbohydrate metabolism						
Sugar, glucoside, polyol and carboxylate metabolism	2.47	106	2.8E-11	1.95	62	2E-04
Polysaccharide metabolism	0.68	29	3E-04	0.35	11	0.4
**Subcellular localization**						
Cell wall	0.77	33	0.1	0.53	17	0.3
Extracellular/secretion proteins	0.19	8	0.2	0.13	4	1
