# Supplementary material for: Cell Wall Remodeling in Abscission Zone Cells during Ethylene-Promoted Fruit Abscission in Citrus
Source: Front Plant Sci. 2017 Feb 8;8:126. doi: 10.3389/fpls.2017.00126 (PMC5296326; doi:10.3389/fpls.2017.00126)
Supplement: Table S4 — Identification of citrus genes belonging to different families of cell wall remodeling enzymes, and monolignol biosynthesis and polymerization. [file Table4.PDF]

Table S4. Specific primers used for sqRT-PCR.

Gene (microarray probe ID)	Forward and reverse primers (5' - 3')
CitCEL6 (C21007H10)	GGGGTTCATCTCTACCGTCCA
	GCTGATAATTATTACGATTATCG
CitPG43 (IC0AAA67DG09)	AACTGCCGAAAGAACTCAGG
	CTCCGAATTCCTCCAGTGAT
CitBMAN1 (C03004E07)	TCATCACAGCCAAAGGAGTG
	GGCTGTCACCACCATCACTA
CitEXP14 (IC0AAA14BD04)	TGTGCGAAGAAAGGAGGAAT
	CACTGGCTGTCACTTGGAAA
CitPL5 (IC0AAA15AF11)	AATGGCAATCCCTTCTCCTT
	TGGAGCCACATAATTGGTCA
CitXTH16 (C02023G10)	TCATCAGACGGTCCAAATCA
	CTGGTTCCAAAGGAGGGAAT
CitPME13 (C01011H09)	AATCAGAACACGGGCATCTC
	TCCAGTGCAAAGTCTCCATTC
CitAGP11 (IC0AAA56BH05)	TTCACCCACATTAGCACCAA
	AAATTGGTCAGAGCCACGAC
CitPAP1 (C02014A07)	TTTATGTCCCCGCTGAGAAC
	CCTTCCTGCCCATACCAATA
CitHD-Zip1 (C31809D05)	AAACATGCACAGAGCACTCG
	GAGAATATTCGCCCCCATCT
CitUBC1 (C06015C05)	TGGACGCTTCAGTCTGTTTG
	TCGTCAATCACCCCTTCTTT
