# Supplementary material for: Cell Wall Remodeling in Abscission Zone Cells during Ethylene-Promoted Fruit Abscission in Citrus
Source: Front Plant Sci. 2017 Feb 8;8:126. doi: 10.3389/fpls.2017.00126 (PMC5296326; doi:10.3389/fpls.2017.00126)
Supplement: Figure S1 — sqRT-PCR-based relative expression in the AZ-C and the FR. [file Image1.PDF]

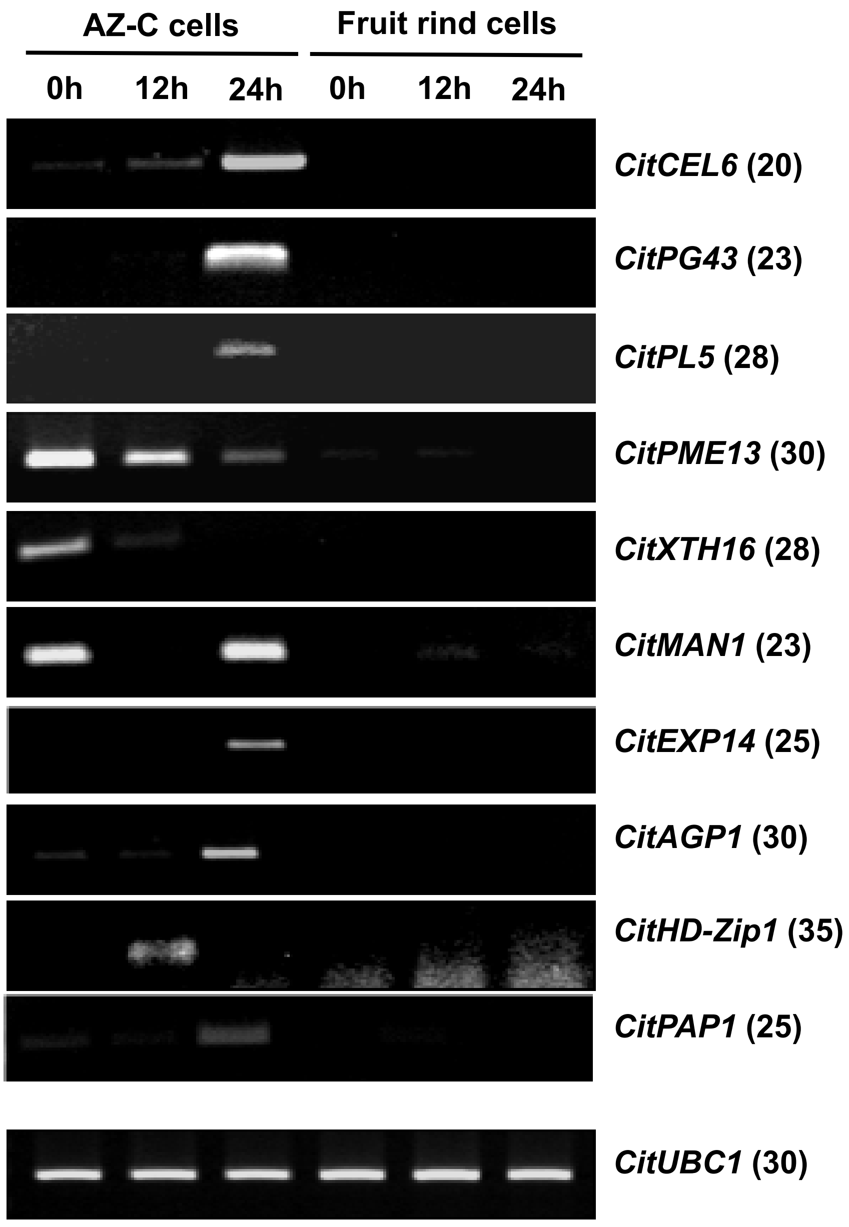

Figure S1. sqRT-PCR-based relative expression in the AZ-C and the FR. Numbers on the right hand side represent the cycles performed during the PCR.
