# Supplementary figures and images for: Cell Wall Remodeling in Abscission Zone Cells during Ethylene-Promoted Fruit Abscission in Citrus
Source: Front Plant Sci. 2017 Feb 8;8:126. doi: 10.3389/fpls.2017.00126 (PMC5296326; doi:10.3389/fpls.2017.00126)

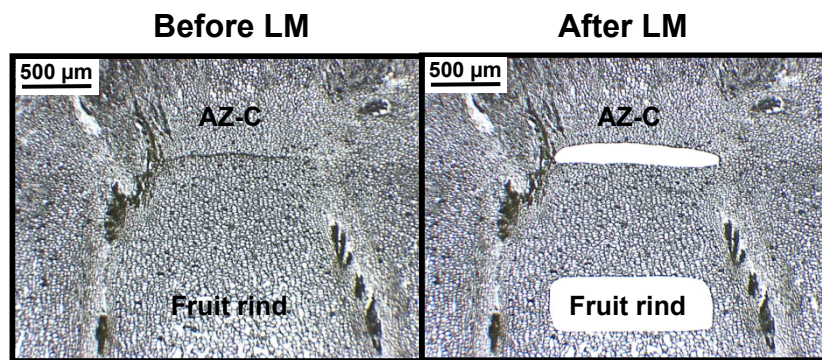

**Figure S2. LM isolation of AZ-C and FR cells.**

Supplement: Figure S2 — LM isolation of AZ-C and FR cells. [file Image2.PDF]
