# Supplementary material for: Cell Wall Remodeling in Abscission Zone Cells during Ethylene-Promoted Fruit Abscission in Citrus
Source: Front Plant Sci. 2017 Feb 8;8:126. doi: 10.3389/fpls.2017.00126 (PMC5296326; doi:10.3389/fpls.2017.00126)
Supplement: Figure S3 — Phylogenetic relationships between Carbohydrate esterases. [file Image3.PDF]

Figure S3. Phylogenetic relationships between carbohydrate esterases (CEs): pectin-methylesterases (CE8s) and pectin-acetylesterases (CE13s).

Color-coding of proteins regulated by ethylene in AZ-C cells and/or FR cells of Washington Navel maturing fruits or regulated during abscission in AZs in other plant species

CitXXXXX	Up-regulated exclusivelly in AZ-C cells
CitXXXXX	Up-regulated exclusivelly in fruit rind cells
CitXXXXX	Up-regulated in both AZ-C and fruit rind cells
CitXXXXX	Down-regulated exclusivelly in AZ-C cells
CitXXXXX	Down-regulated exclusivelly in fruit rind cells
CitXXXXX	Down-regulated in both AZ-C and fruit rind cells
CitXXXXX	Probe printed in the 20 K citrus microarray (Martínez-Godoy et al, 2008) but without hybridization results
XXXXX	Up-regulated during AZ activation in other plant species
XXXXX	Down-regulated during AZ activation in other plant species
XXXXX	β-glucuronidase (GUS) activity in floral organ AZ cells of *Arabidopsis thaliana*
LAZ	Up-regulated in LAZ-enriched tissues (Agustí et al., 2008; 2012) or preferentially expressed in LAZ cells (Agustí et al., 2009) during ethylene-promoted abscission in citrus leaves
Petiole	Up-regulated in petioles (Agustí et al., 2008) or preferentially expressed in petiolar cortical cells (Agustí et al., 2009) during ethylene-promoted abscission in citrus leaves
AZ-C tissues	Up-regulated in AZ-enriched tissues during ethylene-promoted abscission in orange fruits (Cheng et al, 2015)
AZ-C tissues	Down-regulated in AZ-enriched tissues during ethylene-promoted abscission in orange fruits (Cheng et al, 2015)
ida-2	Down-regulated in receptacles of *ida-2* plants (Liu et al, 2013)
hae-3/hsl2-3	Down-regulated in receptacles of *hae-2/hsl2-3* double mutant plants (Niederhuth et al, 2013)

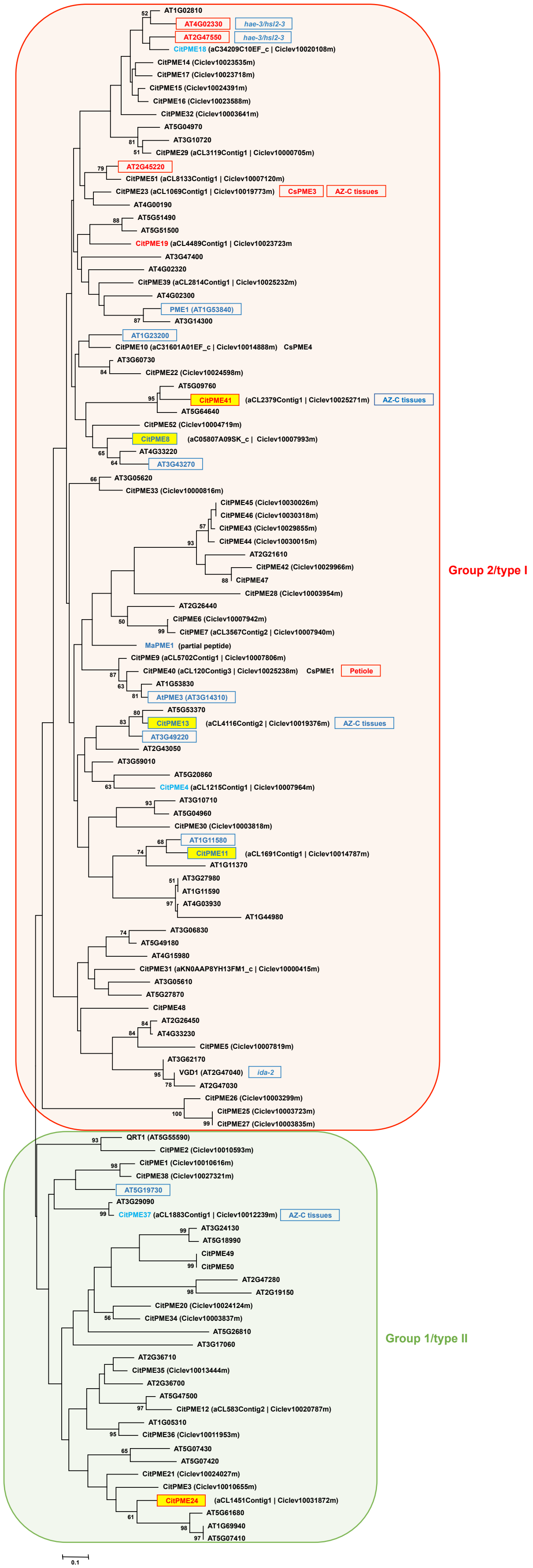

Figure S3A. Phylogenetic relationships between carbohydrate esterases (CEs): pectin-methylesterases (PMEs, CE8s). The phylogenetic tree shows the degree of similarity between the PMEs annotated in the genome of *Arabidopsis thaliana* (TAIR) and in the *Citrus clementina* haploid genome (Wu et al., 2014; Table S3) and those previously described as related to the abscission process in other plant species. Phylogenetic trees are based on multiple alignments of proteins using the profile alignment function of ClustalW (http://www.ch.embnet.org/software/ClustalW-XXL.html) and were generated with MEGA7 (Kumar et al., 2016) using the neighbor-joining algorithm with 1,000 bootstrap replicates. Only bootstrap supports higher than 50% were considered and are shown in the nodes. Accession numbers for the sequences of PME proteins regulated during organ abscission in different plant species are shown. *Arabidopsis thaliana* (Lashbrook and Cai, 2008): AT1G11580, AT1G23200, AT2G45220, AT2G47550, AT3G14310 (AtPME3), AT3G43270, AT3G49220, AT4G02330 and AT5G19730. Banana [*Musa acuminata*] (Mbéguié-A-Mbéguié et al., 2009): MaPME1 (ACQ85264). Orange [*Citrus sinensis*] (Nairn et al., 1998): CsPME3 (U82997).

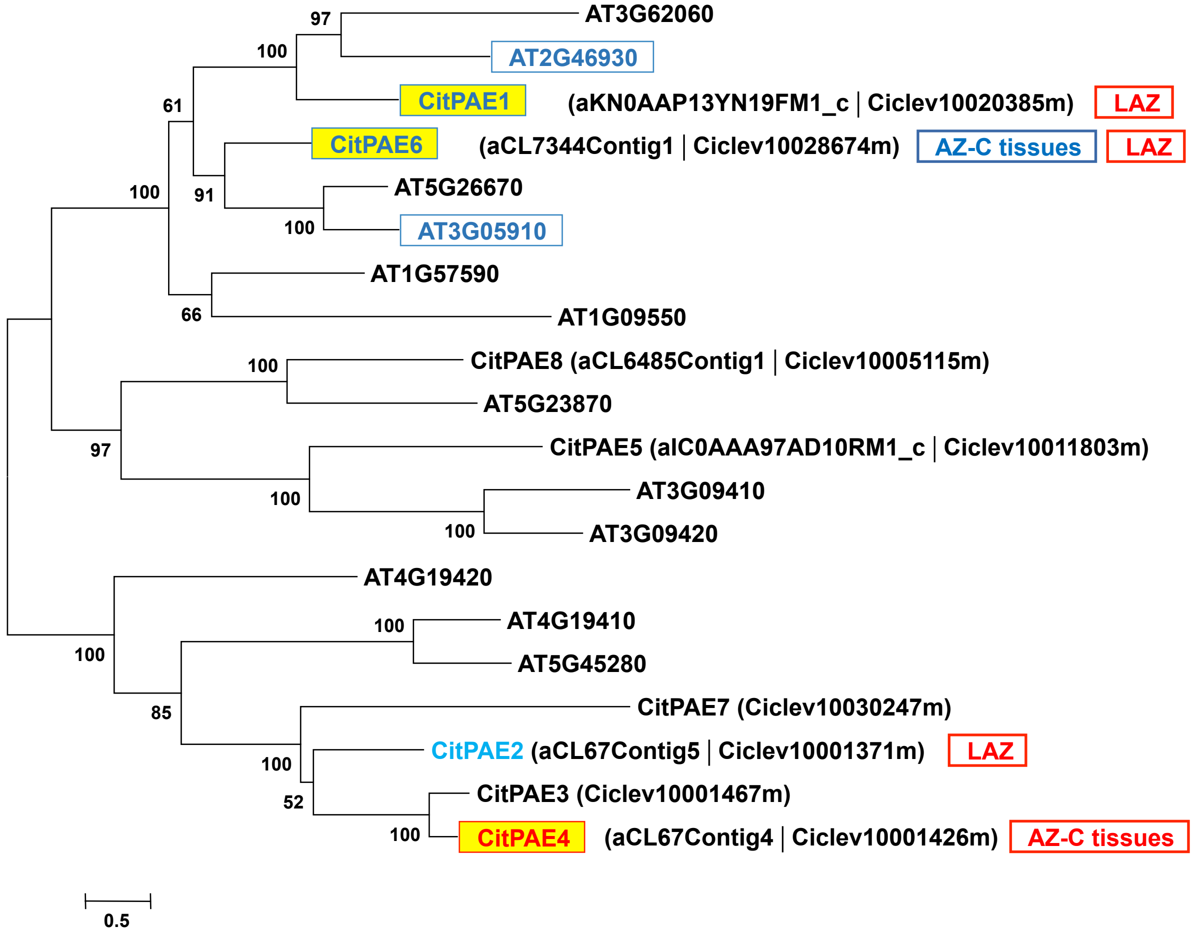

Figure S3B. Phylogenetic relationships between carbohydrate esterases (CEs): pectin-acethylesterases (PAEs, CE13s). The phylogenetic tree shows the degree of similarity between the PAEs annotated in the genome of *Arabidopsis thaliana* (TAIR) and in the *Citrus clementina* haploid genome (Wu et al., 2014; Table S3) and those previously described as related to the abscission process in other plant species. Phylogenetic trees are based on multiple alignments of proteins using the profile alignment function of ClustalW (http://www.ch.embnet.org/software/ClustalW-XXL.html) and were generated with MEGA7 (Kumar et al., 2016) using the neighbor-joining algorithm with 1,000 bootstrap replicates. Only bootstrap supports higher than 50% were considered and are shown in the nodes. Accession numbers for the sequences of PAE proteins regulated during organ abscission in *Arabidopsis thaliana* (Lashbrook and Cai, 2008): AT2G46930 and AT3G05910.

REFERENCES

Agustí, J., Gimeno, J., Merelo, P., Serrano, R., Cercos, M., Conesa, A., Talon, M., and Tadeo, F.R. (2012). Early gene expression events in the laminar abscission zone of abscission-promoted citrus leaves after a cycle of water stress/rehydration: involvement of CitbHLH1. *J Exp Bot* 63, 6079-6091.

Agustí, J., Merelo, P., Cercos, M., Tadeo, F.R., and Talon, M. (2008). Ethylene-induced differential gene expression during abscission of citrus leaves. *J Exp Bot* 59, 2717-2733.

Agustí, J., Merelo, P., Cercos, M., Tadeo, F.R., and Talon, M. (2009). Comparative transcriptional survey between laser-microdissected cells from laminar abscission zone and petiolar cortical tissue during ethylene-promoted abscission in citrus leaves. *BMC Plant Biol* 9, 127.

Cheng, C., Zhang, L., Yang, X., and Zhong, G. (2015). Profiling gene expression in citrus fruit calyx abscission zone (AZ-C) treated with ethylene. *Mol Genet Genomics* 290, 1991-2006.

Kumar, S., Stecher, G., and Tamura, K. (2016). MEGA7: Molecular Evolutionary Genetics Analysis version 7.0 for bigger datasets. *Molecular Biology and Evolution*.

Lashbrook, C.C., and Cai, S. (2008). Cell wall remodeling in Arabidopsis stamen abscission zones: Temporal aspects of control inferred from transcriptional profiling. *Plant Signaling & Behavior* 3, 733-736.

Liu, B., Butenko, M.A., Shi, C.L., Bolivar, J.L., Winge, P., Stenvik, G.E., Vie, A.K., Leslie, M.E., Brembu, T., Kristiansen, W., Bones, A.M., Patterson, S.E., Liljegren, S.J., and Aalen, R.B. (2013). NEVERSHED and INFLORESCENCE DEFICIENT IN ABSCISSION are differentially required for cell expansion and cell separation during floral organ abscission in Arabidopsis thaliana. *J Exp Bot* 64, 5345-5357.

Martinez-Godoy, M.A., Mauri, N., Juarez, J., Marques, M.C., Santiago, J., Forment, J., and Gadea, J. (2008). A genome-wide 20 K citrus microarray for gene expression analysis. *BMC Genomics* 9, 318.

Mbéguié-a-Mbéguié, D., Hubert, O., Baurens, F.C., Matsumoto, T., Chillet, M., Fils-Lycaon, B., and Sidibé-Bocs, S. (2009). Expression patterns of cell wall-modifying genes from banana during fruit ripening and in relationship with finger drop. *Journal of Experimental Botany* 60, 2021-2034.

Nairn, C.J., Lewandowski, D.J., and Burns, J.K. (1998). Genetics and expression of two pectinesterase genes in Valencia orange. *Physiologia Plantarum* 102, 226-235.

Niederhuth, C.E., Patharkar, O.R., and Walker, J.C. (2013). Transcriptional profiling of the Arabidopsis abscission mutant hae hsl2 by RNA-Seq. *BMC Genomics* 14, 37.

Wu, G.A., Prochnik, S., Jenkins, J., Salse, J., Hellsten, U., Murat, F., Perrier, X., Ruiz, M., Scalabrin, S., Terol, J., Takita, M.A., Labadie, K., Poulain, J., Couloux, A., Jabbari, K., Cattonaro, F., Del Fabbro, C., Pinosio, S., Zuccolo, A., Chapman, J., Grimwood, J., Tadeo, F.R., Estornell, L.H., Munoz-Sanz, J.V., Ibanez, V., Herrero-Ortega, A., Aleza, P., Perez-Perez, J., Ramon, D., Brunel, D., Luro, F., Chen, C., Farmerie, W.G., Desany, B., Kodira, C., Mohiuddin, M., Harkins, T., Fredrikson, K., Burns, P., Lomsadze, A., Borodovsky, M., Reforgiato, G., Freitas-Astua, J., Quetier, F., Navarro, L., Roose, M., Wincker, P., Schmutz, J., Morgante, M., Machado, M.A., Talon, M., Jaillon, O., Ollitrault, P., and Gmitter, F. (2014). Sequencing of diverse mandarin, pummelo and orange genomes reveals complex history of admixture during citrus domestication. *Nature Biotechnology* 32, 656-662.
