# Supplementary material for: Cell Wall Remodeling in Abscission Zone Cells during Ethylene-Promoted Fruit Abscission in Citrus
Source: Front Plant Sci. 2017 Feb 8;8:126. doi: 10.3389/fpls.2017.00126 (PMC5296326; doi:10.3389/fpls.2017.00126)
Supplement: Figure S4 — Phylogenetic relationships between Glycoside hydrolases. [file Image4.PDF]

Figure S4. Phylogenetic relationships between Glycoside hydrolases (GHs): CELs (GH9), PGs (GH28s), β-GLUs (GH1s), β-XYLs (GH3s), β-MANs (GH5s), XTHs (GH16s), α-XYLs (GH31s), β-GALs (GH35s and GH2s) and ASDs (GH51s)

Color-coding of proteins regulated by ethylene in AZ-C cells and/or FR cells of Washington Navel maturing fruits or regulated during abscission in AZs in other plant species

CitXXXXX	Up-regulated exclusivelly in AZ-C cells
CitXXXXX	Up-regulated exclusivelly in fruit rind cells
CitXXXXX	Up-regulated in both AZ-C and fruit rind cells
CitXXXXX	Down-regulated exclusivelly in AZ-C cells
CitXXXXX	Down-regulated exclusivelly in fruit rind cells
CitXXXXX	Down-regulated in both AZ-C and fruit rind cells
CitXXXXX	Probe printed in the 20 K citrus microarray (Martínez-Godoy et al, 2008) but without hybridization results
XXXXX	Up-regulated during AZ activation in other plant species
XXXXX	Down-regulated during AZ activation in other plant species
XXXXX	β-glucuronidase (GUS) activity in floral organ AZ cells of *Arabidopsis thaliana*
LAZ	Up-regulated in LAZ-enriched tissues (Agustí et al., 2008; 2012) or preferentially expressed in LAZ cells (Agustí et al., 2009) during ethylene-promoted abscission in citrus leaves
Petiole	Up-regulated in petioles (Agustí et al., 2008) or preferentially expressed in petiolar cortical cells (Agustí et al., 2009) during ethylene-promoted abscission in citrus leaves
AZ-C tissues	Up-regulated in AZ-enriched tissues during ethylene-promoted abscission in orange fruits (Cheng et al, 2015)
AZ-C tissues	Down-regulated in AZ-enriched tissues during ethylene-promoted abscission in orange fruits (Cheng et al, 2015)
ida-2	Down-regulated in receptacles of *ida-2* plants (Liu et al, 2013)
hae-3/hsl2-3	Down-regulated in receptacles of *hae-2/hsl2-3* double mutant plants (Niederhuth et al, 2013)

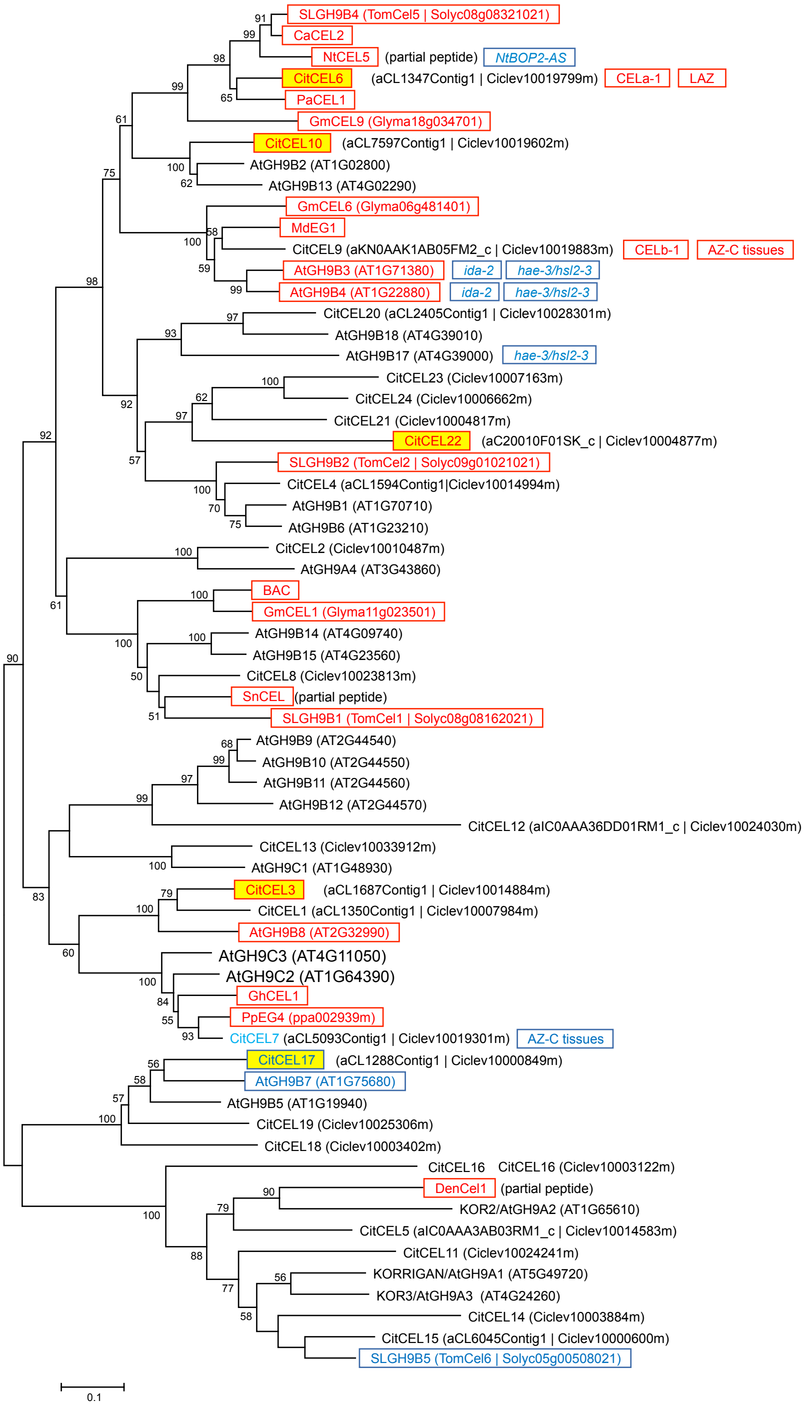

Figure S4A. Phylogenetic relationships between cellulases/endo-1,4-β-glucanases (CELs, GH9). The phylogenetic tree shows the degree of similarity between the CELs annotated in the genome of *Arabidopsis thaliana* (TAIR) and in the *Citrus clementina* haploid genome (Wu et al., 2014; Table S3) and those previously described as related to the abscission process in other plant species. Phylogenetic trees are based on multiple alignments of proteins using the profile alignment function of ClustalW (http://www.ch.embnet.org/software/ClustalW-XXL.html) and were generated with MEGA7 (Kumar et al., 2016) using the neighbor-joining algorithm with 1,000 bootstrap replicates. Only bootstrap supports higher than 50% were considered and are shown in the nodes. Transcripts of *NtCEL5* were down-regulated in the corolla base of tobacco plants over-expressing an antisense-oriented sequence of *NtBOP2* ([*NtBOP2-AS*]; Wu et al., 2012). Accession numbers for the sequences of CEL proteins regulated during organ abscission in different plant species are shown. *Arabidopsis thaliana* (Lashbrook and Cai, 2008): AtGH9B3 (AT1G71380), AtGH9B4 (AT1G22880), AtGH9B7 (AT1G75680) and AtGH9B8 (AT2G32990). Apple [*Malus X domestica*] (Li and Yuan, 2008): MdEG1 (AAQ55294). Avocado [*Persea americana*] (Tonutti et al., 1995): PaCEL1 (P05522). Bean [*Phaseolus vulgaris*] (Tucker et al., 1988): BAC (Phvul.002G160200.1). Cotton [*Gossypium hirusutum*] (Mishra et al., 2008): GhCEL1 (AAN04496). *Dendrobium* (Rungruchkanont et al., 2007): DenCel1 (ABQ81793). Elderberry [*Sambucus nigra*] (Taylor et al., 1994): SnCEL (CAA52343). Orange [*Citrus sinensis*] (Burns et al., 1998; Kazokas and Burns, 1998): CELa-1 (NP_001275794) and CELb-1 (NP_001275809). Peach [*Prunus persica*] (Trainotti et al., 2006): PpEG4 (AJ890498). Pepper [*Capsicum annum*] (Ferrarese et al., 1995): CaCEL2 (CAA65828). Soybean [*Glycine max*] (Tucker et al., 2007): GmCEL1 (Glyma11g02350.1), GmCEL6 (Glyma06g48140.1) and GmCEL9 (Glyma18g03470.1). Tobacco [*Nicotiana tabacum*] (Wu et al., 2012): NtCEL5 (AAL30456). Tomato [*Solanum lycopersicum*] (Brummell et al., 1999; del Campillo and Bennett, 1996; Lashbrook et al., 1994; Meir et al., 2010): SLGH9B1 (TomCel1/Solyc08g081620.2.1), SLGH9B2 (TomCel2/Solyc09g010210.2.1), SLGH9B4 (TomCel5/Solyc08g083210.2.1) and SLGH9B5 (TomCel6/Solyc05g005080.2.1).

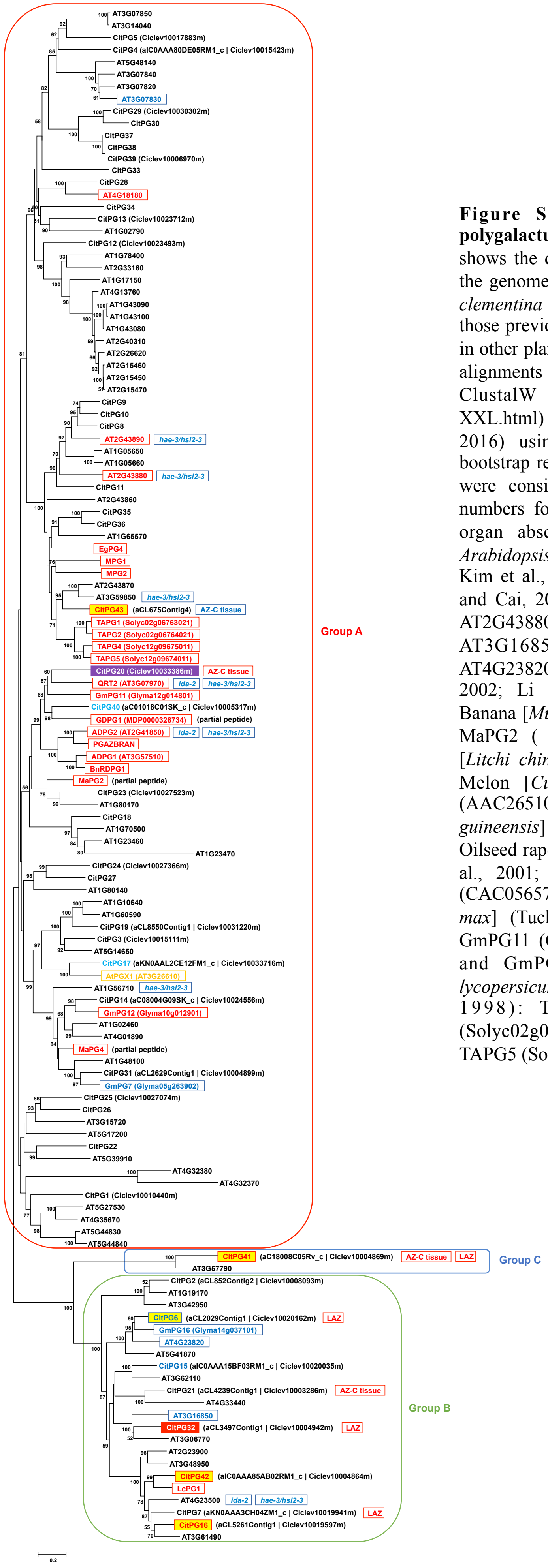

Figure S4B. Phylogenetic relationships between polygalacturonases (PGs, GH28). The phylogenetic tree shows the degree of similarity between the PGs annotated in the genome of *Arabidopsis thaliana* (TAIR) and in the *Citrus clementina* haploid genome (Wu et al., 2014; Table S3) and those previously described as related to the abscission process in other plant species. Phylogenetic trees are based on multiple alignments of proteins using the profile alignment function of ClustalW (http://www.ch.embnet.org/software/ClustalW-XXL.html) and were generated with MEGA7 (Kumar et al., 2016) using the neighbor-joining algorithm with 1,000 bootstrap replicates. Only bootstrap supports higher than 50% were considered and are shown in the nodes. Accession numbers for the sequences of PG proteins regulated during organ abscission in different plant species are shown. *Arabidopsis thaliana* (Jenkins et al., 1999; Sander et al., 2001; Kim et al., 2006; González-Carranza et al., 2007; Lashbrook and Cai, 2008; Ogawa et al., 2009): ADPG2 (AT2G41850), AT2G43880, AT2G43890, AT3G07830, QRT2 (AT3G07970), AT3G16850, ADPG1 (AT3G57510), AT4G18180 and AT4G23820. Apple [*Malus X domestica*] (Atkinson et al., 2002; Li and Yuan, 2008): GDPG1 (MDP0000326734). Banana [*Musa acuminata*] (Mbéguié-A-Mbéguié et al., 2009): MaPG2 (AAO27531) and MaPG4 (CAE51357). Lychee [*Litchi chinensis*] (Peng et al., 2013): LcPG1 (AFW04075). Melon [*Cucumis melo*] (Hadfield et al., 1998): MPG1 (AAC26510) and MPG2 (AAC26511). Oil palm [*Elaeis guineensis*] (Roongsattham et al., 2012): EgPG4 (AFO53698). Oilseed rape [*Brassica napus*] (Petersen et al., 1996; Sander et al., 2001; Gonzalez-Carranza et al., 2002): PGAZBRAN (CAC05657) and BnRDPG1 (CAA65072). Soybean [*Glycine max*] (Tucker et al., 2007): GmPG7 (Glyma05g26390.2), GmPG11 (Glyma12g01480.1), GmPG12 (Glyma10g01290.1) and GmPG16 (Glyma14g03710.1). Tomato [*Solanum lycopersicum*] (Kalaitzis et al., 1995, 1997; Hong and Tucker, 1998): TAPG1 (Solyc02g067630.2.1), TAPG2 (Solyc02g067640.2.1), TAPG4 (Solyc12g096750.1.1) and TAPG5 (Solyc12g096740.1.1).

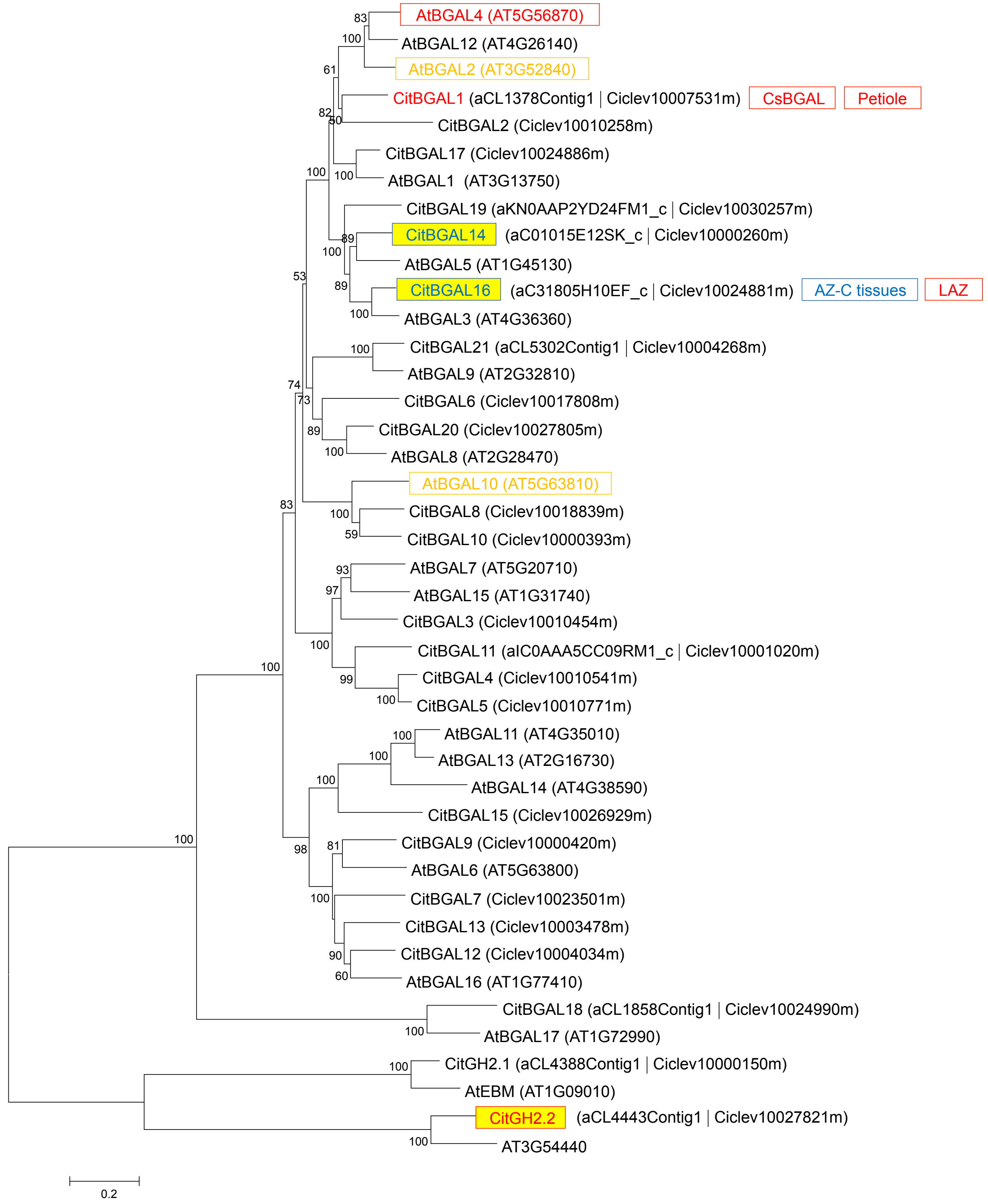

Figure S4C. Phylogenetic relationships between β-galactosidases (BGALs, GH35, GH2). The phylogenetic tree shows the degree of similarity between the PLs annotated in the genome of *Arabidopsis thaliana* (TAIR) and in the *Citrus clementina* haploid genome (Wu et al., 2014; Table S3) and those previously described as related to the abscission process in other plant species. Phylogenetic trees are based on multiple alignments of proteins using the profile alignment function of ClustalW (http://www.ch.embnet.org/software/ClustalW-XXL.html) and were generated with MEGA7 (Kumar et al., 2016) using the neighbor-joining algorithm with 1,000 bootstrap replicates. Only bootstrap supports higher than 50% were considered and are shown in the nodes. Family 2 of Glycoside Hydrolases (GH2s) gathers enzymes with different known enzymatic activities including β-galactosidase activity (EC 3.2.1.23). In Arabidopsis, AtEBM (AT1G09010) has endo-β-mannosidase activity and AT3G5444 has β-galactosidase activity. Accession numbers for the sequences of BGAL proteins regulated during organ abscission in different plant species are shown. *Arabidopsis thaliana* (Lashbrook and Cai, 2008): AtBGAL4 (AT5G56870). Orange [*Citrus sinensis*] (Wu and Burns, 2004): CsBGAL (NP_001275845). GUS activity in floral organ AZs according to Albornos et al. (2012).

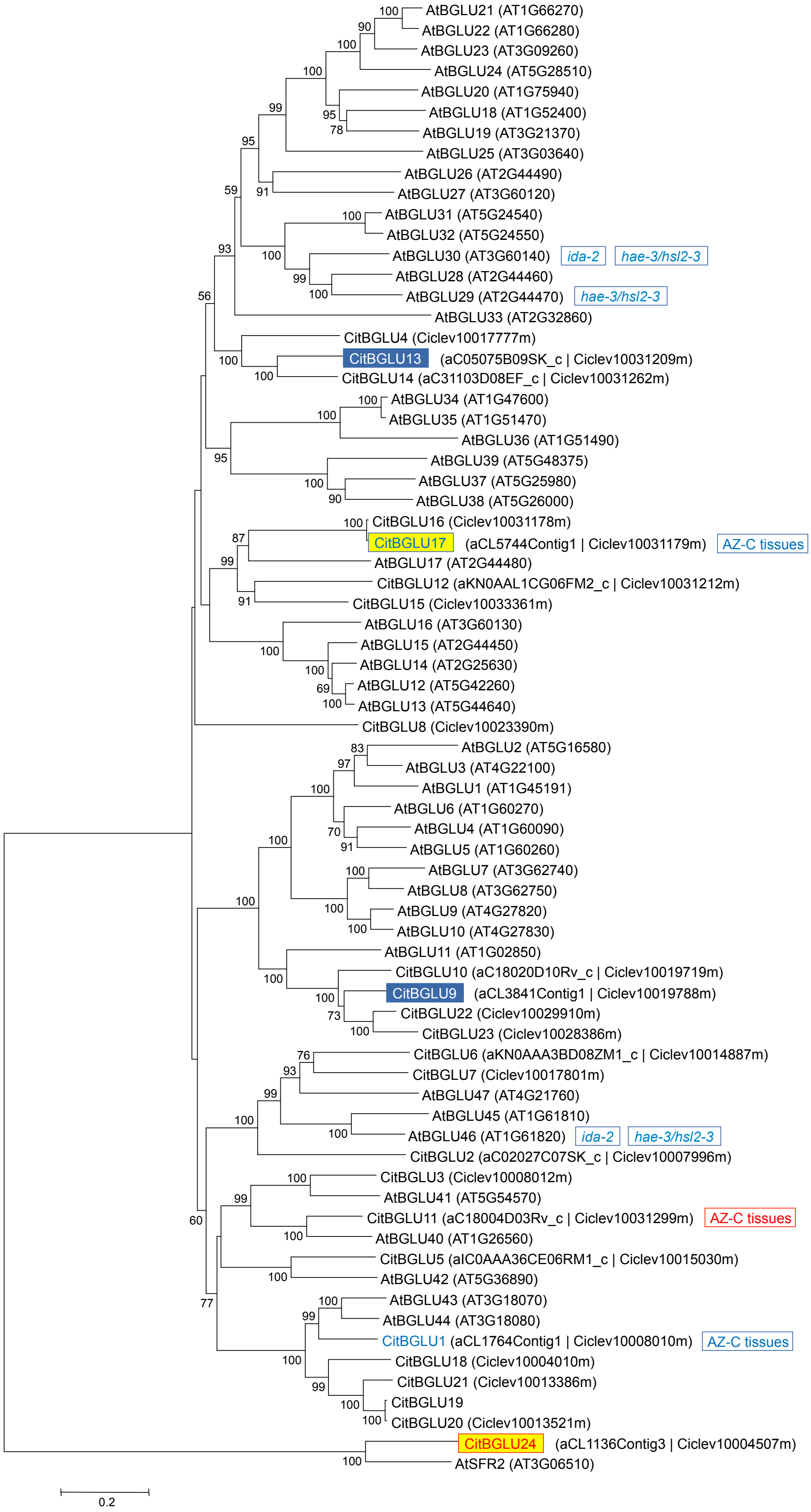

Figure S4D. Phylogenetic relationships between β-glucosidases (BGLUs, GH1). The phylogenetic tree shows the degree of similarity between the BGLUs annotated in the genome of *Arabidopsis thaliana* (TAIR), the BGLUs annotated in the *Citrus clementina* haploid genome (Wu et al., 2014; Table S3) and the BGLUs previously described as related to the abscission process in other plant species are shown. Phylogenetic trees are based on multiple alignments of proteins using the profile alignment function of ClustalW (http://www.ch.embnet.org/software/ClustalW-XXL.html) and were generated with MEGA7 (Kumar et al., 2016) using the neighbor-joining algorithm with 1,000 bootstrap replicates. Only bootstrap supports higher than 50% were considered and are shown in the nodes.

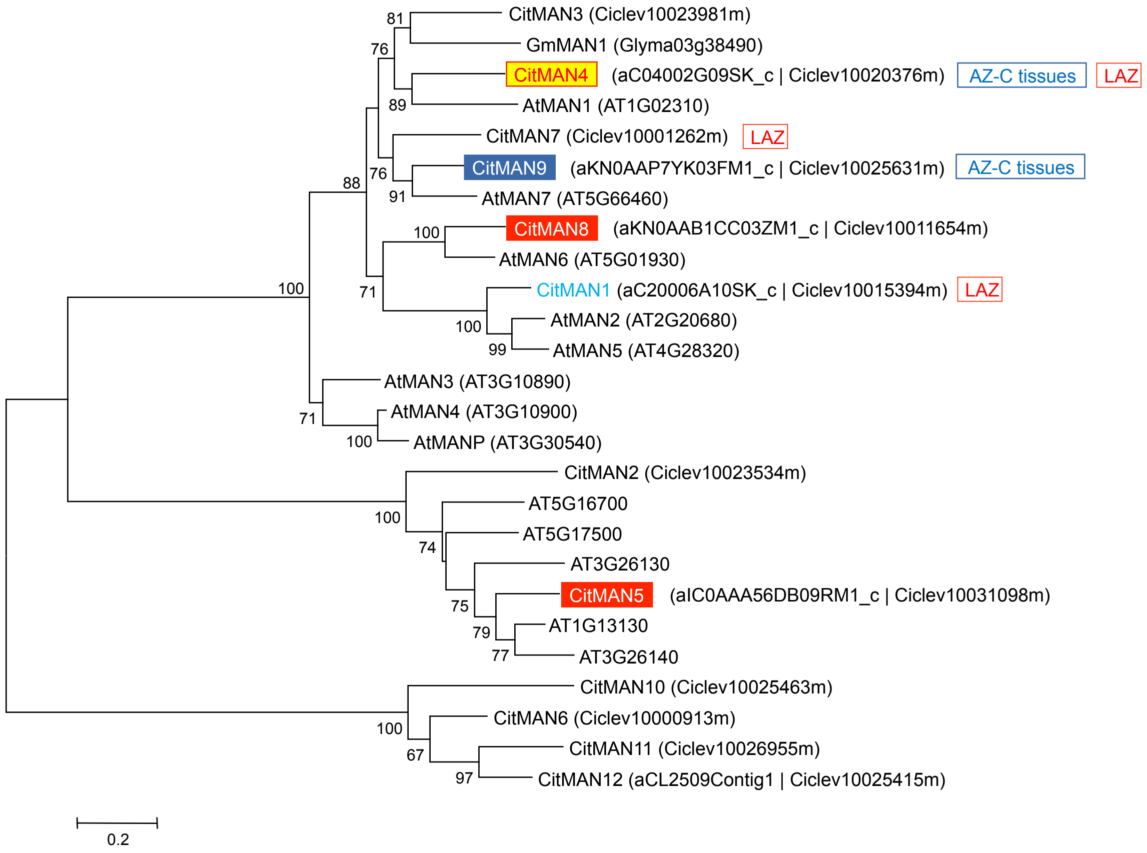

Figure S4E. Phylogenetic relationships between endo-β-mannanases (MANs, GH5). The phylogenetic tree shows the degree of similarity between the MANs annotated in the genome of *Arabidopsis thaliana* (TAIR) and in the *Citrus clementina* haploid genome (Wu et al., 2014; Table S3) and those previously described as related to the abscission process in other plant species. Phylogenetic trees are based on multiple alignments of proteins using the profile alignment function of ClustalW (http://www.ch.embnet.org/software/ClustalW-XXL.html) and were generated with MEGA7 (Kumar et al., 2016) using the neighbor-joining algorithm with 1,000 bootstrap replicates. Only bootstrap supports higher than 50% were considered and are shown in the nodes. Accession numbers for the sequences of MAN proteins regulated during organ abscission in different plant species: soybean [*Glycine max*] (Yan et al., 2012): GmMAN1 (Glyma03g38490). The expression of GmMAN1 was induced in both petiole-AZs and non-AZs during petiole abscission in explants although was not induced in soybean plants treated with ethephon.

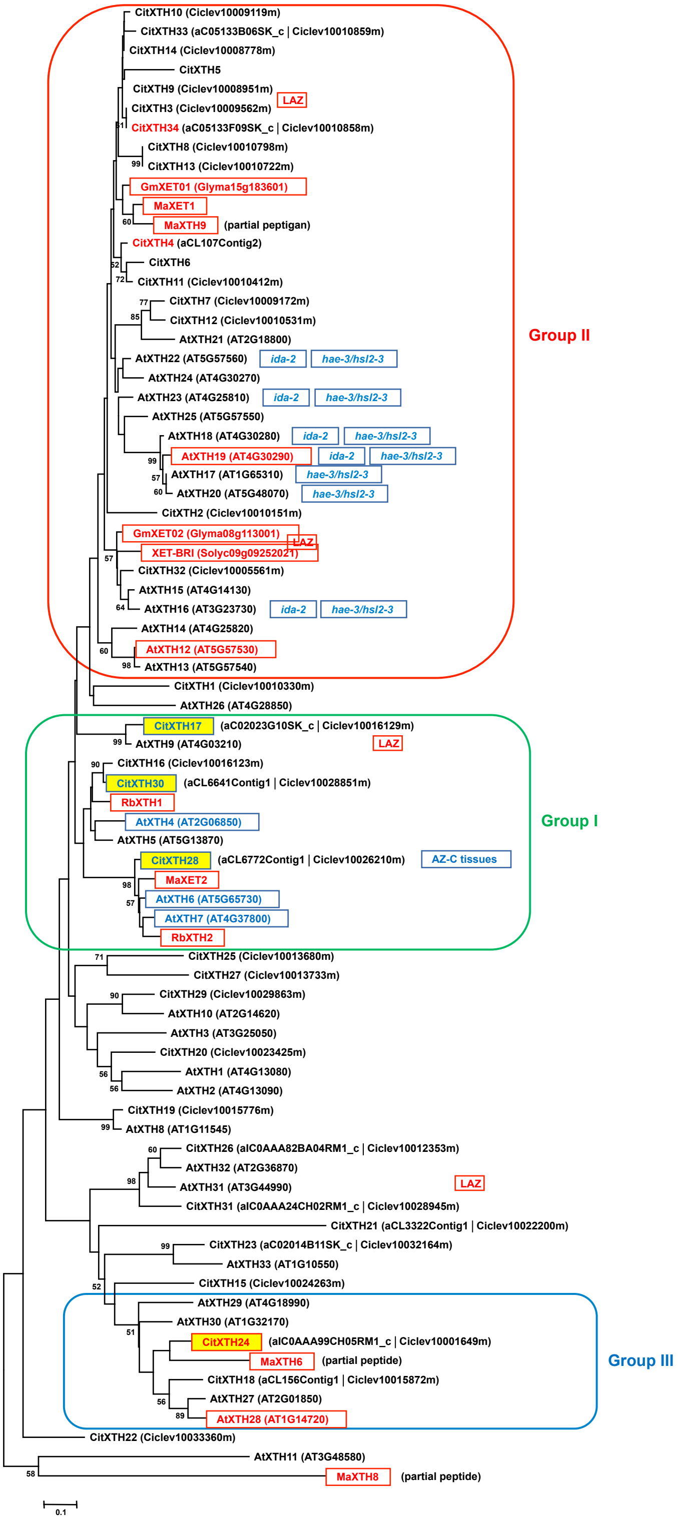

Figure S4F. Phylogenetic relationships between xyloglucan endotranglycosylases / hydrolases (XTHs, GH16). The phylogenetic tree shows the degree of similarity between the XTHs annotated in the genome of *Arabidopsis thaliana* (TAIR) and in the *Citrus clementina* haploid genome (Wu et al., 2014; Table S3) and those previously described as related to the abscission process in other plant species. Phylogenetic trees are based on multiple alignments of proteins using the profile alignment function of ClustalW (http://www.ch.embnet.org/software/ClustalW-XXL.html) and were generated with MEGA7 (Kumar et al., 2016) using the neighbor-joining algorithm with 1,000 bootstrap replicates. Only bootstrap supports higher than 50% were considered and are shown in the nodes. Accession numbers for the sequences of XTH proteins regulated during organ abscission in different plant species are shown. *Arabidopsis thaliana* (Lashbrook and Cai, 2008): AtXTH4 (AT2G06850), AtXTH6 (AT5G65730), AtXTH7 (AT4G37800), AtXTH12 (AT5G57530), AtXTH19 (AT4G30290) and AtXTH28 (AT1G14720). Banana [*Musa acuminata*] (Mbéguié-A-Mbéguié et al., 2009): MaXET1 (ABL10090), MaXET2 (ABL10091), MaXTH6 (ACQ85268), MaXTH8 (ACQ85270) and MaXTH9 (ACQ85271). Rose [*Rosa buorboniana*] (Singh et al., 2011): RbXTH1 (ABB86296) and RbXTH2 (ABC55454). Soybean [*Glycine max*] (Tucker et al., 2007): GmXET01 (Glyma15g18360.1) and GmXET02 (Glyma08g11300.1). Tomato [*Solanum lycopersicum*] (Meir et al., 2010): XET-BRI (Solyc09g092520.2.1).

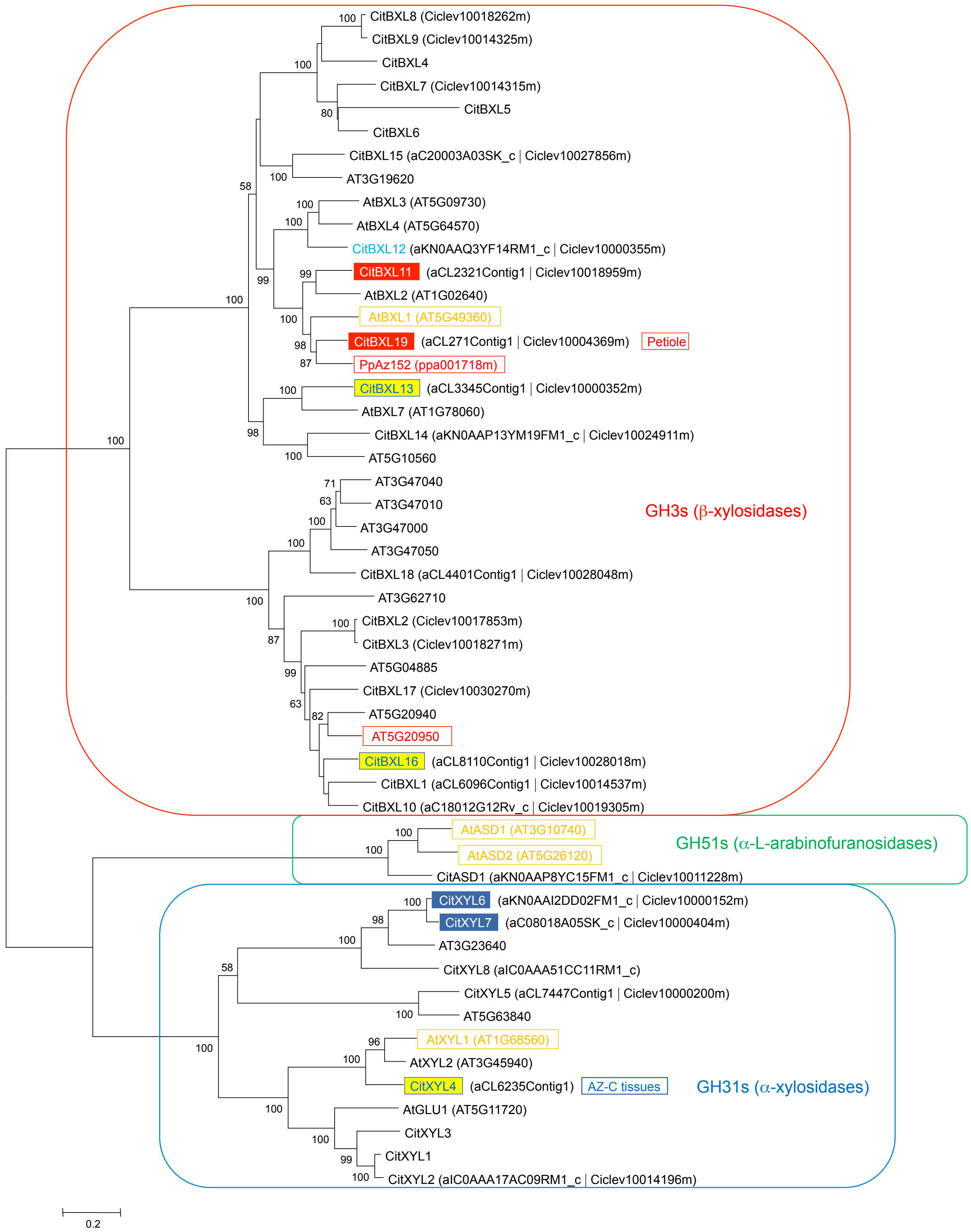

Figure S4G. Phylogenetic relationships between β-xylosidases (BXLs, GH3), α-xylosidases (XYLs, GH31) and α-arabinofuranosidases (ASDs, GH51). The phylogenetic tree shows the degree of similarity between the BXLs, XYLs and ASDs annotated in the genome of *Arabidopsis thaliana* (TAIR) and in the *Citrus clementina* haploid genome (Wu et al., 2014; Table S3) and those previously described as related to the abscission process in other plant species. Phylogenetic trees are based on multiple alignments of proteins using the profile alignment function of ClustalW (http://www.ch.embnet.org/software/ClustalW-XXL.html) and were generated with MEGA7 (Kumar et al., 2016) using the neighbor-joining algorithm with 1,000 bootstrap replicates. Only bootstrap supports higher than 50% were considered and are shown in the nodes. Accession numbers for the sequences of BXL, XYL and ASD proteins regulated during organ abscission in different plant species are shown. *Arabidopsis thaliana* (Goujon et al., 2003; Lashbrook and Cai, 2008): AtBXL1 (AT5G49360) and AT5G20950. Peach [*Prunus persica*] (Ruperti et al., 2002): PpAz152 (ppa001718m). *Arabidopsis thaliana* (Sampedro et al., 2010): AtXYL1 (AT1G68560). *Arabidopsis thaliana* (Fulton and Cobbett, 2003): AtASD1 (AT3G10740) and AtASD2 (AT5G26120).

REFERENCES

Agustí, J., Gimeno, J., Merelo, P., Serrano, R., Cercos, M., Conesa, A., Talon, M., and Tadeo, F.R. (2012). Early gene expression events in the laminar abscission zone of abscission-promoted citrus leaves after a cycle of water stress/rehydration: involvement of CitbHLH1. *J Exp Bot* 63**,** 6079-6091.

Agustí, J., Merelo, P., Cercos, M., Tadeo, F.R., and Talon, M. (2008). Ethylene-induced differential gene expression during abscission of citrus leaves. *J Exp Bot* 59**,** 2717-2733.

Agustí, J., Merelo, P., Cercos, M., Tadeo, F.R., and Talon, M. (2009). Comparative transcriptional survey between laser-microdissected cells from laminar abscission zone and petiolar cortical tissue during ethylene-promoted abscission in citrus leaves. *BMC Plant Biol* 9**,** 127.

Albornos, L., Martín, I., Pérez, P., Marcos, R., Dopico, B., and Labrador, E. (2012). Promoter activities of genes encoding β-galactosidases from Arabidopsis a1 subfamily. *Plant physiology and biochemistry* 60**,** 223-232.

Atkinson, R.G., Schroder, R., Hallett, I.C., Cohen, D., and Macrae, E.A. (2002). Overexpression of polygalacturonase in transgenic apple trees leads to a range of novel phenotypes involving changes in cell adhesion. *Plant Physiol* 129**,** 122-133.

Brummell, D.A., Hall, B.D., and Bennett, A.B. (1999). Antisense suppression of tomato endo-1,4-beta-glucanase Cel2 mRNA accumulation increases the force required to break fruit abscission zones but does not affect fruit softening. *Plant Mol Biol* 40**,** 615-622.

Burns, J.K., Lewandowski, D.J., Nairn, C.J., and Brown, G.E. (1998). Endo-1,4-β-glucanase gene expression and cell wall hydrolase activities during abscission in Valencia orange. *Physiologia Plantarum* 102**,** 217-225.

Cheng, C., Zhang, L., Yang, X., and Zhong, G. (2015). Profiling gene expression in citrus fruit calyx abscission zone (AZ-C) treated with ethylene. *Mol Genet Genomics* 290**,** 1991-2006.

Del Campillo, E., and Bennett, A.B. (1996). Pedicel breakstrength and cellulase gene expression during tomato flower abscission. *Plant Physiol* 111**,** 813-820.

Ferrarese, L., Trainotti, L., Moretto, P., Polverino De Laureto, P., Rascio, N., and Casadoro, G. (1995). Differential ethylene-inducible expression of cellulase in pepper plants. *Plant Mol Biol* 29**,** 735-747.

Fulton, L.M., and Cobbett, C.S. (2003). Two α-l-arabinofuranosidase genes in Arabidopsis thaliana are differentially expressed during vegetative growth and flower development*. *Journal of Experimental Botany* 54**,** 2467-2477.

Gonzalez-Carranza, Z.H., Elliott, K.A., and Roberts, J.A. (2007). Expression of polygalacturonases and evidence to support their role during cell separation processes in Arabidopsis thaliana. *J Exp Bot* 58**,** 3719-3730.

Gonzalez-Carranza, Z.H., Whitelaw, C.A., Swarup, R., and Roberts, J.A. (2002). Temporal and spatial expression of a polygalacturonase during leaf and flower abscission in oilseed rape and Arabidopsis. *Plant Physiol* 128**,** 534-543.

Goujon, T., Minic, Z., El Amrani, A., Lerouxel, O., Aletti, E., Lapierre, C., Joseleau, J.P., and Jouanin, L. (2003). AtBXL1, a novel higher plant (Arabidopsis thaliana) putative beta-xylosidase gene, is involved in secondary cell wall metabolism and plant development. *Plant J* 33**,** 677-690.

Hadfield, K.A., Rose, J.K.C., Yaver, D.S., Berka, R.M., and Bennett, A.B. (1998). Polygalacturonase Gene Expression in Ripe Melon Fruit Supports a Role for Polygalacturonase in Ripening-Associated Pectin Disassembly. *Plant Physiology* 117**,** 363-373.

Hong, S.B., and Tucker, M.L. (1998). Genomic organization of six tomato polygalacturonases and 5' upstream sequence identity with tap1 and win2 genes. *Mol Gen Genet* 258**,** 479-487.

Jenkins, E.S., Paul, W., Craze, M., Whitelaw, C.A., Weigand, A., and Roberts, J.A. (1999). Dehiscence-related expression of an Arabidopsis thaliana gene encoding a polygalacturonase in transgenic plants of Brassica napus. *Plant, Cell & Environment* 22**,** 159-167.

Kalaitzis, P., Koehler, S.M., and Tucker, M.L. (1995). Cloning of a tomato polygalacturonase expressed in abscission. *Plant Mol Biol* 28**,** 647-656.

Kalaitzis, P., Solomos, T., and Tucker, M.L. (1997). Three different polygalacturonases are expressed in tomato leaf and flower abscission, each with a different temporal expression pattern. *Plant Physiol* 113**,** 1303-1308.

Kazokas, W.C., and Burns, J.K. (1998). Cellulase activity and gene expression in citrus fruit abscission zones during and after ethylene treatment. *Journal of the American Society for Horticultural Science* 123**,** 781-786.

Kim, J., Shiu, S.-H., Thoma, S., Li, W.-H., and Patterson, S.E. (2006). Patterns of expansion and expression divergence in the plant polygalacturonase gene family. *Genome Biology* 7**,** R87-R87.

Kumar, S., Stecher, G., and Tamura, K. (2016). MEGA7: Molecular Evolutionary Genetics Analysis version 7.0 for bigger datasets. *Molecular Biology and Evolution*.

Lashbrook, C.C., and Cai, S. (2008). Cell wall remodeling in Arabidopsis stamen abscission zones: Temporal aspects of control inferred from transcriptional profiling. *Plant Signaling & Behavior* 3**,** 733-736.

Lashbrook, C.C., Gonzalez-Bosch, C., and Bennett, A.B. (1994). Two divergent endo-beta-1,4-glucanase genes exhibit overlapping expression in ripening fruit and abscising flowers. *The Plant Cell* 6**,** 1485-1493.

Letunic, I., Doerks, T., and Bork, P. (2009). SMART 6: recent updates and new developments. *Nucleic Acids Res* 37**,** D229-232.

Li, J., and Yuan, R. (2008). NAA and Ethylene Regulate Expression of Genes Related to Ethylene Biosynthesis, Perception, and Cell Wall Degradation During Fruit Abscission and Ripening in 'Delicious' Apples. *Journal of Plant Growth Regulation* 27**,** 283.

Liu, B., Butenko, M.A., Shi, C.L., Bolivar, J.L., Winge, P., Stenvik, G.E., Vie, A.K., Leslie, M.E., Brembu, T., Kristiansen, W., Bones, A.M., Patterson, S.E., Liljegren, S.J., and Aalen, R.B. (2013). NEVERSHED and INFLORESCENCE DEFICIENT IN ABSCISSION are differentially required for cell expansion and cell separation during floral organ abscission in Arabidopsis thaliana. *J Exp Bot* 64**,** 5345-5357.

Lombard, V., Golaconda Ramulu, H., Drula, E., Coutinho, P.M., and Henrissat, B. (2014). The carbohydrate-active enzymes database (CAZy) in 2013. *Nucleic Acids Res* 42**,** D490-495.

Martinez-Godoy, M.A., Mauri, N., Juarez, J., Marques, M.C., Santiago, J., Forment, J., and Gadea, J. (2008). A genome-wide 20 K citrus microarray for gene expression analysis. *BMC Genomics* 9**,** 318.

Meir, S., Philosoph-Hadas, S., Sundaresan, S., Selvaraj, K.S., Burd, S., Ophir, R., Kochanek, B., Reid, M.S., Jiang, C.Z., and Lers, A. (2010). Microarray analysis of the abscission-related transcriptome in the tomato flower abscission zone in response to auxin depletion. *Plant Physiol* 154**,** 1929-1956.

Minic, Z., Rihouey, C., Do, C.T., Lerouge, P., and Jouanin, L. (2004). Purification and Characterization of Enzymes Exhibiting β-d-Xylosidase Activities in Stem Tissues of Arabidopsis. *Plant Physiology* 135**,** 867-878.

Mishra, A., Khare, S., Trivedi, P.K., and Nath, P. (2008). Ethylene induced cotton leaf abscission is associated with higher expression of cellulase (GhCel1) and increased activities of ethylene biosynthesis enzymes in abscission zone. *Plant Physiol Biochem* 46**,** 54-63.

Niederhuth, C.E., Patharkar, O.R., and Walker, J.C. (2013). Transcriptional profiling of the Arabidopsis abscission mutant hae hsl2 by RNA-Seq. *BMC Genomics* 14**,** 37.

Ogawa, M., Kay, P., Wilson, S., and Swain, S.M. (2009). ARABIDOPSIS DEHISCENCE ZONE POLYGALACTURONASE1 (ADPG1), ADPG2, and QUARTET2 are Polygalacturonases required for cell separation during reproductive development in Arabidopsis. *Plant Cell* 21**,** 216-233.

Peng, G., Wu, J., Lu, W., and Li, J. (2013). A polygalacturonase gene clustered into clade E involved in lychee fruitlet abscission. *Scientia Horticulturae* 150**,** 244-250.

Petersen, M., Sander, L., Child, R., Van Onckelen, H., Ulvskov, P., and Borkhardt, B. (1996). Isolation and characterisation of a pod dehiscence zone-specific polygalacturonase from Brassica napus. *Plant Mol Biol* 31**,** 517-527.

Roongsattham, P., Morcillo, F., Jantasuriyarat, C., Pizot, M., Moussu, S., Jayaweera, D., Collin, M., Gonzalez-Carranza, Z.H., Amblard, P., Tregear, J.W., Tragoonrung, S., Verdeil, J.L., and Tranbarger, T.J. (2012). Temporal and spatial expression of polygalacturonase gene family members reveals divergent regulation during fleshy fruit ripening and abscission in the monocot species oil palm. *BMC Plant Biol* 12**,** 150.

Rungruchkanont, K., Ketsa, S., Chatchawankanphanich, O., and Van Doorn, W.G. (2007). Endogenous auxin regulates the sensitivity of Dendrobium (cv. Miss Teen) flower pedicel abscission to ethylene. *Functional Plant Biology* 34**,** 885-894.

Sampedro, J., Pardo, B., Gianzo, C., Guitian, E., Revilla, G., and Zarra, I. (2010). Lack of alpha-xylosidase activity in Arabidopsis alters xyloglucan composition and results in growth defects. *Plant Physiol* 154**,** 1105-1115.

Sander, L., Child, R., Ulvskov, P., Albrechtsen, M., and Borkhardt, B. (2001). Analysis of a dehiscence zone endo-polygalacturonase in oilseed rape (Brassica napus) and Arabidopsis thaliana: evidence for roles in cell separation in dehiscence and abscission zones, and in stylar tissues during pollen tube growth. *Plant Mol Biol* 46**,** 469-479.

Singh, A.P., Pandey, S.P., Rajluxmi, Pandey, S., Nath, P., and Sane, A.P. (2011). Transcriptional activation of a pectate lyase gene, RbPel1, during petal abscission in rose. *Postharvest Biology and Technology* 60**,** 143-148.

Taylor, J.E., Coupe, S.A., Picton, S., and Roberts, J.A. (1994). Characterization and accumulation pattern of an mRNA encoding an abscission-related beta-1,4-glucanase from leaflets of Sambucus nigra. *Plant Mol Biol* 24**,** 961-964.

Taylor, J.E., Coupe, S.A., Picton, S., and Roberts, J.A. (1994). Characterization and accumulation pattern of an mRNA encoding an abscission-related beta-1,4-glucanase from leaflets of Sambucus nigra. *Plant Mol Biol* 24**,** 961-964.

Tonutti, P., Cass, L.G., and Christoffersen, R.E. (1995). The expression of cellulase gene family members during induced avocado fruit abscission and ripening. *Plant, Cell & Environment* 18**,** 709-713.

Tucker, M.L., Burke, A., Murphy, C.A., Thai, V.K., and Ehrenfried, M.L. (2007). Gene expression profiles for cell wall-modifying proteins associated with soybean cyst nematode infection, petiole abscission, root tips, flowers, apical buds, and leaves. *Journal of Experimental Botany* 58**,** 3395-3406.

Tucker, M.L., Sexton, R., Del Campillo, E., and Lewis, L.N. (1988). Bean abscission cellulase : characterization of a cDNA clone and regulation of gene expression by ethylene and auxin. *Plant Physiol* 88**,** 1257-1262.

Wu, G.A., Prochnik, S., Jenkins, J., Salse, J., Hellsten, U., Murat, F., Perrier, X., Ruiz, M., Scalabrin, S., Terol, J., Takita, M.A., Labadie, K., Poulain, J., Couloux, A., Jabbari, K., Cattonaro, F., Del Fabbro, C., Pinosio, S., Zuccolo, A., Chapman, J., Grimwood, J., Tadeo, F.R., Estornell, L.H., Munoz-Sanz, J.V., Ibanez, V., Herrero-Ortega, A., Aleza, P., Perez-Perez, J., Ramon, D., Brunel, D., Luro, F., Chen, C., Farmerie, W.G., Desany, B., Kodira, C., Mohiuddin, M., Harkins, T., Fredrikson, K., Burns, P., Lomsadze, A., Borodovsky, M., Reforgiato, G., Freitas-Astua, J., Quetier, F., Navarro, L., Roose, M., Wincker, P., Schmutz, J., Morgante, M., Machado, M.A., Talon, M., Jaillon, O., Ollitrault, P., and Gmitter, F. (2014). Sequencing of diverse mandarin, pummelo and orange genomes reveals complex history of admixture during citrus domestication. *Nature Biotechnology* 32**,** 656-662.

Wu, X.-M., Yu, Y., Han, L.-B., Li, C.-L., Wang, H.-Y., Zhong, N.-Q., Yao, Y., and Xia, G.-X. (2012). The Tobacco BLADE-ON-PETIOLE2 Gene Mediates Differentiation of the Corolla Abscission Zone by Controlling Longitudinal Cell Expansion. *Plant Physiology* 159**,** 835-850.

Wu, Z., and Burns, J.K. (2004). A beta-galactosidase gene is expressed during mature fruit abscission of 'Valencia' orange (Citrus sinensis). *J Exp Bot* 55**,** 1483-1490.
