# Supplementary material for: Cell Wall Remodeling in Abscission Zone Cells during Ethylene-Promoted Fruit Abscission in Citrus
Source: Front Plant Sci. 2017 Feb 8;8:126. doi: 10.3389/fpls.2017.00126 (PMC5296326; doi:10.3389/fpls.2017.00126)
Supplement: Figure S6 — Phylogenetic relationships between Expansins. [file Image6.PDF]

Figure S6. Phylogenetic relationships between Expansins.

Color-coding of proteins regulated by ethylene in AZ-C cells and/or FR cells of Washington Navel maturing fruits or regulated during abscission in AZs in other plant species

CitXXXXX	Up-regulated exclusivelly in AZ-C cells
CitXXXXX	Up-regulated exclusivelly in fruit rind cells
CitXXXXX	Up-regulated in both AZ-C and fruit rind cells
CitXXXXX	Down-regulated exclusivelly in AZ-C cells
CitXXXXX	Down-regulated exclusivelly in fruit rind cells
CitXXXXX	Down-regulated in both AZ-C and fruit rind cells
CitXXXXX	Probe printed in the 20 K citrus microarray (Martínez-Godoy et al, 2008) but without hybridization results
XXXXX	Up-regulated during AZ activation in other plant species
XXXXX	Down-regulated during AZ activation in other plant species
XXXXX	β-glucuronidase (GUS) activity in floral organ AZ cells of *Arabidopsis thaliana*
LAZ	Up-regulated in LAZ-enriched tissues (Agustí et al., 2008; 2012) or preferentially expressed in LAZ cells (Agustí et al., 2009) during ethylene-promoted abscission in citrus leaves
Petiole	Up-regulated in petioles (Agustí et al., 2008) or preferentially expressed in petiolar cortical cells (Agustí et al., 2009) during ethylene-promoted abscission in citrus leaves
AZ-C tissues	Up-regulated in AZ-enriched tissues during ethylene-promoted abscission in orange fruits (Cheng et al, 2015)
AZ-C tissues	Down-regulated in AZ-enriched tissues during ethylene-promoted abscission in orange fruits (Cheng et al, 2015)
ida-2	Down-regulated in receptacles of *ida-2* plants (Liu et al, 2013)
hae-3/hsl2-3	Down-regulated in receptacles of *hae-2/hsl2-3* double mutant plants (Niederhuth et al, 2013)

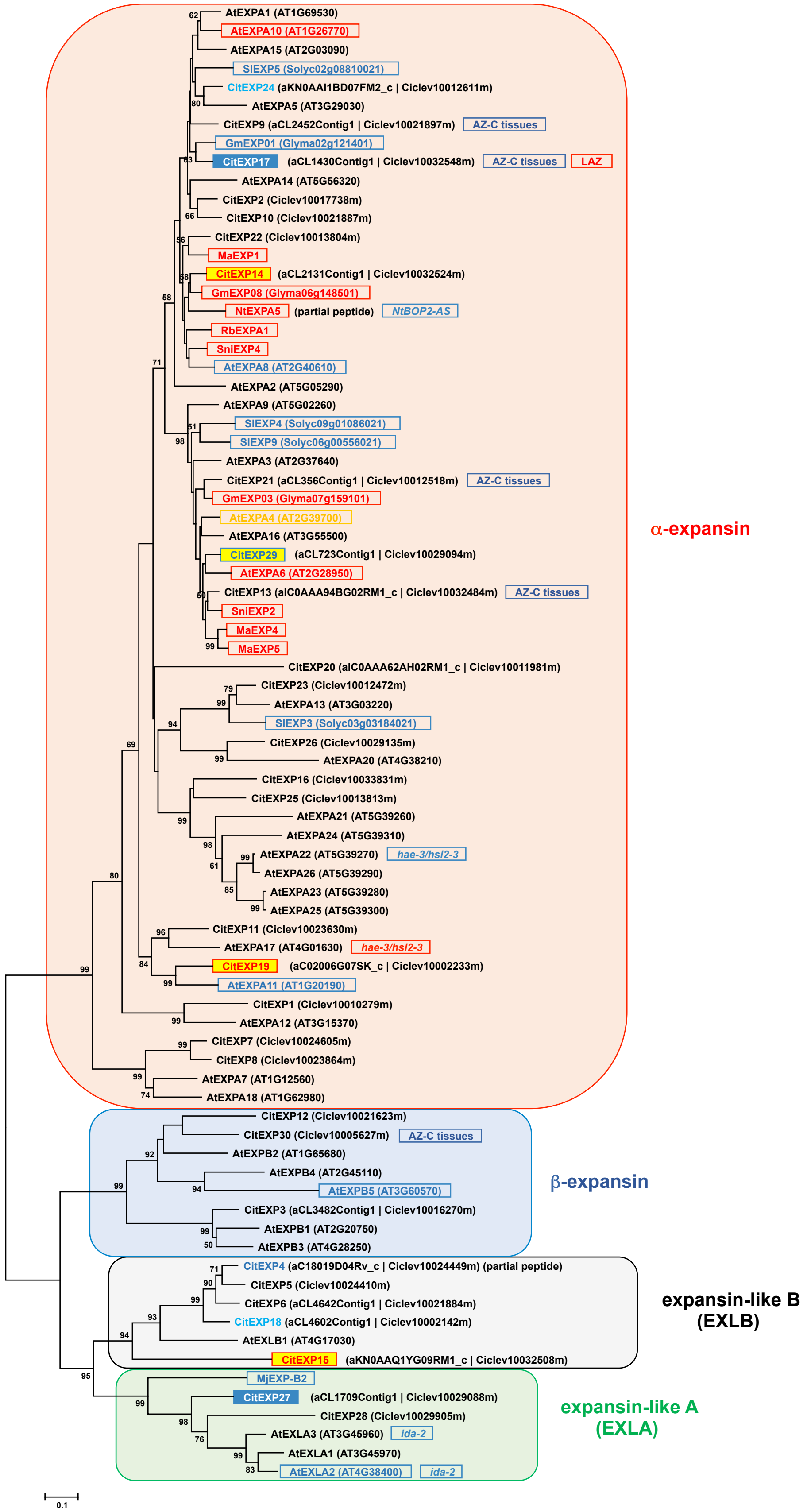

Figure S6. Phylogenetic relationships between expansins (EXPs). The phylogenetic tree shows the degree of similarity between the EXPs annotated in the genome of *Arabidopsis thaliana* (TAIR) and in the *Citrus clementina* haploid genome (Wu et al, 2014; Table S3) and those previously described as related to the abscission process in other plant species. Phylogenetic trees are based on multiple alignments of proteins using the profile alignment function of ClustalW (http://wwwchembnetorg/software/ClustalW-XXLhtml) and were generated with MEGA7 (Kumar et al, 2016) using the neighbor-joining algorithm with 1,000 bootstrap replicates. Only bootstrap supports higher than 50% were considered and are shown in the nodes. Transcripts of NtEXPA5 were down-regulated in the corolla base of tobacco plants over-expressing an antisense-oriented sequence of NtBOP2 ([NtBOP2-AS]; Wu et al, 2012). Accession numbers for the sequences of EXP proteins regulated during organ abscission in AZs of different plant species are shown. *Arabidopsis thaliana* (Cho and Cosgrove, 2000; González-Carranza et al., 2012; Lashbrook and Cai, 2008): AtEXPA4 (AT2G39700), AtEXPA6 (AT2G28950), AtEXPA8 (AT2G40610), AtEXPA10 (AT1G26770), AtEXPA11 (AT1G20190), AtEXPB5 (AT3G60570) and AtEXLA2 (AT4G38400). Elderberry [*Sambucus nigra*] (Belfield et al, 2005): SniEXP2 (AAP48989) and SniEXP4 (AAP48991). Soybean [*Glycine max*] (Tucker et al, 2007): GmEXP1 (Glyma02g121401), GmEXP3 (Glyma07g159101) and GmEXP8 (Glyma06g148501). Banana [*Musa acuminata*] (Mbéguié-A-Mbéguié et al, 2009): MaEXP1 (AAM08930), MaEXP4 (ABN09939) and MaEXP5 (ABN09940). Rose [*Rosa bourboniana*] (Sane et al, 2007): RbEXPA1 (ABC55453). The four o'clock flower [*Mirabilis jalapa*] (Meir et al, 2006): MjEXP-B2 (AAN86683). Tomato [*Solanum lycopersicum*] (Meir et al, 2010): SlEXP3 (SGN-U576599), SlEXP4 (SGN-U580310), SlEXP5 (SGN-U581279) and SlEXP9 (SGN-U580110). GUS activity in floral organ AZs according to González-Carranza et al. (2012).

REFERENCES

Agustí, J., Gimeno, J., Merelo, P., Serrano, R., Cercos, M., Conesa, A., Talon, M., and Tadeo, F.R. (2012). Early gene expression events in the laminar abscission zone of abscission-promoted citrus leaves after a cycle of water stress/rehydration: involvement of CitbHLH1. *J Exp Bot* 63, 6079-6091.

Agustí, J., Merelo, P., Cercos, M., Tadeo, F.R., and Talon, M. (2008). Ethylene-induced differential gene expression during abscission of citrus leaves. *J Exp Bot* 59, 2717-2733.

Agustí, J., Merelo, P., Cercos, M., Tadeo, F.R., and Talon, M. (2009). Comparative transcriptional survey between laser-microdissected cells from laminar abscission zone and petiolar cortical tissue during ethylene-promoted abscission in citrus leaves. *BMC Plant Biol* 9, 127.

Belfield, E.J., Ruperti, B., Roberts, J.A., and Mcqueen-Mason, S. (2005). Changes in expansin activity and gene expression during ethylene-promoted leaflet abscission in Sambucus nigra. *Journal of Experimental Botany* 56, 817-823.

Cheng, C., Zhang, L., Yang, X., and Zhong, G. (2015). Profiling gene expression in citrus fruit calyx abscission zone (AZ-C) treated with ethylene. *Mol Genet Genomics* 290, 1991-2006.

Cho, H.T., and Cosgrove, D.J. (2000). Altered expression of expansin modulates leaf growth and pedicel abscission in Arabidopsis thaliana. *Proc Natl Acad Sci U S A* 97, 9783-9788.

Gonzalez-Carranza, Z.H., Shahid, A.A., Zhang, L., Liu, Y., Ninsuwan, U., and Roberts, J.A. (2012). A novel approach to dissect the abscission process in Arabidopsis. *Plant Physiol* 160, 1342-1356.

Kumar, S., Stecher, G., and Tamura, K. (2016). MEGA7: Molecular Evolutionary Genetics Analysis version 7.0 for bigger datasets. *Molecular Biology and Evolution*.

Lashbrook, C.C., and Cai, S. (2008). Cell wall remodeling in Arabidopsis stamen abscission zones: Temporal aspects of control inferred from transcriptional profiling. *Plant Signaling & Behavior* 3, 733-736.

Liu, B., Butenko, M.A., Shi, C.L., Bolivar, J.L., Winge, P., Stenvik, G.E., Vie, A.K., Leslie, M.E., Brembu, T., Kristiansen, W., Bones, A.M., Patterson, S.E., Liljegren, S.J., and Aalen, R.B. (2013). NEVERSHED and INFLORESCENCE DEFICIENT IN ABSCISSION are differentially required for cell expansion and cell separation during floral organ abscission in Arabidopsis thaliana. *J Exp Bot* 64, 5345-5357.

Martinez-Godoy, M.A., Mauri, N., Juarez, J., Marques, M.C., Santiago, J., Forment, J., and Gadea, J. (2008). A genome-wide 20 K citrus microarray for gene expression analysis. *BMC Genomics* 9, 318.

Mbéguié-a-Mbéguié, D., Hubert, O., Baurens, F.C., Matsumoto, T., Chillet, M., Fils-Lycaon, B., and Sidibé-Bocs, S. (2009). Expression patterns of cell wall-modifying genes from banana during fruit ripening and in relationship with finger drop. *Journal of Experimental Botany* 60, 2021-2034.

Meir, S., Hunter, D.A., Chen, J.C., Halaly, V., and Reid, M.S. (2006). Molecular changes occurring during acquisition of abscission competence following auxin depletion in Mirabilis jalapa. *Plant Physiol* 141, 1604-1616.

Meir, S., Philosoph-Hadas, S., Sundaresan, S., Selvaraj, K.S., Burd, S., Ophir, R., Kochanek, B., Reid, M.S., Jiang, C.Z., and Lers, A. (2010). Microarray analysis of the abscission-related transcriptome in the tomato flower abscission zone in response to auxin depletion. *Plant Physiol* 154, 1929-1956.

Niederhuth, C.E., Patharkar, O.R., and Walker, J.C. (2013). Transcriptional profiling of the Arabidopsis abscission mutant hae hsl2 by RNA-Seq. *BMC Genomics* 14, 37.

Sane, A.P., Tripathi, S.K., and Nath, P. (2007). Petal abscission in rose (Rosa bourboniana var Gruss an Teplitz) is associated with the enhanced expression of an alpha expansin gene, RbEXPA1. *Plant Science* 172, 481-487.

Tucker, M.L., Burke, A., Murphy, C.A., Thai, V.K., and Ehrenfried, M.L. (2007). Gene expression profiles for cell wall-modifying proteins associated with soybean cyst nematode infection, petiole abscission, root tips, flowers, apical buds, and leaves. *Journal of Experimental Botany* 58, 3395-3406.

Wu, G.A., Prochnik, S., Jenkins, J., Salse, J., Hellsten, U., Murat, F., Perrier, X., Ruiz, M., Scalabrin, S., Terol, J., Takita, M.A., Labadie, K., Poulain, J., Couloux, A., Jabbari, K., Cattonaro, F., Del Fabbro, C., Pinosio, S., Zuccolo, A., Chapman, J., Grimwood, J., Tadeo, F.R., Estornell, L.H., Munoz-Sanz, J.V., Ibanez, V., Herrero-Ortega, A., Aleza, P., Perez-Perez, J., Ramon, D., Brunel, D., Luro, F., Chen, C., Farmerie, W.G., Desany, B., Kodira, C., Mohiuddin, M., Harkins, T., Fredrikson, K., Burns, P., Lomsadze, A., Borodovsky, M., Reforgiato, G., Freitas-Astua, J., Quetier, F., Navarro, L., Roose, M., Wincker, P., Schmutz, J., Morgante, M., Machado, M.A., Talon, M., Jaillon, O., Ollitrault, P., and Gmitter, F. (2014). Sequencing of diverse mandarin, pummelo and orange genomes reveals complex history of admixture during citrus domestication. *Nature Biotechnology* 32, 656-662.

Wu, X.-M., Yu, Y., Han, L.-B., Li, C.-L., Wang, H.-Y., Zhong, N.-Q., Yao, Y., and Xia, G.-X. (2012). The Tobacco BLADE-ON-PETIOLE2 Gene Mediates Differentiation of the Corolla Abscission Zone by Controlling Longitudinal Cell Expansion. *Plant Physiology* 159, 835-850.
