# Supplementary material for: Cell Wall Remodeling in Abscission Zone Cells during Ethylene-Promoted Fruit Abscission in Citrus
Source: Front Plant Sci. 2017 Feb 8;8:126. doi: 10.3389/fpls.2017.00126 (PMC5296326; doi:10.3389/fpls.2017.00126)
Supplement: Figure S7 — Phylogenetic relationships between members of gene families associated with monolignol biosynthesis and polymerization. [file Image7.PDF]

Figure S7. Phylogenetic relationships between members of gene families associated with monolignol biosynthesis and polymerization.

Color-coding of proteins regulated by ethylene in AZ-C cells and/or FR cells of Washington Navel maturing fruits or regulated during abscission in AZs in other plant species

CitXXXXX	Up-regulated exclusivelly in AZ-C cells
CitXXXXX	Up-regulated exclusivelly in fruit rind cells
CitXXXXX	Up-regulated in both AZ-C and fruit rind cells
CitXXXXX	Down-regulated exclusivelly in AZ-C cells
CitXXXXX	Down-regulated exclusivelly in fruit rind cells
CitXXXXX	Down-regulated in both AZ-C and fruit rind cells
CitXXXXX	Probe printed in the 20 K citrus microarray (Martínez-Godoy et al, 2008) but without hybridization results
XXXXX	Up-regulated during AZ activation in other plant species
XXXXX	Down-regulated during AZ activation in other plant species
XXXXX	β-glucuronidase (GUS) activity in floral organ AZ cells of *Arabidopsis thaliana*
AZ-C tissues	Up-regulated in AZ-enriched tissues during ethylene-promoted abscission in orange fruits (Cheng et al, 2015)
AZ-C tissues	Down-regulated in AZ-enriched tissues during ethylene-promoted abscission in orange fruits (Cheng et al, 2015)
ida-2	Down-regulated in receptacles of *ida-2* plants (Liu et al, 2013)
hae-3/hsl2-3	Down-regulated in receptacles of *hae-2/hsl2-3* double mutant plants (Niederhuth et al, 2013)

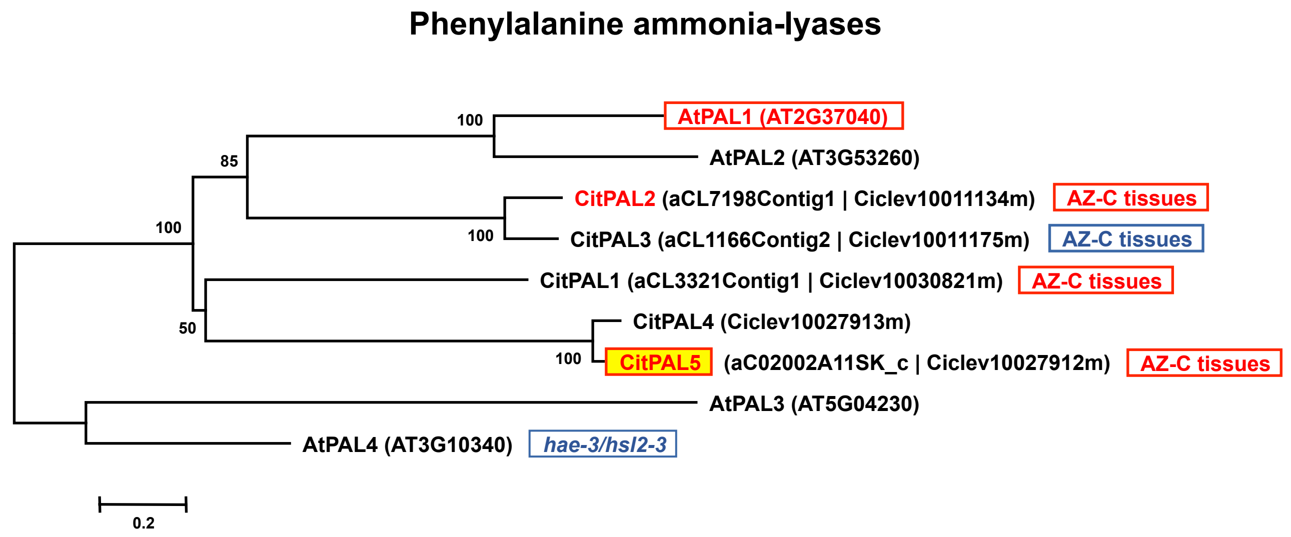

Figure S7A. Phylogenetic relationships between phenylalanine ammonia-lyases (PALs), the enzymes involved in the initial step of the general phenylpropanoid pathway. The synthesis of monolignols starts with the deamination of phenylalanine to form cinnamic acid, which is the first step in the general phenylpropanoid pathway catalyzed by PALs. They are encoded by a small gene family in plants with four members in Arabidopsis (Raes et al., 2003). A large number of studies have shown that PALs are responsive to a variety of environmental stimuli, including pathogen infection, wounding, nutrient depletion, extreme temperatures, and other stress conditions (Zhang and Liu, 2015). Interestingly, organ abscission is regulated by the plant physiological and developmental status and biotic and abiotic environmental conditions (Estornell et al., 2013). Molecular analysis shows that two Arabidopsis PAL genes, *AtPAL1* and *AtPAL4*, are regulated in AZ cells during floral organ abscission. The histochemical staining of transgenic Arabidopsis lines expressing the promoter of *AtPAL1* fused to the β-glucuronidase (GUS) reporter gene shows GUS activity in floral organs AZs (Ohl et al., 1990). In addition, *AtPAL1* is up-regulated in stamen AZ cells during stamen abscission (Cai and Lashbrook, 2008) and *AtPAL4* is down-regulated in the floral receptacles of double *hae hsl2* mutant plants (Niederhuth et al., 2013) suggesting that this gene may be up-regulated in floral organ AZs during abscission. The PAL gene family in citrus apparently consist of six members. *CitPAL5* (aC02002A11SK_c|Ciclev10027912m) is the only PAL gene exclusively regulated (up-regulated) in AZ-C cells during ethylene-promoted fruit abscission. Another citrus PAL gene, *CitPAL2* (aCL7198Contig1|Ciclev10011134m), is also up-regulated by ethylene but in FR cells close but not adjacent to the AZ-C cells. It has been recently reported that *CitPAL5* together with *CitPAL1* and *CitPAL2* were up-regulated whereas *CitPAL3* was down-regulated in orange fruit AZ-enriched tissues by ethephon (Cheng et al., 2015).

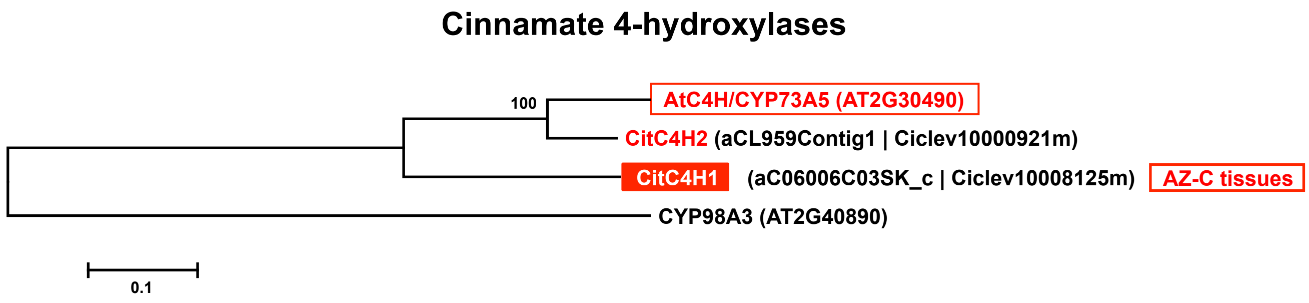

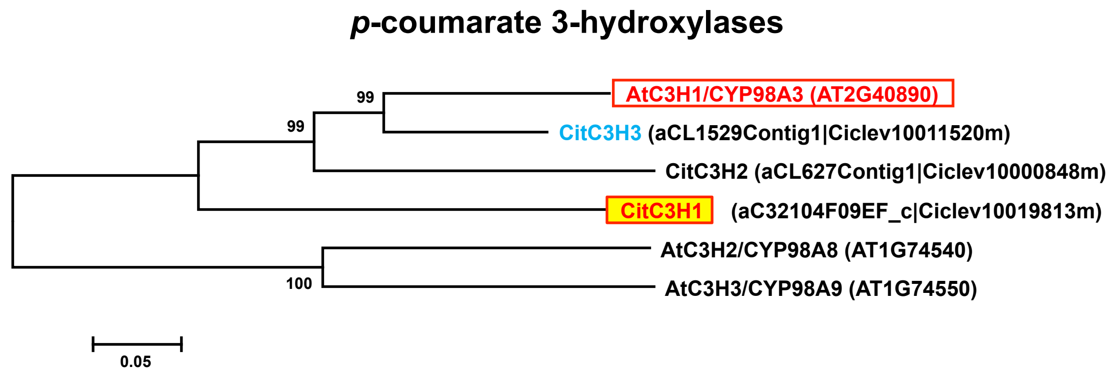

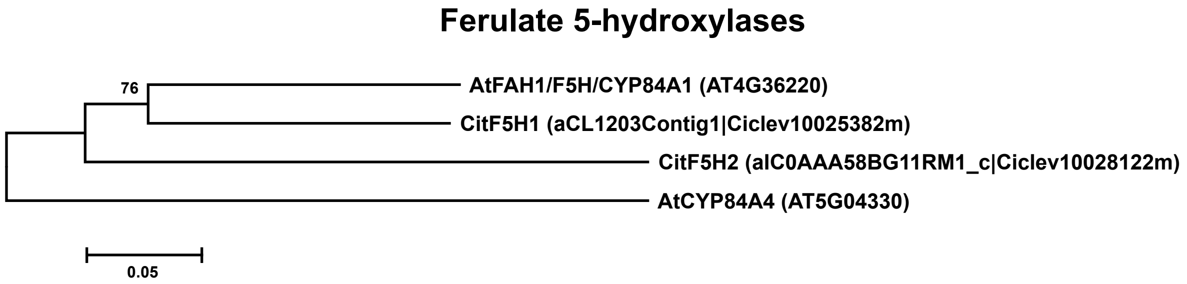

Figure S7B. Phylogenetic relationships of cytochrome P450 enzymes (CYP450s) playing a role in lignin biosynthesis. Three specific cytochrome P450 enzymes (CYP450s; cinnamate 4-hydroxylases, C4H; *p*-coumarate 3-hydroxylases, C3H; ferulate 5-hydroxylases, F5H), all belonging to the class II type CYP450s, catalyze the corresponding 4-, 3-, and 5-hydroxylations of the aromatic ring. In Arabidopsis, the tissue expression profiling of both C4H and C3H reveal an intense β-glucuronidase (GUS) activity in the receptacle of Arabidopsis flowers (Bell-Lelong et al., 1997; Nair et al., 2002) and also up-regulation in stamen AZ cells during floral organ abscission (Cai and Lashbrook, 2008). Recent studies show that C4H and C3H co-localize in the endoplasmic reticulum, forming protein complexes that interact with the soluble HCT and 4CL. These findings suggest the existence of a cluster of membrane proteins acting as a scaffold for further looser associations of soluble partners, leading to the creation of dynamic metabolons that drive the synthesis of a specific monolignol (Chen et al., 2011; Bassard et al., 2012). Recently, it has been reported that the citrus gene *CitC4H1* is up-regulated by ethephon in orange fruit AZ-C-enriched tissues (Cheng et al., 2015) although our survey shows that it is up-regulated by ethylene in both AZ-C and FR cells. In addition, *CitC3H1* (aC32104F09EF_c|Ciclev10019813m) is exclusively regulated (up-regulated) in AZ-C cells during ethylene-promoted abscission in our gene expression survey.

Hydroxycinnamoyl CoA:shikimate/quinate hydroxycinnamoyltransferases

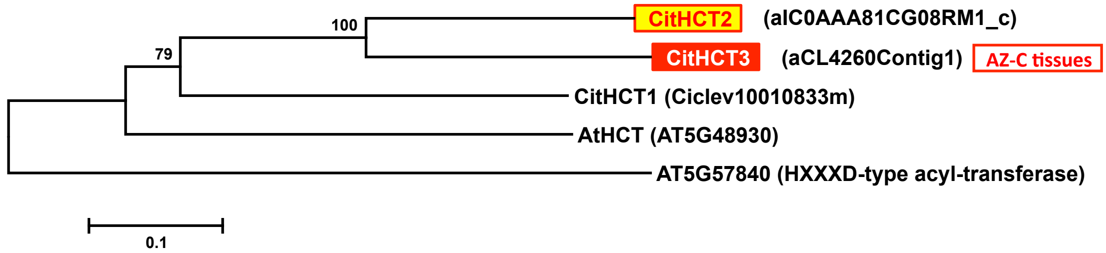

Figure S7C. Phylogenetic relationships between hydroxycinnamoyl CoA:shikimate/quinate hydroxycinnamoyltransferases (HCTs). The C4H-C3H protein complex, in conjunction with HCTs, hydroxylate 4-coumaroyl-CoA using shikimate or quinate as an acyl acceptor, at the 3 position to caffeoyl-CoA. These specific *p*-coumarate esters then serve as the substrates for the phenylpropanoid meta-hydroxylation catalyzed by C3'Hs. HCTs belong to the BAHD acyltransferase superfamily, which has extensively diversified in plants. In contrast to Arabidopsis, there are three HCTs in citrus. *CitHCT3* (aCL4260Contig1) is regulated (up-regulated) in both AZ-C and FR cells during ethylene-promoted abscission in our gene expression survey and also in citrus fruit AZ-C-enriched tissues treated with ethephon (Cheng et al., 2015) and *CitHCT2* (aIC0AAA81CG08RM1_c) is also up-regulated in our survey but exclusively in AZ-C cells.

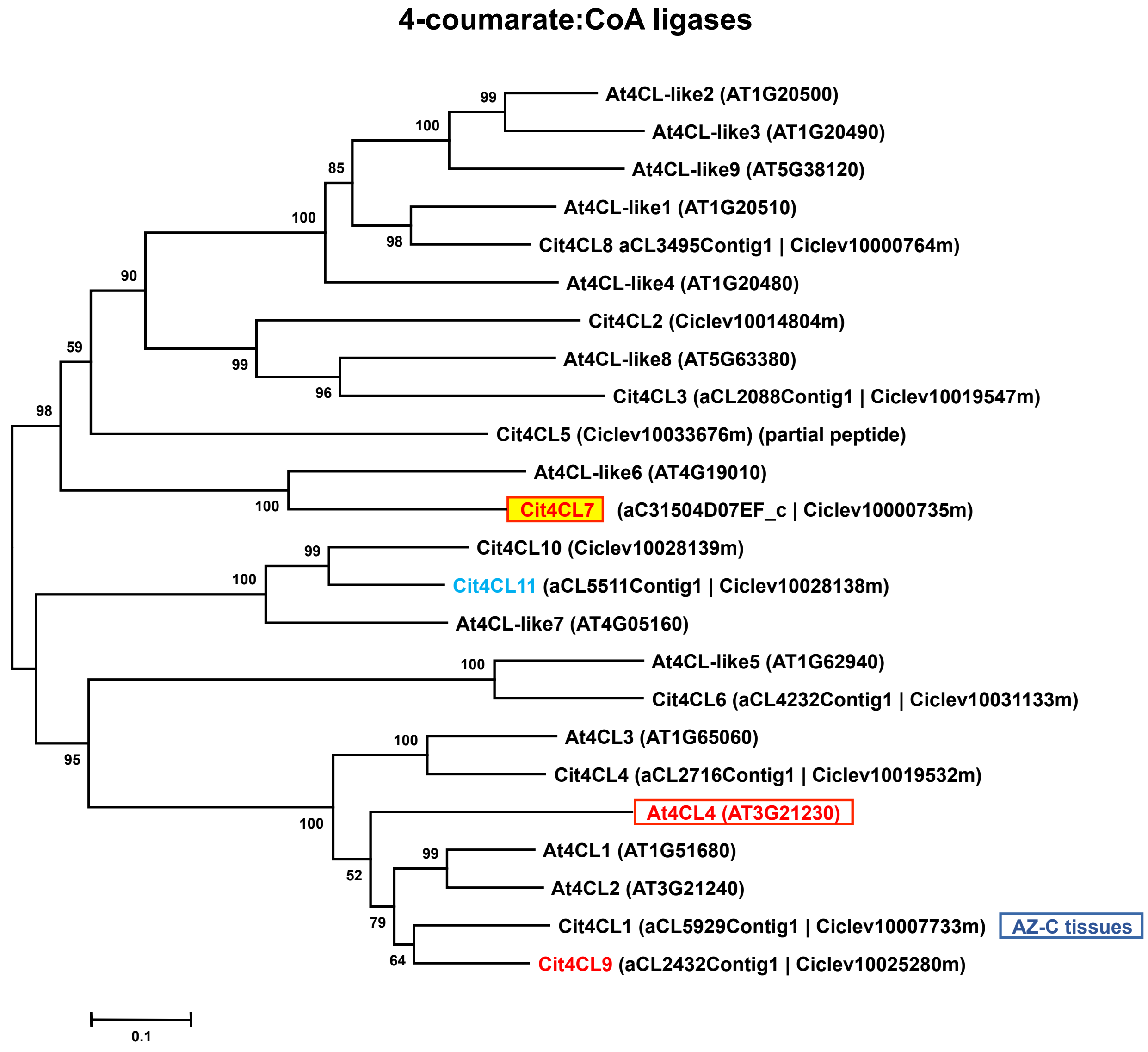

Figure S7D. Phylogenetic relationships between 4-coumarate:CoA ligases (4CLs). The next step in the phenylpropanoid pathway is the activation of the hydroxycinnamic acids to their corresponding CoA thioesters catalyzed by 4CLs. Organ abscission is regulated by plant physiological and developmental status, and biotic and abiotic environmental conditions (Estornell et al., 2013) and molecular analysis shows that one Arabidopsis 4CL gene, *At4CL4*, is up-regulated in stamen abscission zone cells during stamen abscission (Cai and Lashbrook, 2008). The 4CL gene family in citrus apparently consist of eleven members and *Cit4CL7* (aC31504D07EF_c| Ciclev10000735m) is exclusively regulated (up-regulated) in AZ-C cells during ethylene-promoted abscission. Recently, it has been reported that the citrus gene *Cit4CL1* is down-regulated by ethephon in citrus fruit AZ-C-enriched tissues (Cheng et al., 2015) although our survey shows that it is not regulated by ethylene neither in AZ-C or FR cells.

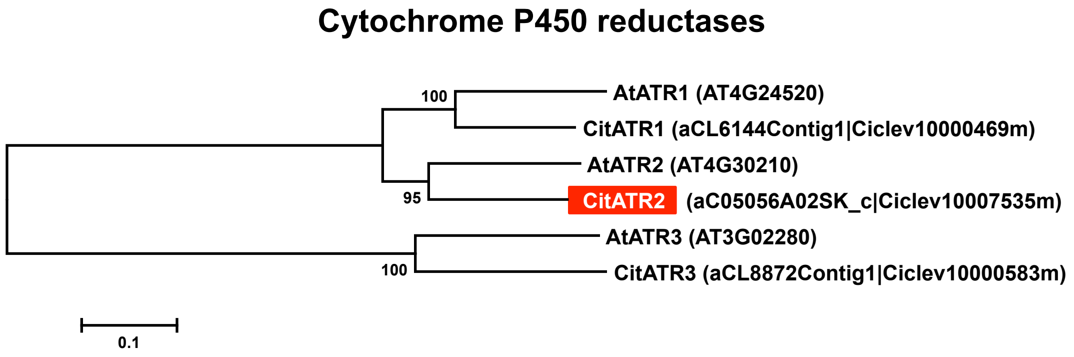

Figure S7E. Phylogenetic relationships between Cytochrome P450 reductases (CPRs). The reactions catalyzed by cytochrome P450s of the class II type rely on Cytochrome P450 reductase (CPR) activity to transfer electrons from NADPH (Jensen and Møller, 2010). Arabidopsis has only three CPR genes that are annotated as ARABIDOPSIS THALIANA P450 REDUCTASE1 (ATR1), ATR2, and ATR3 (Urban et al., 1997; Varadarajan et al., 2010). Of these three genes, ATR1 and ATR2 encode genuine CPR proteins. The expression of ATR2, but not that of ATR1, is correlated with the expression of a number of genes from the general phenylpropanoid and monolignol-specific pathways (Ehlting et al., 2005; Soitamo et al., 2008; Sundin et al., 2014). This co-expression pattern suggests that ATR2 provides electrons preferentially to the three cytochrome P450 enzymes participating in the biosynthesis of lignin, making ATR2 as a putative good marker of the lignification process. In fact, the *atr2* mutation resulted in a 6% reduction in total lignin amount in the main inflorescence stem of Arabidopsis plants (Sundin et al., 2014). As in the case of Arabidopsis, the CPR gene family in citrus also contained three members. *CitATR2* (aC05056A02SK_c|Ciclev10007535m) is the only member of this gene family regulated by ethylene during citrus fruit abscission. *CitATR2* is up-regulated in both AZ-C and fruit rind cells in the first 24 hours of fruit exposure to ethylene.

Caffeoyl-CoA 3-*O*-methyltransferases

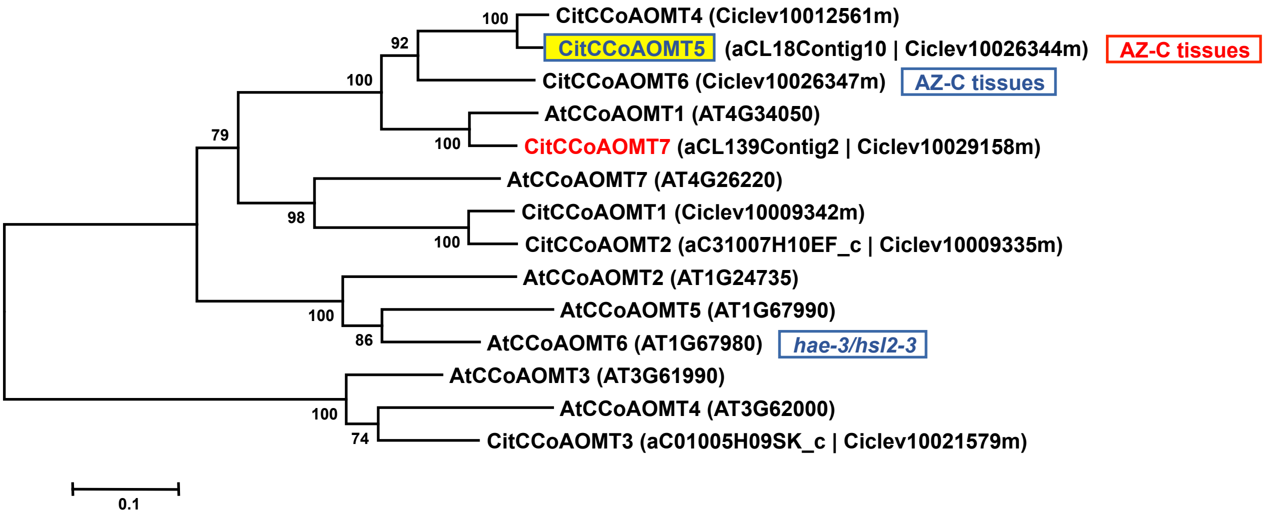

Figure S7F. Phylogenetic relationships between caffeoyl-CoA 3-*O*-methyltransferases (CCoAOMTs). CCoAOMTs produce the first transmethylation reaction on the aromatic 3-hydroxyl using S-adenosyl methionine as the methyl donor. CCoAOMTs convert caffeoyl-substituted intermediates to much more stable guaiacyl-substituted compounds, resulting in a ring modification pattern suitable for lignin biosynthesis. Organ abscission is regulated by plant physiological and developmental status, and biotic and abiotic environmental conditions (Estornell et al., 2013) and molecular analysis shows that one Arabidopsis CCoAOMT gene, *AtCCoAOMT6*, is down-regulated in the floral receptacles of double *hae hsl2* mutant plants (Niederhuth et al., 2013) suggesting that this gene may be up-regulated in floral organ abscission zones during abscission. The CCoAOMT gene family in citrus apparently consist of seven members and *CitCCoAOMT5* (aCL18Contig10|Ciclev10026344m) is exclusively regulated (down-regulated) in AZ-C cells during ethylene-promoted abscission in our gene expression survey. On the contrary, it has been recently reported that this gene is up-regulated by ethephon in citrus fruit AZ-C-enriched tissues (Cheng et al., 2015) whereas another citrus CCoAOMT phylogenetically related, *CitCCoAOMT7*, is down-regulated.

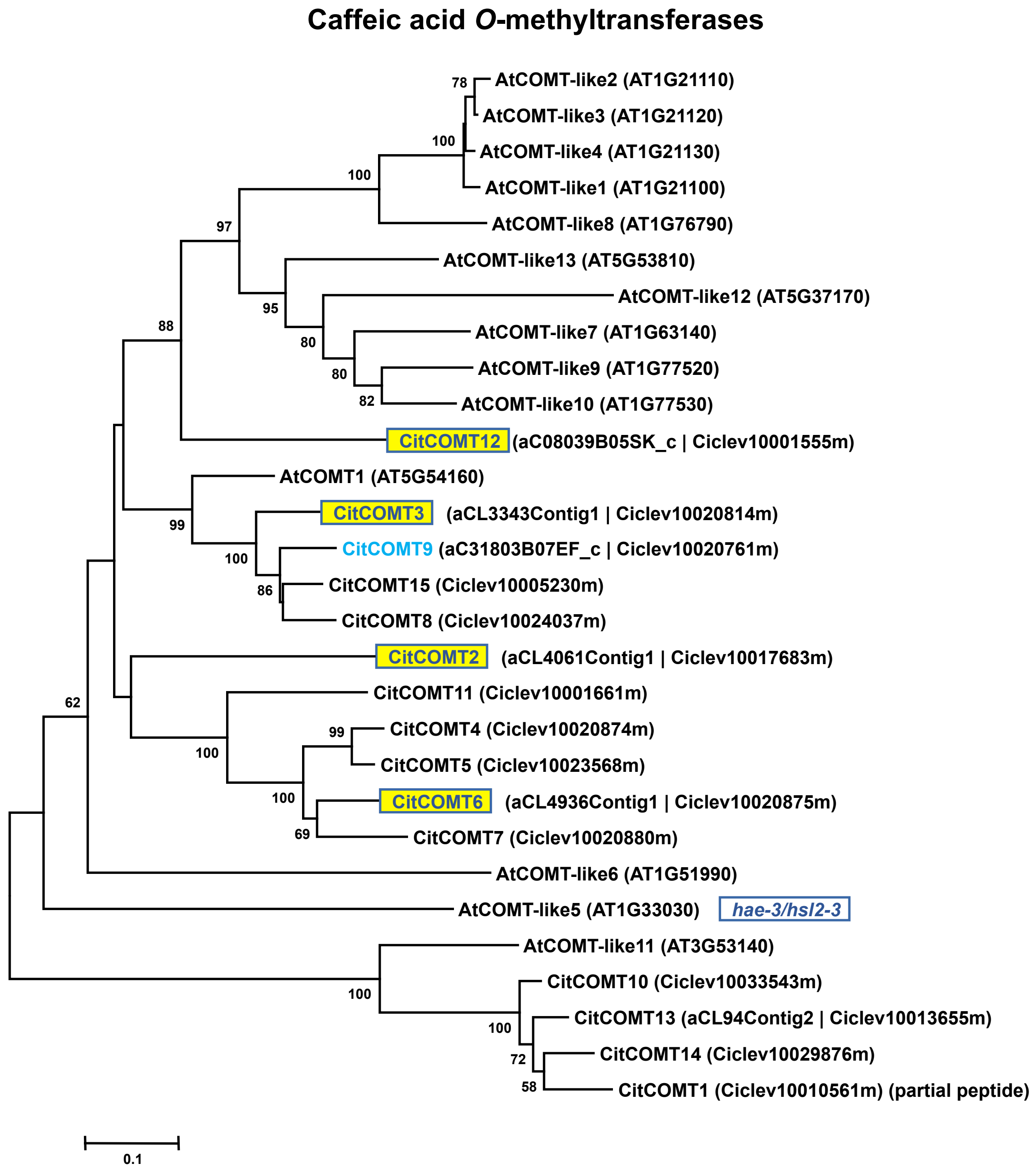

Figure S7G. Phylogenetic relationships between Caffeic acid/5-hydroxyferulic acid O-methyltransferases (COMTs). COMTs act at the level of the aldehyde and alcohol precursors of S lignin by methylating 5-hydroxy-coniferaldehyde and 5-hydroxyconiferyl alcohol to yield sinapaldehyde and sinapyl alcohol, respectively. Organ abscission is regulated by stressful environmental conditions (Estornell et al., 2013) and molecular analysis shows that one Arabidopsis COMT gene, *AtCMT-like5*, is down-regulated in the floral receptacles of double *hae hsl2* mutant plants (Niederhuth et al., 2013) suggesting that this gene may be up-regulated in floral organ abscission zones during abscission. The COMT gene family in citrus apparently consist of fifteen members and four of them are regulated (down-regulated) in AZ-C cells during ethylene-promoted abscission.

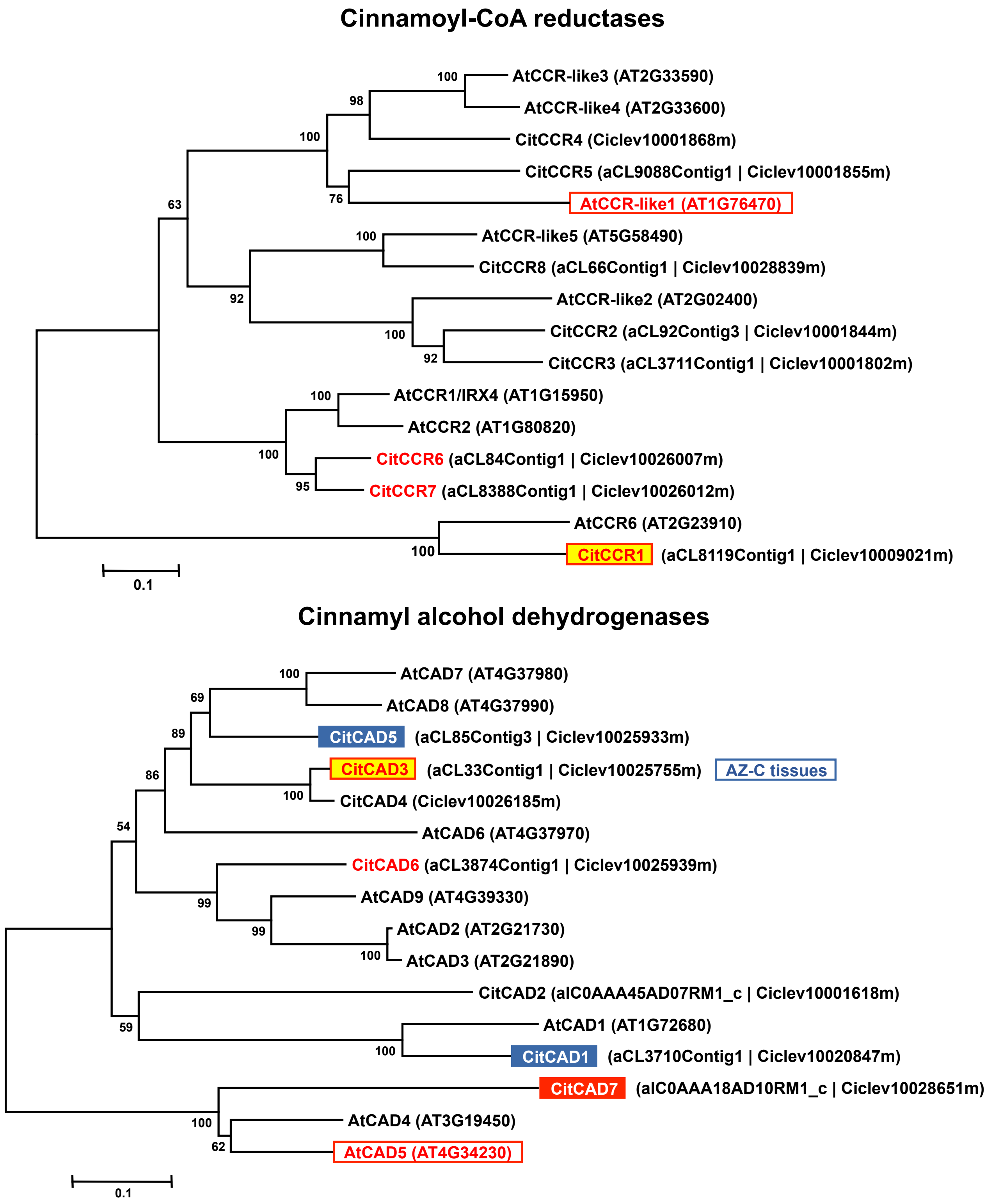

Figure S7H. Phylogenetic relationships between cinnamoyl-CoA reductase (CCRs) and cinnamyl alcohol dehydrogenase (CADs). This oxido-reductases are employed in the core monolignol biosynthetic pathway that reduces hydroxycinnamoyl-CoA esters to their corresponding alcohols. CCRs catalyze the reduction of 4-coumaroyl- and feruloyl-CoA to 4-coumaraldehyde and coniferaldehyde whereas CADs catalyze the final reduction of the cinnamaldehydes to the corresponding alcohols. Organ abscission is regulated by plant physiological and developmental status, and biotic and abiotic environmental conditions (Estornell et al., 2013) and molecular analysis shows that *AtCCR-like1* and *AtCAD5* are up-regulated in Arabidopsis stamen AZ cells during stamen abscission (Cai and Lashbrook, 2008). The CCR and CAD gene families in citrus apparently consist of eight and seven members, respectively. *CitCCR1* (aCL8119Contig1|Ciclev10009021m) and *CitCAD3* (aCL33Contig1|Ciclev10025755m) are exclusively regulated (up-regulated) in AZ-C cells during ethylene-promoted abscission. In a recent transcriptomic survey on citrus abscission zone-enriched tissues, *CitCAD3* was down-regulated by treatment with ethephon (Cheng et al., 2015).

Coniferaldehyde/sinapaldehyde dehydrogenase

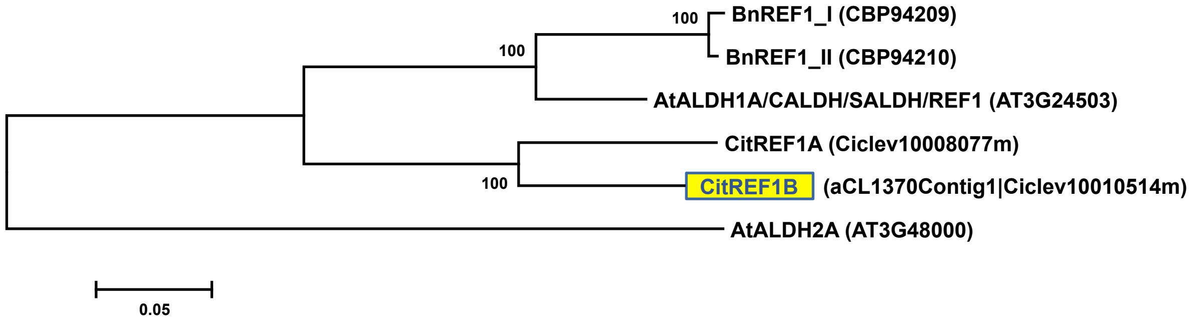

Figure S7I. Phylogenetic relationships between aldehyde dehydrogenase/coniferaldehyde dehydrogenase/sinapaldehyde dehydrogenase (ALDH/CALDH/SALDH). The bifunctional enzyme ALDH/CALDH/SALDH catalyzes the NADP+-dependent oxidation of coniferaldehyde and sinapaldehyde to yield the corresponding hydroxycinnamates, ferulate and sinapate. As a potential branching enzyme, the enzymatic activity of the REF1-encoded ALDH/CALDH/SALDH might be crucial for the partition ratio of metabolites between lignin and hydroxycinnamate biosynthesis (Nair et al., 2004; Mittasch et al., 2013). There are two REF1-type genes in citrus and *CitREF1B* (aCL1370Contig1|Ciclev10010514m) is regulated (down-regulated) in exclusively in AZ-C cells during ethylene-promoted abscission. This result strongly suggest that monolignol biosynthesis is favored during fruit abscission through down-regulation of hydroxycinnamate biosynthesis.

Coniferyl/sinapyl alcohol glucosyltransferases

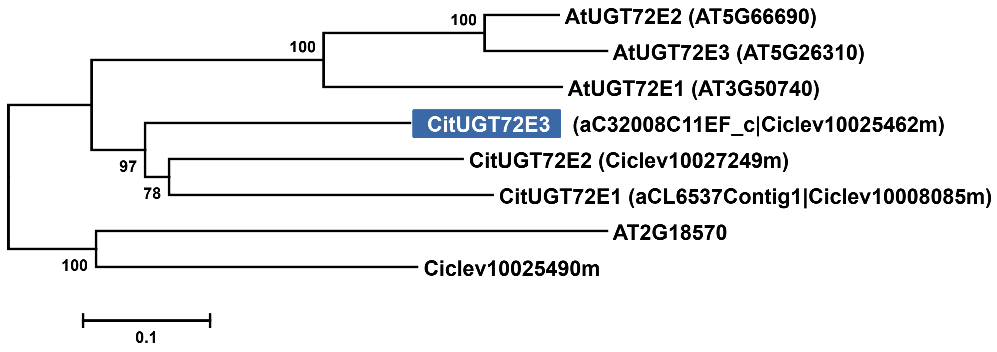

Figure S7J. Putative involvement of monolignol glucosides in the lignin biosynthesis pathway. It has been proposed that monolignols are stored in the vacuole and transported to the cell wall as glucosides and then released for oxidation and polymerization by the action of *β*-glucosidases (BGLU; Liu, 2012). In Arabidopsis, the hydrolysis of monolignol glucosides would involve three BGLUs (AtBGLU45, AtBGLU46 and AtBGLU47) belonging to the group 10 of Family 1 glycoside hydrolases (Xu et al., 2004; Escamilla-Treviño et al., 2006; Chapelle et al., 2012). The only report in the literature supporting a putative involvement of monolignol *β*-glucosidases in organ abscission comes from the down-regulation of *AtBGLU46* in the floral receptacles of Arabidopsis *ida-2* (Liu et al, 2013) and double *hae hsl2* mutant plants (Niederhuth et al., 2013). The citrus homologs of these monolignol BGLUs are not regulated by ethylene in the fruit AZ during abscission (Cheng et al., 2015; see Supplemental Figure S4 in this manuscript). Regarding the glucosylation of monolignols, it involves the activity of specific UDP-glucosyltransferases, the coniferyl/sinapyl alcohol glucosyltransferases (Steeves et al., 2001). In Arabidopsis, a small cluster of three closely related UDP-glucosyltransferases, UGT72E1-E3, is capable of glucosylate monolignols *in vitro* (Lim et al., 2005; Lanot et al., 2006). In the genome of citrus there are also three coniferyl/sinapyl alcohol glucosyltransferases and only one of them, *CitUGT72E3*, is regulated (down-regulated) by ethylene in both AZ-C and fruit rind cells during fruit abscission. Thus, these results apparently suggest that monolignol glucosides are not involved in citrus fruit abscission.

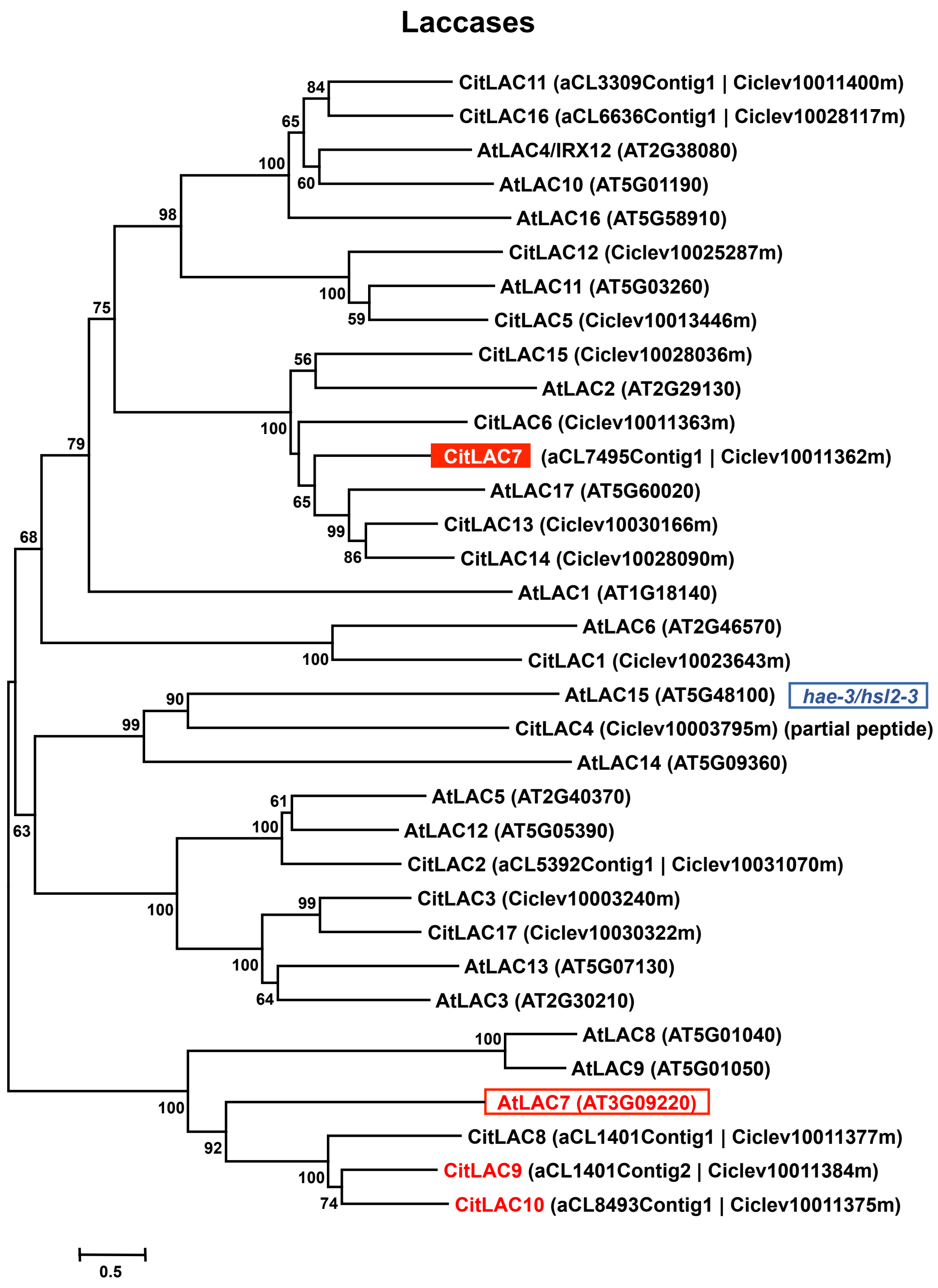

Figure S7K. Phylogenetic relationships between laccases (LACs). Monolignols are synthesized in the cytoplasm and translocated to the cell wall for subsequent polymerization (Alejandro et al., 2012). Recent biochemical studies strongly suggest that monolignols are transported into the cell wall across the plasma membrane in an ATP- dependant process by an ABC-transporter (see Supplemental Figures S8F and S8G) and is subsequently polymerized by laccases (LACs) and/or peroxidases (PRXs) (Alejandro et al., 2012). The incorporation of monolignols into the lignin polymer by PRXs and a specific NADPH oxidase is facilitated, at least in the Casparian strips of Arabidopsis roots, through the action of Casparian strip domain proteins (CASPs) (Lee et al., 2013). Organ abscission is regulated by plant physiological and developmental status, and biotic and abiotic environmental conditions (Estornell et al., 2013). Molecular analysis shows that *AtLAC7* is up-regulated in stamen abscission zone cells during stamen abscission (Cai and Lashbrook, 2008) and *AtLAC15* is down-regulated in the floral receptacles of double *hae hsl2* mutant plants (Niederhuth et al., 2013) suggesting that this gene may be up-regulated in floral organ abscission zones during abscission. The LAC gene family in citrus consist of seventeen members and only *CitLAC7* (aCL7495Contig1|Ciclev10011362m) is regulated (up-regulated) in both AZ-C and FR cells during ethylene-promoted abscission.

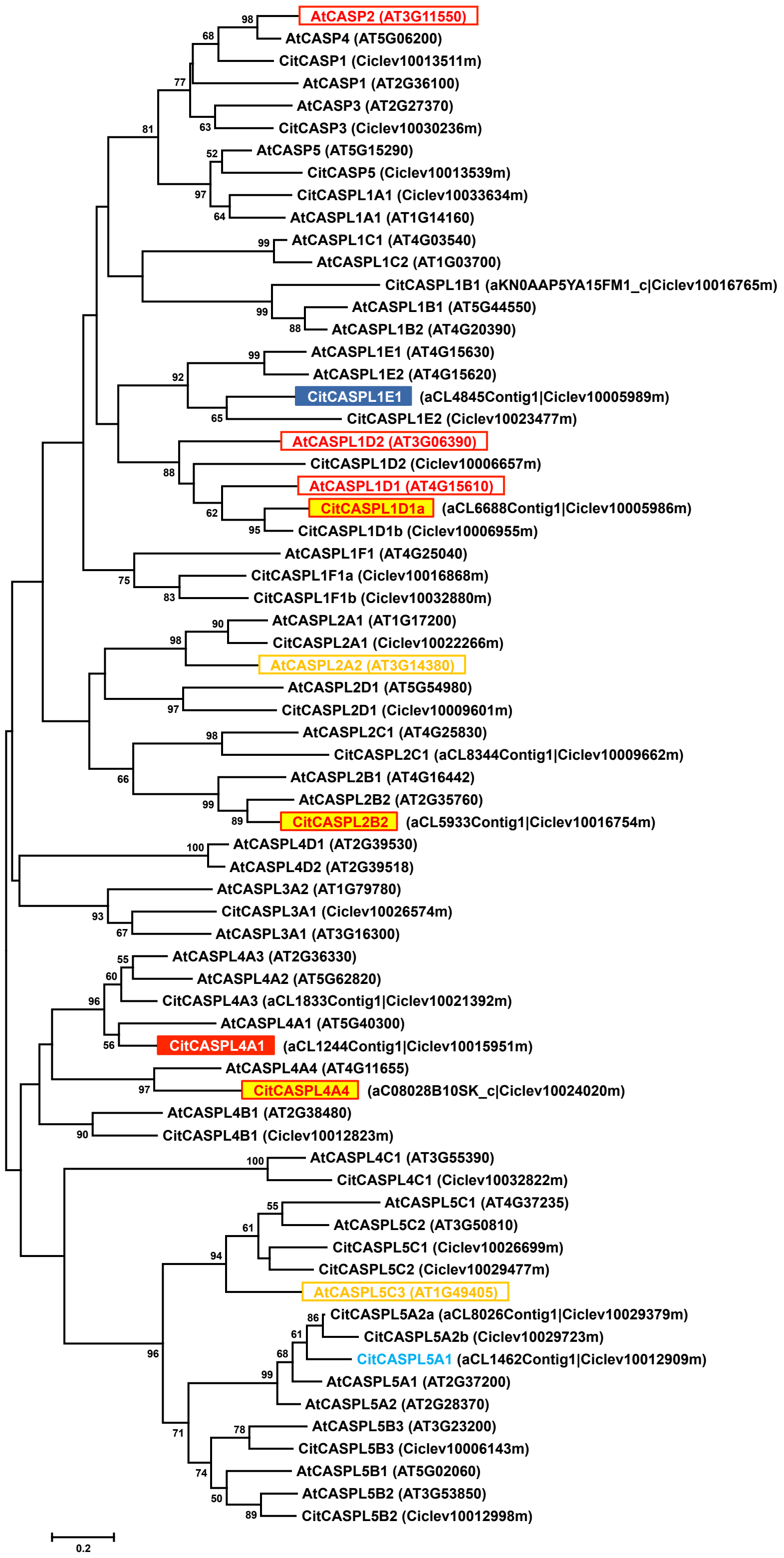

Figure S7L. Phylogenetic relationships between CASPARIAN STRIP MEMBRANE DOMAIN PROTEIN-like (CASPLs). CASPLs are four-membrane-span proteins that mediate the deposition of Casparian strips in the root endodermis by recruiting the lignin polymerization machinery (Roppolo et al., 2011, 2014). In Arabidopsis, CASPLs show specific expression in a variety of cell types including floral organs abscission zone cells (*AtCASPL2A2* and *AtCASPL5C3*; Roppolo et al, 2014). In relation to this suggesting observation, three CASPLs are up-regulated in stamen abscission zone cells during stamen abscission (Cai and Lashbrook, 2008). One of them, *AtCASPL1D1*, shows a close phylogenetic relationship with *CitCASPL1D1a* (aCL6688Contig1|Ciclev10005986m) that is regulated (up-regulated) exclusively in AZ-C cells during ethylene-promoted abscission. Another two citrus CASPLs, CitCASPL4A4 (aC08028B10SK_c|Ciclev10024020m) and CitCASPL2B2 (aCL5933Contig1|Ciclev10016754m), are also up-regulated exclusively in AZ-C cells.

REFERENCES

Alejandro, S., Lee, Y., Tohge, T., Sudre, D., Osorio, S., Park, J., Bovet, L., Lee, Y., Geldner, N., Fernie, A.R., and Martinoia, E. (2012). AtABCG29 is a monolignol transporter involved in lignin biosynthesis. *Curr Biol* 22, 1207-1212.

Bassard, J.E., Richert, L., Geerinck, J., Renault, H., Duval, F., Ullmann, P., Schmitt, M., Meyer, E., Mutterer, J., Boerjan, W., De Jaeger, G., Mely, Y., Goossens, A., and Werck-Reichhart, D. (2012). Protein-protein and protein-membrane associations in the lignin pathway. *Plant Cell* 24, 4465-4482.

Bell-Lelong, D.A., Cusumano, J.C., Meyer, K., and Chapple, C. (1997). Cinnamate-4-hydroxylase expression in Arabidopsis. Regulation in response to development and the environment. *Plant Physiol* 113, 729-738.

Cai, S., and Lashbrook, C.C. (2008). Stamen abscission zone transcriptome profiling reveals new candidates for abscission control: enhanced retention of floral organs in transgenic plants overexpressing Arabidopsis ZINC FINGER PROTEIN2. *Plant Physiol* 146, 1305-1321.

Chapelle, A., Morreel, K., Vanholme, R., Le-Bris, P., Morin, H., Lapierre, C., Boerjan, W., Jouanin, L., and Demont-Caulet, N. (2012). Impact of the absence of stem-specific beta-glucosidases on lignin and monolignols. *Plant Physiol* 160, 1204-1217.

Chen, H.C., Li, Q., Shuford, C.M., Liu, J., Muddiman, D.C., Sederoff, R.R., and Chiang, V.L. (2011). Membrane protein complexes catalyze both 4- and 3-hydroxylation of cinnamic acid derivatives in monolignol biosynthesis. *Proc Natl Acad Sci U S A* 108, 21253-21258.

Cheng, C., Zhang, L., Yang, X., and Zhong, G. (2015). Profiling gene expression in citrus fruit calyx abscission zone (AZ-C) treated with ethylene. *Mol Genet Genomics* 290, 1991-2006.

Ehlting, J., Mattheus, N., Aeschliman, D.S., Li, E., Hamberger, B., Cullis, I.F., Zhuang, J., Kaneda, M., Mansfield, S.D., Samuels, L., Ritland, K., Ellis, B.E., Bohlmann, J., and Douglas, C.J. (2005). Global transcript profiling of primary stems from Arabidopsis thaliana identifies candidate genes for missing links in lignin biosynthesis and transcriptional regulators of fiber differentiation. *Plant J* 42, 618-640.

Escamilla-Trevino, L.L., Chen, W., Card, M.L., Shih, M.C., Cheng, C.L., and Poulton, J.E. (2006). Arabidopsis thaliana beta-Glucosidases BGLU45 and BGLU46 hydrolyse monolignol glucosides. *Phytochemistry* 67, 1651-1660.

Estornell, L.H., Agusti, J., Merelo, P., Talon, M., and Tadeo, F.R. (2013). Elucidating mechanisms underlying organ abscission. *Plant Sci* 199-200, 48-60.

Jensen, K., and Moller, B.L. (2010). Plant NADPH-cytochrome P450 oxidoreductases. *Phytochemistry* 71, 132-141.

Lanot, A., Hodge, D., Jackson, R.G., George, G.L., Elias, L., Lim, E.K., Vaistij, F.E., and Bowles, D.J. (2006). The glucosyltransferase UGT72E2 is responsible for monolignol 4-O-glucoside production in Arabidopsis thaliana. *Plant J* 48, 286-295.

Lee, Y., Rubio, M.C., Alassimone, J., and Geldner, N. (2013). A mechanism for localized lignin deposition in the endodermis. *Cell* 153, 402-412.

Lim, E.K., Jackson, R.G., and Bowles, D.J. (2005). Identification and characterisation of Arabidopsis glycosyltransferases capable of glucosylating coniferyl aldehyde and sinapyl aldehyde. *FEBS Lett* 579, 2802-2806.

Liu, B., Butenko, M.A., Shi, C.L., Bolivar, J.L., Winge, P., Stenvik, G.E., Vie, A.K., Leslie, M.E., Brembu, T., Kristiansen, W., Bones, A.M., Patterson, S.E., Liljegren, S.J., and Aalen, R.B. (2013). NEVERSHED and INFLORESCENCE DEFICIENT IN ABSCISSION are differentially required for cell expansion and cell separation during floral organ abscission in Arabidopsis thaliana. *J Exp Bot* 64, 5345-5357.

Liu, C.J. (2012). Deciphering the enigma of lignification: precursor transport, oxidation, and the topochemistry of lignin assembly. *Mol Plant* 5, 304-317.

Martinez-Godoy, M.A., Mauri, N., Juarez, J., Marques, M.C., Santiago, J., Forment, J., and Gadea, J. (2008). A genome-wide 20 K citrus microarray for gene expression analysis. *BMC Genomics* 9, 318.

Mittasch, J., Böttcher, C., Frolov, A., Strack, D., and Milkowski, C. (2013). Reprogramming the Phenylpropanoid Metabolism in Seeds of Oilseed Rape by Suppressing the Orthologs of REDUCED EPIDERMAL FLUORESCENCE1. *Plant Physiology* 161, 1656-1669.

Nair, R.B., Bastress, K.L., Ruegger, M.O., Denault, J.W., and Chapple, C. (2004). The Arabidopsis thaliana REDUCED EPIDERMAL FLUORESCENCE1 gene encodes an aldehyde dehydrogenase involved in ferulic acid and sinapic acid biosynthesis. *Plant Cell* 16, 544-554.

Nair, R.B., Xia, Q., Kartha, C.J., Kurylo, E., Hirji, R.N., Datla, R., and Selvaraj, G. (2002). Arabidopsis CYP98A3 mediating aromatic 3-hydroxylation. Developmental regulation of the gene, and expression in yeast. *Plant Physiol* 130, 210-220.

Niederhuth, C.E., Patharkar, O.R., and Walker, J.C. (2013). Transcriptional profiling of the Arabidopsis abscission mutant hae hsl2 by RNA-Seq. *BMC Genomics* 14, 37.

Ohl, S., Hedrick, S.A., Chory, J., and Lamb, C.J. (1990). Functional properties of a phenylalanine ammonia-lyase promoter from Arabidopsis. *Plant Cell* 2, 837-848.

Raes, J., Rohde, A., Christensen, J.H., Van De Peer, Y., and Boerjan, W. (2003). Genome-wide characterization of the lignification toolbox in Arabidopsis. *Plant Physiol* 133, 1051-1071.

Roppolo, D., Boeckmann, B., Pfister, A., Boutet, E., Rubio, M.C., Denervaud-Tendon, V., Vermeer, J.E., Gheyselinck, J., Xenarios, I., and Geldner, N. (2014). Functional and Evolutionary Analysis of the CASPARIAN STRIP MEMBRANE DOMAIN PROTEIN Family. *Plant Physiol* 165, 1709-1722.

Roppolo, D., De Rybel, B., Denervaud Tendon, V., Pfister, A., Alassimone, J., Vermeer, J.E., Yamazaki, M., Stierhof, Y.D., Beeckman, T., and Geldner, N. (2011). A novel protein family mediates Casparian strip formation in the endodermis. *Nature* 473**,** 380-383.

Soitamo, A.J., Piippo, M., Allahverdiyeva, Y., Battchikova, N., and Aro, E.-M. (2008). Light has a specific role in modulating Arabidopsis gene expression at low temperature. *BMC Plant Biology* 8**,** 13.

Steeves, V., Forster, H., Pommer, U., and Savidge, R. (2001). Coniferyl alcohol metabolism in conifers. I. Glucosidic turnover of cinnamyl aldehydes by UDPG: coniferyl alcohol glucosyltransferase from pine cambium. *Phytochemistry* 57**,** 1085-1093.

Sundin, L., Vanholme, R., Geerinck, J., Goeminne, G., Hofer, R., Kim, H., Ralph, J., and Boerjan, W. (2014). Mutation of the inducible ARABIDOPSIS THALIANA CYTOCHROME P450 REDUCTASE2 alters lignin composition and improves saccharification. *Plant Physiol* 166**,** 1956-1971.

Urban, P., Mignotte, C., Kazmaier, M., Delorme, F., and Pompon, D. (1997). Cloning, yeast expression, and characterization of the coupling of two distantly related Arabidopsis thaliana NADPH-cytochrome P450 reductases with P450 CYP73A5. *J Biol Chem* 272**,** 19176-19186.

Varadarajan, J., Guilleminot, J., Saint-Jore-Dupas, C., Piegu, B., Chaboute, M.E., Gomord, V., Coolbaugh, R.C., Devic, M., and Delorme, V. (2010). ATR3 encodes a diflavin reductase essential for Arabidopsis embryo development. *New Phytol* 187**,** 67-82.

Xu, Z., Escamilla-Trevino, L., Zeng, L., Lalgondar, M., Bevan, D., Winkel, B., Mohamed, A., Cheng, C.L., Shih, M.C., Poulton, J., and Esen, A. (2004). Functional genomic analysis of Arabidopsis thaliana glycoside hydrolase family 1. *Plant Mol Biol* 55**,** 343-367.

Zhang, X., and Liu, C.-J. Multifaceted Regulations of Gateway Enzyme Phenylalanine Ammonia-Lyase in the Biosynthesis of Phenylpropanoids. *Molecular Plant* 8**,** 17-27.
